# Design, Synthesis, and Pharmacological Activity of the *N*-(4-(1H-1,2,4-Triazol-1-yl)phenyl)-substituted-amide Derivatives

**DOI:** 10.3390/molecules30163400

**Published:** 2025-08-18

**Authors:** Lina Hu, Mengjiao Li, Zheng Liu, Hui Yan, Xuekun Wang, Shiben Wang

**Affiliations:** 1 State Key Laboratory of Macromolecular Drugs and Large-Scale Preparation, School of Pharmaceutical Sciences and Food Engineering, Liaocheng University, Liaocheng 252059, China; 2019207801@stu.lcu.edu.cn (L.H.); 2210240102@stu.lcu.edu.cn (M.L.); yanhui@lcu.edu.cn (H.Y.); 2 School of Medicine, Foshan University, Foshan 528000, China; liuzheng8709@fosu.edu.cn

**Keywords:** synthesis, triazole, anticonvulsant, GABA, molecular docking

## Abstract

To discover novel, potent anticonvulsant compounds, a series of compounds containing triazole structural fragments were synthesized and evaluated for their anticonvulsant activity. Compounds **6f** (maximal electroshock (MES), 50% effective dose (ED_50_) = 13.1 mg/kg; subcutaneous pentylenetetrazole (scPTZ), ED_50_ = 19.7 mg/kg) and **6l** (MES, ED_50_ = 9.1 mg/kg; *sc*PTZ, ED_50_ = 19.0 mg/kg) showed the best activity in MES and *sc*PTZ tests. The results of an affinity test showed that the potent compounds had better binding to the GABA_A_ receptors. The results of an inhibitory neurotransmitter GABA content test in brain tissue indicated that compounds **6f** and **6l** exerted anticonvulsant activity which may be associated with the increase of GABA content in the rat brain. Elevated plus maze (EPM) assay results showed that compounds **6f** and **6l** possessed anxiolytic effects, and their effects correlated with the binding sites of benzodiazepines (BZs) in the GABA_A_ receptors. Molecular docking was performed to investigate the interactions of the studied compound **6l** with the GABA_A_ receptors on the molecular level.

## 1. Introduction

Epilepsy is a clinical syndrome caused by highly synchronized abnormal discharges of neurons in the brain due to a variety of reasons, and is a chronic brain disease. Epilepsy not only causes serious distress in the lives of patients, but is also one of the global public health problems. Epilepsy can develop at any age and, according to incomplete statistics, epilepsy affects more than 70 million people worldwide [1,2,3]. The treatment of epilepsy includes medication, surgery, and ketogenic diet, among other methods. Currently, medication is the main method of epilepsy treatment, which can effectively control or reduce the number of seizures in epilepsy patients. Currently, there are more than 20 anticonvulsant drugs used in clinical practice, and hundreds of combination methods of these drugs will appear as combination drugs, which are often prone to problems, such as toxic effects of drugs, deterioration of patient compliance, and higher economic burden on families; about 30% of patients are resistant to the selected anticonvulsant drugs, which makes it difficult to achieve the expected results [4,5,6]. Although, new anticonvulsant drugs appear in the clinic every year, it is still difficult to meet the needs of epileptic patients. Therefore, it is important to design and develop anticonvulsant drugs with novel structures.

**Figure 1:**
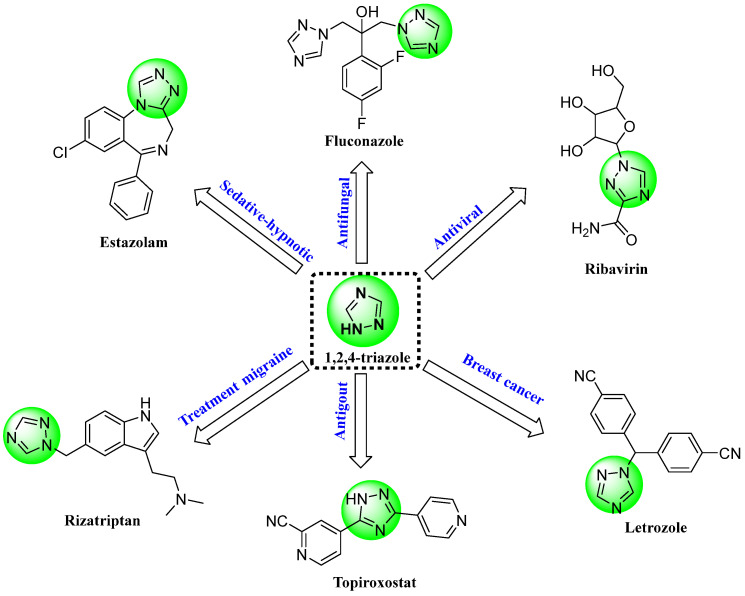
Drugs containing 1,2,4-triazole.

The compound 1,2,4-triazole is a common five-membered aromatic heterocyclic structure in drug molecules and medicinal chemistry design with weak basicity, which is an important intermediate in the synthesis of pesticide fungicides, plant growth regulators polyazole, and alizarinol. As a very valuable five-membered nitrogen-containing heterocyclic skeleton, 1,2,4-triazole has also been widely used in many bioactive molecules, such as in antimicrobial [7], anti-inflammatory [8], antiproliferative activity [9], neuroprotectivity [10], antidiabetic [11], antitumor [12], and anticonvulsant [13,14], among other drug development areas. There are many drugs containing 1,2,4-triazole structural fragments currently on the market, including estazolam (sedative-hypnotic), fluconazole (antifungal), ribavirin (antiviral), and letrozole (breast cancer), among others (Figure 1). Triazolam, the first anticonvulsant drug discovered with a structural fragment of triazole, also has a sedative-hypnotic effect. Nowadays, more and more medicinal chemists are beginning to use triazole structural fragments in the design and development of anticonvulsant drugs [15,16,17,18].

**Figure 2:**
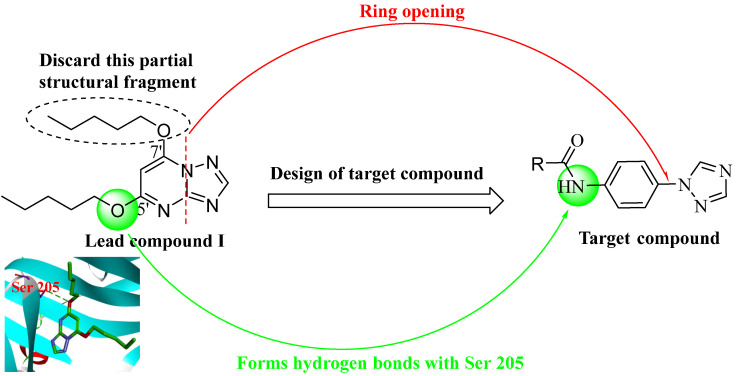
Chemical structures of lead compound **I** and target compounds.

In our previous study, the group synthesized a series of compounds containing the triazole structure (compound **I** is the best active compound, Figure 2) and evaluated their anticonvulsant activity. The pharmacological results showed that this series of compounds had good anticonvulsant activity [14]. Previously, molecular docking simulations revealed that the “O” atom at the 5-position in the structure of compound **I** can form important hydrogen bonding interactions with Ser205, an important amino acid in the active site of benzodiazepines (BZs) of the GABA_A_ receptor (PDB Code: 6HUP, obtained from RCSB), whereas the alkyl chain attached to the “O” atom forms hydrophobic interactions with the active site amino acids. The alkyl chain attached to the “O” atom at the 7-position in the structure of compound **I** cannot form interactions with the active site amino acids. The triazolopyrimidine structure of compound **I** can form pi–pi stacks interaction with the active site amino acids, such as Phe77. Based on the above findings, the present work was carried out to design new compounds using compound **I** as the lead compound. The design consists of the following three aspects: (1) the “O” atom in the lead compound was replaced by an amide structure that can form hydrogen bonds and is important for improving biological activity; (2) the alkyl chain fragment at the 7-position that does not interact with the active site was discarded; (3) the triazolopyrimidine structure was modified to a ring-opening structure to explore the effect of the ring-opening structure on the activity. In summary, a series of derivatives containing the triazole structure were synthesized by using 4-bromoaniline and other synthetic raw materials, expecting to obtain potential compounds with better activity, and further exploring their structural relationships.

**Figure 3:**
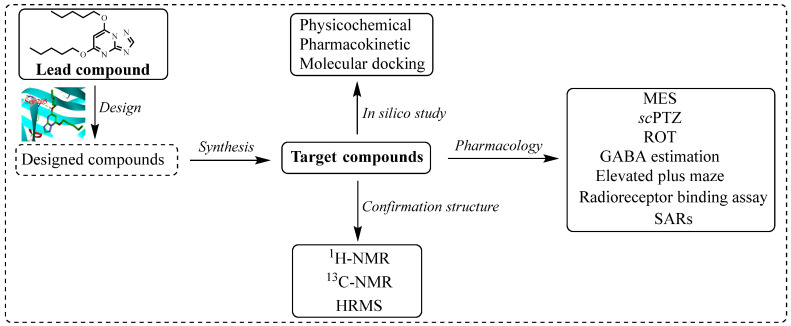
Experimental protocol.

In this study, the synthesized compounds were tested for structure confirmation and pharmacological activity experiments. The specific experimental scheme is shown in Figure 3. The pre-discovered compound **I** was used as the lead compound for structural modification. For the structure confirmation part, the ^1^H NMR, ^13^C NMR, and HRMS methods were used for verification. In the pharmacological experimental, the target compounds were initially screened for anticonvulsant activity and neurotoxicity using maximal electroshock (MES), subcutaneous pentylenetetrazole (scPTZ), and rotating rod (ROT) in vivo animal experimental models. The potential compounds were selected to determine the affinity with the GAAB_A_ receptors, and were further quantitatively evaluated for their 50% effective dose (ED_50_) and 50% toxic dose (TD_50_). The optimal compounds with good activity, low toxicity, and high safety were finally selected. The possible mode of action of the optimal compounds to exert activity was further verified using the brain tissue GABA content assay and the elevated plus maze (EPM) assay. Discovery Studio 2021 (DS 2021) was used for molecular docking simulations to predict the interaction force of the target compounds with the GABA_A_ receptors and to analyze the possible mode of action with the receptors. DS 2021 software was used to predict the physicochemical and pharmacokinetic properties of the target compounds and to further validate the potential for their druggability.

## 2. Results and Discussion

### 2.1. Chemistry

**Scheme 1:**
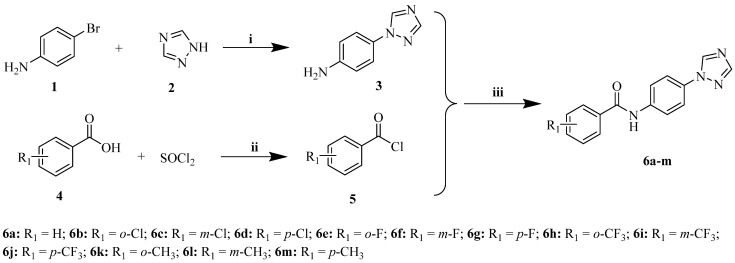
Synthesis of target compounds **6a**–**m**. Reagents and conditions: (**i**) CuI, CsCO_3_, DMF, 120 °C, 40 h; (**ii**) SOCl_2_, CH_2_Cl_2_, 30 °C, 6 h; (**iii**) Et_3_N, CH_2_Cl_2_, room temperature, 2 h.

**Scheme 2:**
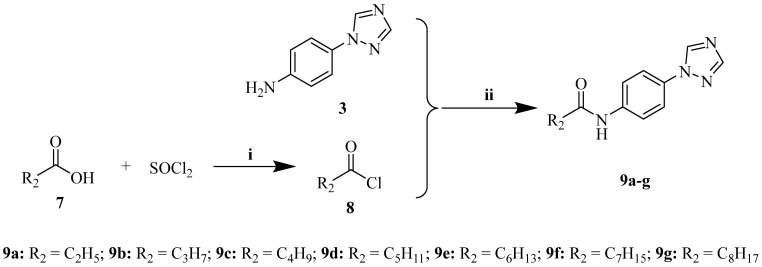
Synthesis of target compounds **9a**–**g**. Reagents and conditions: (**i**) SOCl_2_, CH_2_Cl_2_, 30 °C, 6 h; (**ii**) Et_3_N, CH_2_Cl_2_, room temperature, 2 h.

The synthetic route for the target compounds **6a**–**m** and **9a**–**g** is shown in Scheme 1 and Scheme 2. Firstly, 4-bromoaniline (compound **1**) was used as a starting material and reacted with 1,2,4-triazole (compound **2**) under alkaline conditions to obtain the intermediate 4-(1H-1,2,4-triazol-1-yl)aniline (compound **3**) [19]. Compound **3** was then reacted with various substituted benzoyl chlorides (compound **5**) under alkaline conditions to give the target compounds **6a**–**m**, and with intermediate compound **8** obtained after fatty acid chlorination to give the target compounds **9a**–**g**. A total of twenty target compounds were synthesized in the experiments, all of which were subjected to structural confirmation by using the ^1^H NMR, ^13^C NMR, and HRMS methods.

A structural analysis of the target compound **6a**–**m** produced the following results: such as *N*-(4-(1H-1,2,4-triazol-1-yl)phenyl)benzamide (**6a**), in ^1^H NMR, the multiple peaks at δ of 7.54–8.00 ppm correspond to the benzene ring protons, the single peaks at δ of 8.23 and 9.25 ppm are the peaks of H on the triazole (2H), and the single peak at δ 10.32 ppm is the peak of NH on amide (1H). The ^13^C NMR spectrum accurately gave various information about the structure of compound **6a**, such as the peaks at δ 120.34–139.22 ppm are the carbon of the benzene ring, the peaks at δ 142.47, 152.72 ppm are the carbon of the methylene group, and the peak at δ 166.17 ppm is the carbon of the carbonyl group. In the HRMS analysis, the theoretical molecular weight of compound **6a** was 265.1084, and the actual measured value was 265.1090, with a difference of less than 0.001. The molecular structure could be confirmed based on the H and C spectra.

### 2.2. Pharmacology

#### 2.2.1. MES and *sc*PTZ

Pharmacological tests were performed using two experimental animal models, MES and *sc*PTZ, which were divided into three dosage groups of 30, 100, and 300 mg/kg, and the anticonvulsant activity of the target compounds **6a**–**m** and **9a**–**g**, after 0.5 and 4 h of administration, respectively, were tested. Carbamazepine, which is effective in the MES model, was chosen as a positive control, and ethosuximide, which is effective in the *sc*PTZ model, was chosen as a positive control. The results of the pharmacological experiments for MES and *sc*PTZ are shown in Table 1.

From the results of the MES experiments presented in Table 1, it can be seen that all the target compounds showed anticonvulsant activity at different doses after 0.5 h of administration. For example, compounds **6a**–**b**, **6d**, **6m**, and **9g** showed anticonvulsant activity at a dose of 300 mg/kg; compounds **6c**, **6e**, **6h**, **6j**, **9a**, and **9e**–**f** showed anticonvulsant activity at a dose of 100 mg/kg; whereas compounds **6f**–**g**, **6i**, **6k**–**l**, and **9b**–**d** showed the best anticonvulsant activity at a dose of 30 mg/kg. After 4 h of administration, all the target compounds showed anticonvulsant activity, while seven compounds **6f**–**g**, **6i**, **6k**–**l**, and **9c**–**d** showed the best anticonvulsant activity at a dose of 30 mg/kg.

It can be seen from the *sc*PTZ test results in Table 1 that, after 0.5 h of administration, all compounds showed anticonvulsant activity at 100 and 300 mg/kg dose except for six compounds, **6f**–**g**, **6i**, **6l**, and **9b**–**c**, which showed the best activity at 30 mg/kg dose. After 4 h of administration, all compounds showed anticonvulsant activity except for five compounds, **6d**, **6h**, and **9e**–**g**, which were inactive at 30, 100, and 300 mg/kg dose, while compounds **6f** and **6l** showed the best activity at 30 mg/kg.

#### 2.2.2. ROT

The neurotoxicity of the target compounds was tested using the ROT experimental model, which was divided into two dose groups, 100 and 300 mg/kg, and tested after 0.5 and 4 h of administration, respectively. The results of the ROT test are shown in Table 2. As can be seen from Table 2, after 0.5 h of administration, none of the compounds were neurotoxic except for compound **9b**–**c**, which showed neurotoxicity at a dose of 300 mg/kg. After 4 h of administration, none of the compounds were neurotoxic.

#### 2.2.3. ED_50/_TD_50_ Determination

In order to quantitatively evaluate the ED_50_ (0.5 h) and TD_50_ (0.5 h) of the potential compounds and to calculate the protective index (PI = TD_50_/ED_50_), five potential compounds, **6f**–**g**, **6i**, **6l** and **9c**, were selected from Table 1 and Table 2 for testing. Carbamazepine was selected as a positive control in the MES test, and ethosuximide was selected as a positive control in the *sc*PTZ test. The results of the tests and analyses are shown in Table 3.

From the analytical results in Table 3, it can be seen that, in the MES test, compound **6l** showed the lowest ED_50_ value of 9.1 mg/kg, which showed the best activity, and was superior to the positive control carbamazepine at 11.4 mg/kg; whereas, in the *sc*PTZ test, compound **6f** showed the lowest ED_50_ value of 19.7 mg/kg, which showed the best activity, and was superior to the positive control ethosuximide of 94.4 mg/kg; while, in ROT test, compound **6f** showed the highest TD_50_ value of 476.7 mg/kg, and showed the lowest neurotoxicity. In the MES test, the PI values of compounds **6f** and **6l** were 36.3 and 45.9, respectively, which were higher than that of the positive control carbamazepine (6.1) in terms of safety. In the *sc*PTZ test, the PI values of compounds **6f** and **6l** were 24.2 and 22.1, respectively, which were higher than that of the positive control ethosuximide (3.5). In conclusion, compounds **6f** and **6l** are the most preferred compounds for further study.

#### 2.2.4. Radioreceptor Binding Assay

In order to verify the possible mechanism of action of this series of compounds to exert their activity, five potential compounds, **6f**–**g**, **6i**, **6l**, and **9c**, were selected from Table 1 and Table 2 and were tested for their affinities with the GABA_A_ receptor; the binding site is the active site of BZs of the GABA_A_ receptor. Diazepam was selected as a positive control. The results are shown in Table 4. As can be seen from Table 4, compound **6f** showed the best affinity with a half-maximal inhibitory concentration (IC_50_) of 0.14 µM, and the binding was weaker than the positive control diazepam, with an IC_50_ of 0.034 µM. Other compounds also showed different levels of affinity to the GABA_A_ receptor. The results suggest that the compounds in this series may exert their pharmacological effects by acting on the BZ binding site of the GABA_A_ receptor.

#### 2.2.5. GABA Estimation

**Figure 4:**
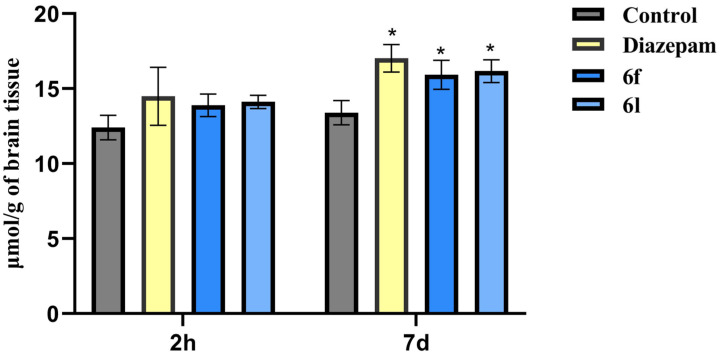
Effect of compounds **6f** and **6l** on GABA content in brain tissue. Comparisons between samples were made by Two-Way ANOVA using GraphPad Prism 8. * *p* < 0.05, significant compared to control, *n* = 5.

Gamma-aminobutyric acid (GABA) is an important inhibitory neurotransmitter in the central nervous system, and GABA has an extremely important role in the homeostatic regulation of brain function, the control of action potential issuance, and the output of neural information, as well as neural network activity and synchronization. Insufficient GABA function in the brain can cause numerous neurodegenerative disorders, such as epilepsy and convulsions that may be triggered [20]. In order to investigate whether this series of compounds affects GABA content in the brain, the two preferred compounds, **6f** and **6l**, were selected for the experiment. The experiment was carried out on Wistar rats, The compounds **6f** and **6l** were administered at a dose of 30 mg/kg, and the changes in GABA content in the brain of the rats were determined after 2 h and 7 d of administration. Diazepam was chosen as a positive control, and was administered at a dose of 3 mg/kg. The results of the tests and analyses are shown in Figure 4. In Figure 4, it can be seen that, after 2 h of administration, compounds **6f**, **6l**, and diazepam do not show significant differences (*p* > 0.05) in the brain tissue of the rats compared to the control group, but increase the content of GABA in the brain of the rats. After 7 d of continuous administration, the content of compounds **6f**, **6l**, and diazepam in the rat brain tissues showed significant differences compared to the control group (* *p* < 0.05), the results of this experiment suggest that compounds **6f** and **6l** may exert anticonvulsant effects by affecting the GABAergic system.

#### 2.2.6. EPM Test

The EPM experiment is a method for evaluating whether experimental animals have an anxiety response. The principle is that, in the face of new things, mice will generate curiosity to explore (open arm), while they have a nature of darkness (closed arm), the conflict behavior between the two occurs between exploration and avoidance, resulting in anxiety. Anxious behavior can be evaluated by comparing the retention time of mice in the open and closed arms.

**Figure 5:**
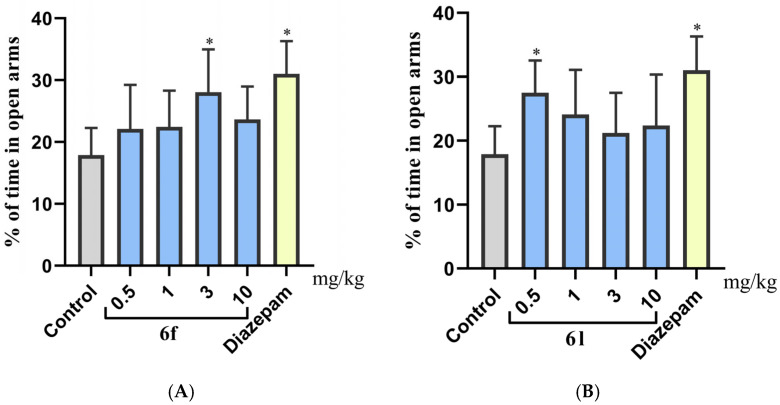
(**A**) EPM experimental results for compound **6f**; (**B**) EPM experimental results for compound **6l**. Data represent % of time in open arms = time in open arms/total time spent on all four arms, *n* = 6, * *p* < 0.05, significant compared to control.

In this experiment, compounds **6f** and **6l** were selected for the EPM tests, which were divided into four dosage groups of 0.5, 1, 3, and 10 mg/kg administration, and diazepam, which is effective in this model and has anxiolytic effects, was chosen as the positive control. The results of the EPM tests are shown in Figure 5. In Figure 5A, it can be seen that, at a dose of 3 mg/kg, compound **6f** showed a significant difference (* *p* < 0.05) compared with the control group, which significantly increased the time of the experimental animals in the open arm; compared with the control group, the diazepam (1 mg/kg) likewise showed a significant difference (* *p* < 0.05). In Figure 5B, at a dose of 0.5 mg/kg, compound **6l** showed a significant difference (* *p* < 0.05) compared to the control group.

**Figure 6:**
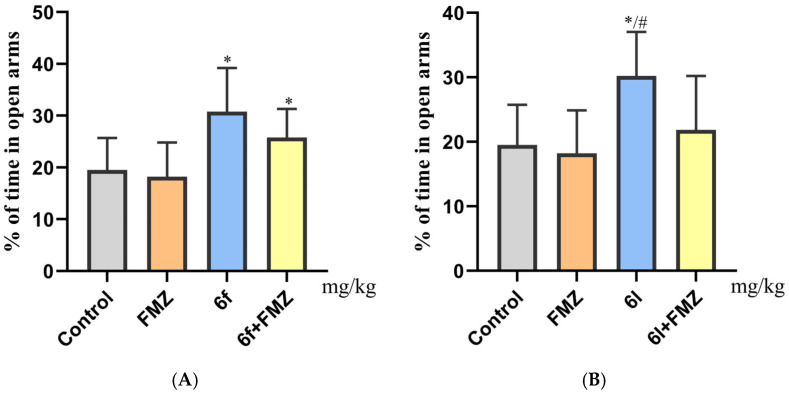
(**A**) EPM experimental results for compound **6f** and FMZ; (**B**) EPM experimental results for compound **6l** and FMZ. Data represent % of time in open arms = time in open arms/total time spent on all four arms, *n* = 6, * *p* < 0.05, significant compared to control. # *p* < 0.05, significant compared to **6f** + FMZ or **6l** + FMZ.

Flumazenil is a selective BZ antagonist with a chemical structure similar to that of BZs, which acts on central benzodiazepine (BZ) receptors and thus blocks the receptors without producing BZ-like effects. In order to verify whether compounds **6f** and **6l** exert anxiolytic effects by acting on the BZ active site of the GABA_A_ receptor, the experiment was divided into control group, FMZ group (5 mg/kg), compound group (**6f** = 3 mg/kg or **6l** = 0.5 mg/kg), and compound + FMZ group (**6f** = 3 mg/kg or **6l** = 0.5 mg/kg, and FMZ = 5 mg/kg). The results of the pharmacological experiments are shown in Figure 6. In Figure 6A, it can be seen that the FMZ group did not show significant differences compared to the control group (*p* > 0.05), while the compound **6f** group showed significant differences (* *p* < 0.05), which significantly increased the time of the experimental animals in the open arm; the **6f** + FMZ group also showed significant differences (* *p* < 0.05). The compound **6f** + FMZ group significantly shortened the time of experimental animals in open arm compared to the **6f** group. In Figure 6B, compared with the control group, the FMZ group did not show a significant difference (*p* > 0.05), the compound **6l** group showed a significant difference (* *p* < 0.05), which significantly improved the time of the experimental animals in the open arm, whereas the **6l** + FMZ group, although it did not show a significant difference, significantly shortened the time of the experimental animals in the open arm. There was a significant difference between the compound **6l** and **6l** + FMZ group (# *p* < 0.05). From the above experimental results, it is hypothesized that compounds **6f** and **6l** may exert their pharmacological effects by agonizing the BZ active site of the GABA_A_ receptor.

#### 2.2.7. Structure-Activity Relationships

In our previous study, the group designed and synthesized a series of compounds containing the triazole structure, most of the compounds showed significant anticonvulsant activity, all showed high neurotoxicity. In the present study, a series of compounds **6a**–**m** and **9a**–**g** with novel structures were synthesized by rational drug design methodology using the pre-discovered compound **I** as a lead compound. The results of the pharmacological experiments revealed that this series of compounds reduced neurotoxicity compared to the lead compounds.

From the results of the MES and *sc*PTZ experiments presented in Table 1, it was found that various electron-withdrawing and electron-donating substituents (F, Cl, CF_3_, CH_3_) and positions on the benzene ring in the compounds **6a**–**m** affect the anticonvulsant activity of the compounds. For drugs acting on the central nervous system, their lipid–water partition coefficients of a triazole structure have an important influence on the activity. In this series of compounds, the introduction of electron-withdrawing and electron-donating substituents on the benzene ring improves the lipid solubility of the target molecule, which reduces the dissociation ability of the compounds, thus making it easy to pass through the blood–brain barrier (BBB), reach the site of action, bind to the corresponding receptor, and exert the drug effect. Overall, the compounds with F and CH_3_ substitutions on the benzene ring were more active than the CF_3_-substituted compounds, while the CF_3_-substituted compounds were more active than the Cl-substituted compounds. In the two experimental models, the activity of the same substituent at different positions on the benzene ring was also different, and did not show any obvious structure–activity relationships (SARs).

**Figure 7:**
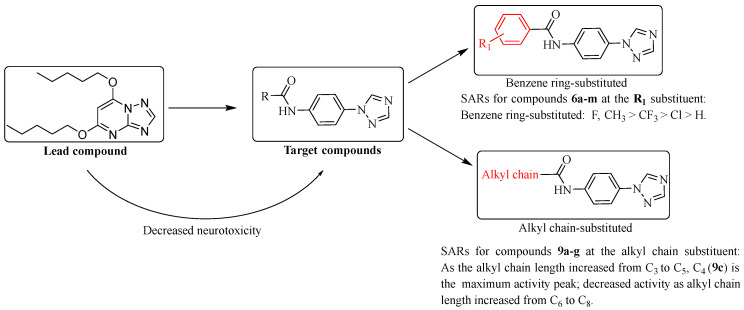
SARs of the target compounds.

In the **9a**–**g** series of alkyl chain (C_2_–C_8_) substituted derivatives, the length of the alkyl chain has an important effect on the activity. The lipid solubility of the compounds in this series is gradually enhanced with the extension of the alkyl chain, but it does not mean that the stronger the lipid solubility, the higher the activity. From the results of the MES and *sc*PTZ experiments in Table 1, it can be found that with the extension the alkyl chain (C_2_–C_4_ or **9a**–**c**), the activity is gradually enhanced, and C_4_ (**9c**) has the best activity. As the alkyl chain (C_4_–C_8_ or **9c**–**g**) continues to extend, the activity tends to decrease. The results suggest that compound **9c** has a more reasonable lipid–water partition coefficient and is more likely to actively penetrate the BBB. These preliminary structure–activity relationships provide some theoretical basis for the design of subsequent compounds (Figure 7).

#### 2.2.8. Docking Study

**Figure 8:**
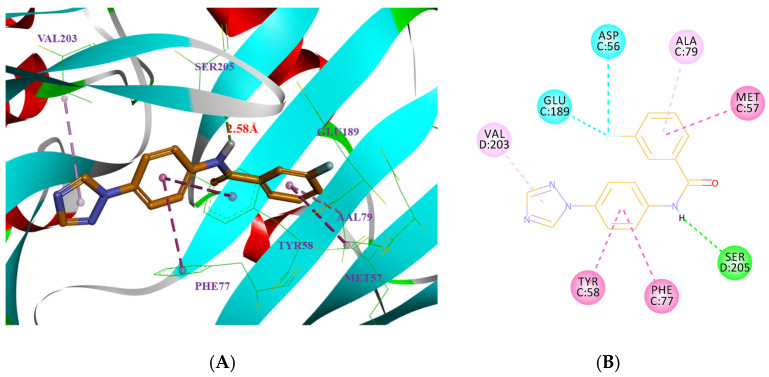
(**A**) Results of the molecular docking 3D experiments for representative compound **6l**; (**B**) Results of the molecular docking 2D experiments for representative compound **6l**.

Molecular docking is a computational method mainly used to predict the binding mode and affinity between small molecules (ligands) and biological macromolecules (proteins or receptors). In this experiment, representative compound **6l** was chosen as the ligand for docking, and the GABA_A_ receptor was chosen as the receptor for docking, the molecular docking location was the binding region of BZ in the GABA_A_ receptor, and the important amino acids around the binding region include TYR58, TYR160, TYR210, PHE77, THR207, VAL203, HIS102, SER205, and SER206, among others [21,22,23,24,25]. The molecular docking results (2D/3D plots) are shown in Figure 8.

SER205 is a very critical amino acid in the active site and is able to form an important hydrogen bonding interaction with diazepam. This hydrogen bonding interaction has a great impact on the activity of the drug. As can be seen in Figure 8, the amide group NH in the structure of compound **6l** can form hydrogen bonding interactions with SER205 at a distance of 2.58 Å. The two benzene rings of compound **6l** are able to form pi–pi interactions with the amino acids TYR58, PHE77, and MET57, and pi–alkyl interactions with the amino acids ALA79 and VAL203. In summary, we hypothesize that compound **6l**, like BZ, may act in the same way as BZ by binding to the BZ binding site of the GABA_A_ receptor and exert pharmacological effects.

**Figure 9:**
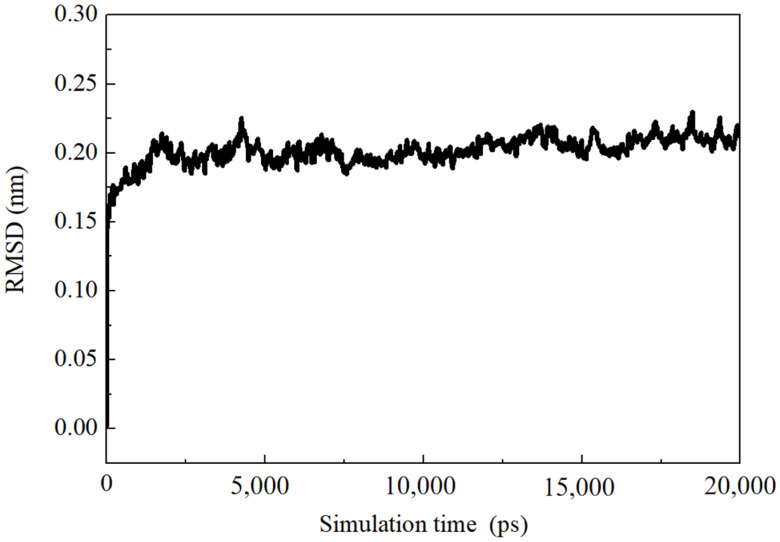
RMSD of the complex during the simulation.

To confirm the stability of the complexes formed in the case of molecular docking, molecular dynamic simulations were performed for the ligand–receptor complexes. The results are shown in Figure 9. In Figure 9, a 20 ns molecular dynamics simulation was performed, and the RMSD of the complex is shown in the figure. It can be seen that the fluctuation range is kept to within 0.2 nm, which confirms the stability of the complex.

#### 2.2.9. Prediction of Physicochemical and Pharmacokinetic Properties

The Lipinski rule of five is the most famous prediction method in the study of the rule of druggability property. Compounds that comply with the rule of five are more likely to be developed into orally consumed drugs with better bioavailability and pharmacokinetic properties in vivo. It is the most widely used indicator of druggability, which can be used to accurately predict the absorption and permeation of compounds at the early stage of drug development, and to eliminate compounds with lower feasibility as early as possible from drug discovery. The Lipinski rule of five includes molecular weight (MW) ≤ 500, number of hydrogen donors (nHD) ≤ 5, number of hydrogen acceptors (nHA) ≤ 10, number of rotatable bonds (nRotB) ≤ 10, and calculated lipophilicity (ClogP) ≤ 5. From the predicted results in Table 5, it can be seen that the physicochemical predictions of all the target compounds complied with the Lipinski rule of five. The pharmacokinetic properties of the target compounds, such as ABS level and BBB level, were also predicted in this experiment. It can be seen from the predicted results in Table 5 that all the target compounds have better pharmacokinetic properties. In summary, this series of compounds has good druggability properties.

## 3. Materials and Methods

### 3.1. Chemistry

All the chemical reagents and solvents were obtained from commercial companies (Anhui Zesun Technology Co., Ltd., Shanghai, China; Aladdin Reagent Co., Ltd., Shanghai, China) and were used without further purification. The purity of the compound was checked by TLC on plates with silica gel GF254, produced by Qingdao Haiyang Chemical Co., Ltd. (Qingdao, China). The melting points of the target compounds were determined using a micro melting point apparatus (X-4, Shanghai, China). The target compounds were dissolved in DMSO*_d6_*, and TMS was chosen as the internal standard. The ^1^H NMR and ^13^C NMR data were determined by a Bruker AV-500 nuclear magnetic resonance spectrometer (Bruker, Bremerhaven, Germany). The abbreviations for the spectral data were expressed as follows: s, single peak; d, double peaks; t, triple peaks; q, quadruple peaks; m, multiple peaks. The molecular weights of the target compounds were determined using a high-resolution mass spectrometer (Bruker Daltonik, Karlsruhe, Germany).

#### 3.1.1. Synthesis of Intermediates **5** and **8**

In a 100 mL round-bottomed reaction flask, various substituted benzoic acids/fatty acids (20.0 mmol), SOCl_2_ (60.0 mmol), and CH_2_Cl_2_ (35 mL) were added, and the reaction was carried out at 30 °C for 6 h. The reaction progress was monitored by TLC. After completion of the reaction, the solvent was removed by concentration under reduced pressure to give the crude product of intermediates **5** and **8**, which can be used in the next step of the reaction without purification.

#### 3.1.2. Synthesis of Intermediate **3**

In a 100 mL round-bottomed reaction flask, the raw materials 1,2,4-triazole (1.48 g, 20 mmol), 4-bromoaniline (3.44 g, 20 mmol), CsCO_3_ (6.84 g, 21 mmol), CuI (4 mmol), and DMF (30 mL) were added and reacted at 120 °C for 40 h. The reaction was monitored by thin-layer chromatography (TLC). After the reaction was completed, the solvent was removed by concentration under reduced pressure, 30 mL of water was added and stirred, and the crude intermediate 4-(1*H*-1,2,4-triazol-1-yl)aniline was obtained by filtration, washing with water for three times and drying, with a yield of 60%.

#### 3.1.3. General Procedure for the Synthesis of Target Compounds **6a**–**m** and **9a**–**g**

In a 100 mL round-bottomed reaction flask, 4-(1*H*-1,2,4-triazol-1-yl)aniline (4.8 g, 30.0 mmol), intermediate **5**/**8** (33.0 mmol), and CH_2_Cl_2_ (30 mL) were added, and the reaction was stirred in an ice bath for 10 min, then Et_3_N (3.0 g, 30.0 mmol) was added dropwise, the reaction was continued to be stirred at room temperature for 2 h. Thin-layer chromatography (TLC) was used to monitor the reaction process and, after the reaction was completed, the solvent was concentrated under reduced pressure, 30 mL of water was added, stirred, and then filtered, washed with water for three times, and dried to obtain the crude target compounds, which were purified by column chromatography with V (petroleum ether):V (ethyl acetate) = 10:1 to obtain the pure products. The results, such as NMR analysis, of each target compound were as follows:

*N-(4-(1H-1,2,4-triazol-1-yl)phenyl)benzamide* (**6a**): White solid, Yield 54%, Mp 235–236 °C. ^1^H NMR (500 MHz, DMSO*_d_*_6_) δ: 10.47 (s, 1H, NH), 9.25 (s, 1H, Triazole-H), 8.23 (s, 1H, Triazole-H), 8.00–7.54 (m, 9H, Ar-H). ^13^C NMR (125 MHz, DMSO*_d_*_6_) δ: 166.17, 152.72, 142.47, 139.22, 135.18, 132.82, 132.21, 128.91, 128.17, 121.57, 120.34. ESI-HRMS calcd for C_15_H_13_N_4_O^+^ ([M + H]^+^): 265.1084; found: 265.1090.

*N-(4-(1H-1,2,4-triazol-1-yl)phenyl)-2-chlorobenzamide* (**6b**): White solid, Yield 63%, Mp 247–248 °C. ^1^H NMR (500 MHz, DMSO*_d_*_6_) δ: 10.47 (s, 1H, NH), 9.25 (s, 1H, Triazole-H), 8.23 (s, 1H, Triazole-H), 8.08–7.38 (m, 8H, Ar-H). ^13^C NMR (125 MHz, DMSO*_d_*_6_) δ: 165.64, 165.04, 163.65, 152.72, 142.48, 139.10, 132.87, 131.60, 130.97, 130.90, 121.62, 120.34, 115.96, 115.79. ESI-HRMS calcd for C_15_H_12_ClN_4_O^+^ ([M + H]^+^): 299.0694; found: 299.0703.

*N-(4-(1H-1,2,4-triazol-1-yl)phenyl)-3-chlorobenzamide* (**6c**): White solid, Yield 62%, Mp 209–210 °C. ^1^H NMR (500 MHz, DMSO*_d_*_6_) δ: 10.55 (s, 1H, NH), 9.25 (s, 1H, Triazole-H), 8.23 (s, 1H, Triazole-H), 8.05–7.60 (m, 8H, Ar-H). ^13^C NMR (125 MHz, DMSO*_d_*_6_) δ: 164.69, 152.75, 142.51, 138.90, 137.13, 133.75, 133.03, 132.05, 130.95, 127.93, 127.03, 121.70, 120.36. ESI-HRMS calcd for C_15_H_12_ClN_4_O^+^ ([M + H]^+^): 299.0694; found: 299.0705.

*N-(4-(1H-1,2,4-triazol-1-yl)phenyl)-4-chlorobenzamide* (**6d**): White solid, Yield 57%, Mp 259–261 °C. ^1^H NMR (500 MHz, DMSO*_d_*_6_) δ: 10.52 (s, 1H, NH), 9.24 (s, 1H, Triazole-H), 8.24 (s, 1H, Triazole-H), 7.92–7.35 (m, 8H, Ar-H). ^13^C NMR (125 MHz, DMSO*_d_*_6_) δ: 165.03, 152.73, 142.48, 138.99, 137.07, 133.84, 132.95, 130.15, 129.00, 121.65, 120.35. ESI-HRMS calcd for C_15_H_12_ClN_4_O^+^ ([M + H]^+^): 299.0694; found: 299.0702.

*N-(4-(1H-1,2,4-triazol-1-yl)phenyl)-2-fluorobenzamide* (**6e**): White solid, Yield 53%, Mp 195–196 °C. ^1^H NMR (500 MHz, DMSO*_d_*_6_) δ: 10.65 (s, 1H, NH), 9.24 (s, 1H, Triazole-H), 8.24 (s, 1H, Triazole-H), 7.92–7.35 (m, 8H, Ar-H). ^13^C NMR (125 MHz, DMSO*_d_*_6_) δ: 163.46, 160.35, 158.37, 152.71, 142.47, 138.80, 133.25, 132.96, 130.39, 125.13, 121.10, 120.51, 116.77, 116.60. ESI-HRMS calcd for C_15_H_12_FN_4_O^+^ ([M + H]^+^): 283.0990; found: 283.0999.

*N-(4-(1H-1,2,4-triazol-1-yl)phenyl)-3-fluorobenzamide* (**6f**): White solid, Yield 48%, Mp 223–224 °C. ^1^H NMR (500 MHz, DMSO*_d_*_6_) δ: 10.52 (s, 1H, NH), 9.25 (s, 1H, Triazole-H), 8.23 (s, 1H, Triazole-H), 7.98–7.47 (m, 8H, Ar-H). ^13^C NMR (125 MHz, DMSO*_d_*_6_) δ: 164.74, 163.38, 161.44, 152.74, 142.50, 138.90, 137.42, 133.01, 131.16, 124.42, 124.40, 121.69, 120.35, 119.04, 114.92. ESI-HRMS calcd for C_15_H_12_FN_4_O^+^ ([M + H]^+^): 283.0990; found: 283.0998.

*N-(4-(1H-1,2,4-triazol-1-yl)phenyl)-4-fluorobenzamide* (**6g**): White solid, Yield 41%, Mp 247–248 °C. ^1^H NMR (500 MHz, DMSO*_d_*_6_) δ: 10.73 (s, 1H, NH), 9.24 (s, 1H, Triazole-H), 8.23 (s, 1H, Triazole-H), 7.91–7.48 (m, 8H, Ar-H). ^13^C NMR (125 MHz, DMSO*_d_*_6_) δ: 165.53, 152.74, 142.50, 138.89, 137.19, 132.97, 131.73, 130.42, 130.18, 129.44, 127.78, 120.86, 120.52.ESI-HRMS calcd for C_15_H_12_FN_4_O^+^ ([M + H]^+^): 283.0990; found: 283.1000.

*N-(4-(1H-1,2,4-triazol-1-yl)phenyl)-2-(trifluoromethyl)benzamide* (**6h**): White solid, Yield 56%, Mp 219–220 °C. ^1^H NMR (500 MHz, DMSO*_d_*_6_) δ: 10.79 (s, 1H, NH), 9.24 (s, 1H, Triazole-H), 8.23 (s, 1H, Triazole-H), 7.88–7.74 (m, 8H, Ar-H). ^13^C NMR (125 MHz, DMSO*_d_*_6_) δ: 166.18, 152.75, 142.53, 138.87, 136.40, 133.15, 133.03, 130.68, 129.02, 126.88, 125.34, 123.16, 120.92, 120.57. ESI-HRMS calcd for C_16_H_12_F_3_N_4_O^+^ ([M + H]^+^): 333.0958; found: 333.0967.

*N-(4-(1H-1,2,4-triazol-1-yl)phenyl)-3-(trifluoromethyl)benzamide* (**6i**): White solid, Yield 66%, Mp 190–191 °C. ^1^H NMR (500 MHz, DMSO*_d_*_6_) δ: 10.67 (s, 1H, NH), 9.26 (s, 1H, Triazole-H), 8.24 (s, 1H, Triazole-H), 8.33–7.80 (m, 8H, Ar-H). ^13^C NMR (125 MHz, DMSO*_d_*_6_) δ:164.65, 152.75, 142.51, 138.80, 136.03, 133.11, 132.37, 130.27, 129.57, 128.79, 125.53, 124.75, 123.36, 121.83, 120.37. ESI-HRMS calcd for C_16_H_12_F_3_N_4_O^+^ ([M + H]^+^): 333.0958; found: 333.0966.

*N-(4-(1H-1,2,4-triazol-1-yl)phenyl)-4-(trifluoromethyl)benzamide* (**6j**): White solid, Yield 76%, Mp 293–294 °C. ^1^H NMR (500 MHz, DMSO*_d_*_6_) δ: 10.68(s, 1H, NH), 9.26 (s, 1H, Triazole-H), 8.24 (s, 1H, Triazole-H), 8.19–7.87 (m, 8H, Ar-H). ^13^C NMR (125 MHz, DMSO*_d_*_6_) δ: 164.99, 152.75, 142.51, 138.97, 138.82, 133.10, 131.85, 129.12, 125.93, 121.72, 120.37. ESI-HRMS calcd for C_16_H_12_F_3_N_4_O^+^ ([M + H]^+^): 333.0958; found: 333.0962.

*N-(4-(1H-1,2,4-triazol-1-yl)phenyl)-2-methylbenzamide* (**6k**): White solid, Yield 58%, Mp 197–198 °C. ^1^H NMR (500 MHz, DMSO*_d_*_6_) δ: 10.51 (s, 1H, NH), 9.23 (s, 1H, Triazole-H), 8.22 (s, 1H, Triazole-H), 7.94–7.32 (m, 8H, Ar-H), 2.41 (s, 3H, CH_3_). ^13^C NMR (125 MHz, DMSO*_d_*_6_) δ: 168.45, 152.70, 142.46, 139.30, 137.41, 135.78, 132.73, 131.05, 130.26, 127.72, 126.15, 120.88, 120.43, 19.78. ESI-HRMS calcd for C_16_H_15_N_4_O^+^ ([M + H]^+^): 279.1240; found: 279.1248.

*N-(4-(1H-1,2,4-triazol-1-yl)phenyl)-3-methylbenzamide* (**6l**): White solid, Yield 55%, Mp 183–184 °C. ^1^H NMR (500 MHz, DMSO*_d_*_6_) δ: 10.42 (s, 1H, NH), 9.24 (s, 1H, Triazole-H), 8.23 (s, 1H, Triazole-H), 7.98–7.43 (m, 8H, Ar-H), 2.42 (s, 3H, CH_3_). ^13^C NMR (125 MHz, DMSO*_d_*_6_) δ: 166.27, 152.71, 142.46, 139.26, 138.24, 135.18, 132.78, 128.82, 128.63, 125.34, 121.53, 120.33, 21.44. ESI-HRMS calcd for C_16_H_15_N_4_O^+^ ([M + H]^+^): 279.1240; found: 279.1251.

*N-(4-(1H-1,2,4-triazol-1-yl)phenyl)-4-methylbenzamide* (**6m**): White solid, Yield 63%, Mp 255–256 °C. ^1^H NMR (500 MHz, DMSO*_d_*_6_) δ: 10.36 (s, 1H, NH), 9.23 (s, 1H, Triazole-H), 8.22 (s, 1H, Triazole-H), 7.98–7.35 (m, 8H, Ar-H), 2.40 (s, 3H, CH_3_). ^13^C NMR (125 MHz, DMSO*_d_*_6_) δ: 165.96, 152.70, 142.45, 142.26, 139.30, 132.74, 132.28, 129.43, 128.21, 121.55, 120.32, 21.49. ESI-HRMS calcd for C_16_H_15_N_4_O^+^ ([M + H]^+^): 279.1240; found: 279.1250.

*N-(4-(1H-1,2,4-triazol-1-yl)phenyl)propionamide* (**9a**): White solid, Yield 65%, Mp 220–221 °C. ^1^H NMR (500 MHz, DMSO*_d_*_6_) δ: 10.08 (s, 1H, NH), 9.20 (s, 1H, Triazole-H), 8.20 (s, 1H, Triazole-H), 7.77 (s, 4H, Ar-H), 2.35 (q, 2H, *J* = 8.33 Hz, CH_2_), 1.10 (t, 3H, *J* = 7.5 Hz, CH_3_). ^13^C NMR (125 MHz, DMSO*_d_*_6_) δ: 172.66, 152.64, 142.37, 139.42, 132.22, 120.49, 120.18, 29.99, 10.03. ESI-HRMS calcd for C_11_H_13_N_4_O^+^ ([M + H]^+^): 217.1084; found: 217.1090.

*N-(4-(1H-1,2,4-triazol-1-yl)phenyl)butyramide* (**9b**): White solid, Yield 66%, Mp 152–153 °C. ^1^H NMR (500 MHz, DMSO*_d_*_6_) δ: 10.15 (s, 1H, NH), 9.19 (s, 1H, Triazole-H), 8.22 (s, 1H, Triazole-H), 7.78 (s, 4H, Ar-H), 2.33 (t, 2H, *J* = 7.5 Hz, CH_2_), 1.66–1.62 (m, 2H, CH_2_), 0.94 (t, 3H, *J* = 7.5 Hz, CH_3_). ^13^C NMR (125 MHz, DMSO*_d_*_6_) δ: 172.13, 152.58, 142.33, 139.27, 132.27, 120.52, 120.38, 38.75, 18.98, 14.02. ESI-HRMS calcd for C_12_H_15_N_4_O^+^ ([M + H]^+^): 231.1240; found: 231.1250.

*N-(4-(1H-1,2,4-triazol-1-yl)phenyl)pentanamide* (**9c**): White solid, Yield 71%, Mp 120–121 °C. ^1^H NMR (500 MHz, DMSO*_d_*_6_) δ: 10.09 (s, 1H, NH), 9.19 (s, 1H, Triazole-H), 8.20 (s, 1H, Triazole-H), 7.77 (s, 4H, Ar-H), 2.34 (t, 2H, *J* = 7.5 Hz, CH_2_), 1.61–1.33 (m, 4H, CH_2_), 0.91 (t, 3H, *J* = 7.5 Hz, CH_3_). ^13^C NMR (125 MHz, DMSO*_d_*_6_) δ: 171.98, 152.65, 142.39, 139.40, 132.26, 120.48, 120.22, 36.60, 27.64, 22.29, 14.20. ESI-HRMS calcd for C_13_H_17_N_4_O^+^ ([M + H]^+^): 245.1397; found: 245.1406.

*N-(4-(1H-1,2,4-triazol-1-yl)phenyl)hexanamide* (**9d**): White solid, Yield 64%, Mp 153–154 °C. ^1^H NMR (500 MHz, DMSO*_d_*_6_) δ: 10.08 (s, 1H, NH), 9.19 (s, 1H, Triazole-H), 8.20 (s, 1H, Triazole-H), 7.77 (s, 4H, Ar-H), 2.34 (t, 2H, *J* = 7.5 Hz, CH_2_), 1.63–1.30 (m, 6H, CH_2_), 0.88 (t, 3H, *J* = 7.5 Hz, CH_3_). ^13^C NMR (125 MHz, DMSO*_d_*_6_) δ: 171.97, 152.64, 142.37, 139.38, 132.25, 120.47, 120.21, 36.85, 31.36, 25.19, 22.37, 14.33. ESI-HRMS calcd for C_14_H_19_N_4_O^+^ ([M + H]^+^): 259.1553; found: 259.1563.

*N-(4-(1H-1,2,4-triazol-1-yl)phenyl)heptanamide* (**9e**): White solid, Yield 58%, Mp 151–152 °C. ^1^H NMR (500 MHz, DMSO*_d_*_6_) δ: 10.16 (s, 1H, NH), 9.18 (s, 1H, Triazole-H), 8.22 (s, 1H, Triazole-H), 7.77 (s, 4H, Ar-H), 2.34 (t, 2H, *J* = 7.5 Hz, CH_2_), 1.62–1.29 (m, 8H, CH_2_), 0.87 (t, 3H, *J* = 7.5 Hz, CH_3_). ^13^C NMR (125 MHz, DMSO*_d_*_6_) δ: 172.31, 152.56, 142.30, 139.27, 132.26, 120.52, 120.39, 36.84, 31.42, 28.71, 25.46, 22.40, 14.33. ESI-HRMS calcd for C_15_H_21_N_4_O^+^ ([M + H]^+^): 273.1710; found: 273.1719.

*N-(4-(1H-1,2,4-triazol-1-yl)phenyl)octanamide* (**9f**): White solid, Yield 55%, Mp 148–149 °C. ^1^H NMR (500 MHz, DMSO*_d_*_6_) δ: 10.07 (s, 1H, NH), 9.19 (s, 1H, Triazole-H), 8.20 (s, 1H, Triazole-H), 7.77 (s, 4H, Ar-H), 2.33 (t, 2H, *J* = 7.5 Hz, CH_2_), 1.62–1.26 (m, 10H, CH_2_), 0.87 (t, 3H, *J* = 7.5 Hz, CH_3_). ^13^C NMR (125 MHz, DMSO*_d_*_6_) δ: 171.98, 152.65, 142.38, 139.40, 132.26, 120.48, 120.22, 36.88, 31.63, 29.10, 28.93, 25.51, 22.53, 14.39. ESI-HRMS calcd for C_16_H_23_N_4_O^+^ ([M + H]^+^): 287.1866; found: 287.1874.

*N-(4-(1H-1,2,4-triazol-1-yl)phenyl)nonanamide* (**9g**): White solid, Yield 49%, Mp 146–147 °C. ^1^H NMR (500 MHz, DMSO*_d_*_6_) δ: 10.08 (s, 1H, NH), 9.19 (s, 1H, Triazole-H), 8.20 (s, 1H, Triazole-H), 7.77 (s, 4H, Ar-H), 2.33 (t, 2H, *J* = 7.5 Hz, CH_2_), 1.62–1.26 (m, 12H, CH_2_), 0.86 (t, 3H, *J* = 7.5 Hz, CH_3_). ^13^C NMR (125 MHz, DMSO*_d_*_6_) δ: 171.98, 152.65, 142.38, 139.40, 132.26, 120.47, 120.21, 36.89, 31.72, 29.23, 29.14, 29.06, 25.50, 22.54, 14.41. ESI-HRMS calcd for C_17_H_25_N_4_O^+^ ([M + H]^+^): 301.2023; found: 301.2032.

### 3.2. Pharmacology

#### 3.2.1. Animals and Treatment

The experimental animals used were Kunming mice (18–22 g) and Wistar rats (280–300 g). The experimental conditions included temperature of 20–25 °C and humidity of 45–65%, and the experimental animals were allowed to drink freely 12 h before the experiment, but no food was allowed. The target compounds were dissolved in 0.9% NaCl or DMSO and administered subcutaneously or intraperitoneally.

#### 3.2.2. Biological Evaluation

The pharmacological part includes MES, *sc*PTZ, ROT, radioreceptor binding, EPM, and GABA content determination experiments. The specific experimental methods are shown in the Supplementary Materials.

The steps of the pharmacological experiments are as follows: Firstly, the anticonvulsant activity of the target compounds was determined using two experimental models, MES and *sc*PTZ. The neurotoxicity of the target compounds was determined using the ROT experimental model. All the above experiments were performed according to the methods described in the National Institutes of Health (NIH) Antiepileptic Drug Development Program (ADD) [26,27,28]. Secondly, based on the results of the above anticonvulsant activity and neurotoxicity tests, potential compounds with high activity and low toxicity were screened and quantitatively evaluated for their ED_50_ and TD_50_, and the affinity of the potential compounds to the GABA_A_ receptor was determined using the in vitro method of radioreceptor binding assays [29]. Finally, the preferred compounds were selected based on the results of in vivo quantitative evaluation assays and in vitro affinity assays. The possible mechanisms of the action of the preferred compounds were further investigated using GABA content determination [30,31] and EPM experiments [32,33]. The data obtained from the experiments were analyzed and processed and plotted using GraphPad Prism 8 software.

#### 3.2.3. In Silico Study

This part includes the prediction of physicochemical and pharmacokinetic properties of all target compounds and the simulation of molecular docking experiments of the optimal compounds. The prediction software used is DS 2021. Physicochemical and pharmacokinetic properties predictions include MW, HD, HA, RotB, ClogP, ABS, and BBB permeability of target compounds.

The representative compound **6l** was selected as the ligand and the GABA_A_ receptor as the receptor for the molecular docking studies. The 3D crystal structure of the GABA_A_ receptor (PDB Code: 6HUP) was obtained from the RCSB Protein Data Bank (https://www.rcsb.org), and the docking site was selected as the BZ binding site of the GABA_A_ receptor. The specific docking method is described in the Supplementary Material. Molecular dynamics simulations were performed on the molecular docking results to further validate the stability of potential ligand–receptor interactions. The specific molecular dynamics simulation methods are described in the Supplementary Materials.

## 4. Conclusions

In this paper, twenty target compounds with triazole structural fragments were designed and synthesized using 4-bromoaniline as raw material, and all the target compounds structure were confirmed by the ^1^H NMR, ^13^C NMR, and HRMS methods. All the target compounds were screened for anticonvulsant activity and neurotoxicity using MES, *sc*PTZ, and ROT in vivo animal models, the anticonvulsant activity and neurotoxicity of the potential compounds were quantitatively evaluated, and the preferred compounds, **6f** and **6l**, were finally obtained. The ED_50_ values of compound **6f** in the MES and *sc*PTZ models were 13.1 and 19.7 mg/kg, respectively, and the PI values were 36.3 and 24.2, respectively. The ED_50_ values of compound **6l** in the MES and *sc*PTZ models were 9.1 and 19.0 mg/kg, respectively, and the PI values were 45.9 and 22.1, respectively. Both compounds were higher than the control group in safety. The affinity test showed that compound **6f** has strong affinity for the GABA_A_ receptor, with an IC_50_ of 0.14 μM. The results of inhibitory neurotransmitter GABA content in brain tissue showed that compounds **6f** and **6l** significantly increase GABA content in the brain of rats. The EPM assay of compounds **6f** and **6l** possessed anxiolytic effects, and the effects were correlated with the BZ binding site of the GABA_A_ receptor. The molecular docking results showed that compound **6l** was able to generate significant force with amino acid residues of the BZ binding site for the GABA_A_ receptor. Molecular dynamics simulation results indicate that the complex formed by molecular docking is stable. In conclusion, this series of compounds has the potential to be used as anticonvulsant lead compounds containing the structural fragments of triazole.

## Figures and Tables

**Table 1:** Results of MES and *sc*PTZ tests for target compounds.

Compounds	MES ^a^ (mg/kg)		Compounds	*sc*PTZ ^a^ (mg/kg)	
	0.5 h	4 h		0.5 h	4 h
**6a**	300	300	**6a**	100	100
**6b**	300	100	**6b**	100	300
**6c**	100	300	**6c**	100	100
**6d**	300	300	**6d**	300	-
**6e**	100	100	**6e**	100	100
**6f**	30	30	**6f**	30	30
**6g**	30	30	**6g**	30	100
**6h**	100	300	**6h**	300	-
**6i**	30	30	**6i**	30	100
**6j**	100	300	**6j**	100	100
**6k**	30	100	**6k**	100	300
**6l**	30	30	**6l**	30	30
**6m**	300	300	**6m**	100	300
**9a**	100	300	**9a**	100	100
**9b**	30	100	**9b**	30	100
**9c**	30	30	**9c**	30	100
**9d**	30	30	**9d**	100	100
**9e**	100	300	**9e**	300	-
**9f**	100	300	**9f**	300	-
**9g**	300	300	**9g**	300	-
**I**	30	30	**I**	30	30

^a^ Three experimental animals were used for each dose, at least one animal was effective at this dose; (-) Not effective at 30, 100 and 300 mg/kg.

**Table 2:** Results of ROT test for target compounds.

Compounds	NT ^a^ (mg/kg)		Compounds	NT (mg/kg)	
	0.5 h	4 h		0.5 h	4 h
**6a**	>300	>300	**6l**	>300	>300
**6b**	>300	>300	**6m**	>300	>300
**6c**	>300	>300	**9a**	>300	>300
**6d**	>300	>300	**9b**	300	>300
**6e**	>300	>300	**9c**	300	>300
**6f**	>300	>300	**9d**	>300	>300
**6g**	>300	>300	**9e**	>300	>300
**6h**	>300	>300	**9f**	>300	>300
**6i**	>300	>300	**9g**	>300	>300
**6j**	>300	>300	**I**	>150	>150
**6k**	>300	>300	**-**	-	-

^a^ Three experimental animals were used at each dose, and neurotoxicity was observed in at least one animal at this dose.

**Table 3:** Quantitative evaluation data for potential compounds.

Compounds	ED_50_ ^a^ (MES)	ED_50_ (*sc*PTZ)	TD_50_ ^b^ (ROT)	PI ^c^	
				MES	*sc*PTZ
**6f**	13.1 (15.4–11.2)	19.7 (21.4–18.1)	476.7 (518.3–438.5)	36.3	24.2
**6g**	13.7 (16.1–11.7)	31.5 (34.3–29.0)	429.1 (466.4–394.7)	31.3	13.6
**6i**	21.9 (25.7–18.6)	22.8 (26.8–19.5)	381.4 (414.6–350.8)	17.4	16.7
**6l**	9.1 (10.7–7.8)	19.0 (22.3–16.2)	419.5 (456.1–385.9)	45.9	22.1
**9c**	17.3 (18.8–15.94)	35.5 (38.5–32.6)	260.0 (282.7–239.2)	15.0	7.3
**I** [14]	11.3 (12.3–10.4)	9.5 (10.4–8.8)	190.7 (207.3–175.4)	16.8	20.0
**Carbamazepine**	11.4 (12.4–10.5)	-	69.3 (75.4–63.8)	6.1	-
**Ethosuximide**	-	94.4 (86.8–102.6)	333.7 (362.8–306.9)	-	3.5

^a^ ED_50_: 50% effective dose, Unit: mg; ^b^ TD_50_: 50% toxic dose, Unit: mg; ^c^ PI: protective index (TD_50_/ED_50_); (-) not tested.

**Table 4:** Results of affinity experiments to test potential compounds.

Compounds	IC_50_ (μM) ^a^ (95% CI)
**6f**	0.14 (0.12–0.16)
**6g**	1.92 (1.52–2.41)
**6i**	0.35 (0.31–0.39)
**6l**	0.22 (0.19–0.25)
**9c**	1.21 (1.01–1.46)
**Diazepam**	0.034 (0.028–0.044)

^a^ The experiment was repeated three times.

**Table 5:** Physicochemical and pharmacokinetic predictions of the target compounds.

Compounds	Physicochemical					Pharmacokinetic	
	MW	ClogP	nHD	nHA	nRotB	ABS ^a^ (Level)	BBB ^b^ (Level)
**6** **a**	264.282	2.04	1	3	3	0	2
**6** **b**	298.727	1.96	1	3	3	0	2
**6** **c**	298.727	2.79	1	3	3	0	2
**6** **d**	298.727	2.79	1	3	3	0	2
**6** **e**	282.272	1.81	1	3	3	0	2
**6** **f**	282.272	2.22	1	3	3	0	2
**6** **g**	282.272	2.22	1	3	3	0	2
**6** **h**	332.28	1.80	1	3	4	0	2
**6** **i**	332.28	3.01	1	3	4	0	2
**6** **j**	332.28	3.01	1	3	4	0	2
**6k**	278.309	2.20	1	3	3	0	2
**6l**	278.309	2.54	1	3	3	0	2
**6m**	278.309	2.54	1	3	3	0	2
**9a**	216.239	1.08	1	3	3	0	3
**9b**	230.266	1.61	1	3	4	0	3
**9c**	244.292	2.14	1	3	5	0	2
**9d**	258.319	2.67	1	3	6	0	2
**9e**	272.346	3.20	1	3	7	0	2
**9f**	286.372	3.73	1	3	8	0	2
**9g**	300.399	4.26	1	3	9	0	2

^a^ Absorption-Level: 0 (very well absorbed), 1 (well absorbed), 2 (poorly absorbed), 3 (very poorly absorbed); ^b^ BBB-Level: 0 (very high penetration), 1 (high penetration), 2 (medium penetration), 3 (weak penetration).

## Data Availability

The data presented in this study are available upon request from the corresponding author.

## Supplementary Materials

The following supporting information can be downloaded at: https://www.mdpi.com/article/10.3390/molecules30163400/s1. Figure S1: The binding site of compound 6l with the receptor. Figure S2: Configuration of the solvated complex for the MD simulation. Figures S3–S22: ^1^H NMR, ^13^C NMR, and HRMS spectrum of compound **6a**–**m** and **9a**–**g**. Figures S23 and S24: HPLC spectrum of compounds **6f** and **6l**. Refs. [34,35,36,37,38,39,40,41,42,43] are cited in the Supplementary Materials.
