# Supplementary material for: Design, Synthesis, and Pharmacological Activity of the N-(4-(1H-1,2,4-Triazol-1-yl)phenyl)-substituted-amide Derivatives
Source: Molecules. 2025 Aug 18;30(16):3400. doi: 10.3390/molecules30163400 (PMC12388125; doi:10.3390/molecules30163400)
Supplement: Supplementary file 1 [file molecules-30-03400-s001.zip › molecules-3807108-supplementary.pdf]

Supporting Information

Lina Hu [1], Mengjiao Li [1], Zheng Liu [2], Hui Yan [1], Xuekun Wang [1,*] and Shiben Wang [1,*]

[1] State Key Laboratory of Macromolecular Drugs and Large-scale Preparation, School of Pharmaceutical Sciences and Food Engineering, Liaocheng University, Liaocheng 252059, China; 2019207801@stu.lcu.edu.cn (L.H.); 2210240102@stu.lcu.edu.cn (M.L.); yanhui@lcu.edu.cn (H.Y.)
[2] School of Medicine, Foshan University, Foshan 528000, China; liuzheng8709@fosu.edu.cn
* Correspondence: xuekunwang0610@126.com (X.W.); wangshiben110@163.com (S.W.);
Tel.: +86-0635-823-9087 (S.W.)

Pharmacology

MES test

Seizures were elicited using a 60 Hz alternating current with an intensity of 50 mA in mice. The current was applied via corneal electrodes for 0.2 s. Protection against the spread of MES-induced seizures was defined as the abolition of the hind leg, and the tonic maximal extension component of the seizure. Thirty minutes after administration of the compounds, their activities were evaluated in the MES test.

scPTZ test

This screen utilizes a dose of pentylenetetrazole (85 mg/kg) that produces clonic seizures lasting for a period of at least five seconds in 97% (CD_{97}) of animals tested. At the anticipated time of testing, the convulsant is administered subcutaneously. The test compound was administered intraperitoneally in mice, and the animals were observed over a 30 min period. Mice were tested at least two different time points (15 min, 30 min, 1h, or 4 h) following i.p administration of 100 and 300 mg/kg of test compound. Absence of clonic spasms indicated a compound's ability to counteract the effect of pentylenetetrazol on seizure threshold.

Rotarod test

The neurotoxicity of the compounds was measured in mice using the rotarod test. Mice were trained to stay on an accelerating rotarod (diameter, 3.2 cm) rotating at 10 rpm. Trained animals were injected with the test compounds (i.p.) and neurotoxicity was measured as the inability of the animal to maintain equilibrium on the rod for at least 1 min in each of the trials.

Binding assay

The radioreceptor binding assay was performed with synaptosomal membranes isolated from mouse cerebellum and was carried out as previously described with some modifications. Mice cerebellum was homogenized in 0.32 M sucrose and 10 mM HEPES buffer, pH 7.4, and centrifuged to collect the desired membrane-containing pellet. On the day of binding assay, the pellet was homogenized in assay buffer, centrifuged, and diluted in fresh assay buffer to a final protein concentration of 1 mg/ml. [^{3}H]flunitrazepam, brain homogenate, and flavonoid compounds (dissolved in DMSO and assayed at less than 0.2% final DMSO concentration) were placed into glass culture tubes for a final assay volume of 0.5 ml. The solutions were vortexed and allowed to incubate on ice for 50 min and filtered onto Whatman GF/B filter paper using a Brandel cell harvester M-24R. Filters were placed into vials and 2.5 ml scintillation fluid and shaken for 90 min. Samples were counted in a Packard Tri-Carb 2300 TR liquid scintillation counter. Specific binding was defined as the total amount bound (zero unlabeled ligand) minus the binding in the presence of 10 μM final concentration flurazepam. Data were analyzed with GraphPad Prism 8.0 software to determine IC_{50} value (one-site competition equation) and Hill slope (sigmoidal dose–response equation). Experiments were conducted in triplicate.

GABA estimation

Weigh about 0.05 g of tissue sample in a homogenizer, add 450 μL of extracting solution to homogenize, homogenize, transfer to an EP tube, mark the liquid level scale, and heat for 2 hours at 95°C. After heating, centrifuge at 8000 × g for 10 min and remove the supernatant for measurement.

① Standard tube: Take 30 μL of different concentrations of standards and add them to the corresponding 1.5 mL EP tube. Measurement tube: Take 30 μL of sample supernatant and add it to 1.5 mL EP tube. ② Add 50 μL of buffer solution and 40 μL of Chromogenic Agent A to each tube in step, mix well, and let stand at room temperature for 5 min. ③ Add 60 μL of Chromogenic Agent B to each tube in step, mix well, and heat at 95°C for 10 min, then cool at ice bath. ④ Add 200 μL of supplementary solution to each tube in step and mix well. ⑤ Take 200 μL into the corresponding wells of the enzyme plate, and measure the OD value of each well at 640 nm.

Elevated plus-maze (EPM)

The apparatus consisted of a black acrylic maze with two opposite open arms (50×10 cm) and two opposite closed arms (50×10×40 cm) disposed like a plus sign with a central square of 10 cm^2. Entire maze was elevated 50 cm above the floor. The test consisted in placing the rat in the central square of the maze facing one closed arm and allowing it to explore during 5 min. This procedure was performed in a quiet and dimly illuminated room. The scores analysed were the time spent in open and closed arms, and the number of entries to both arms. With these data were calculated the percentage of time spent in open arms which is widely used as an anxiety index (time spent in open arms relative to the time spent in open and closed arms). The number of entries in the closed arms was also assessed, as indicative of exploratory activity.

In silico study

Lipinski's "Rule of Five", which is commonly used in drug design and screening, takes into consideration the following aspects: molecular weight (MW ≤ 500), CLogP ≤ 5, number of hydrogen donors (nHD ≤ 5), number of acceptors (nHA ≤ 10), and the number of rotatable bonds (RotB ≤ 10). In fact, a compound that meets Lipinski's "Rule of Five" is more likely to be a suitable drug, even in the case of anticonvulsant drugs. We used Discovery Studio (DS) software to predict the physicochemical properties of the target compounds. As shown in Table 5, almost all target compounds met Lipinski's "Rule of Five".

In molecular docking studies, the BZs on the $GABA_A$ receptor (PDB Code: 6HUP, the binding site of compound **6l** on the $GABA_A$ receptor was consistent with that of diazepam. The binding site was determined by the position of the natural diazepam on the chain C\D from the X-ray diffraction structure, which is represented by a sphere with center coordinates at 117.693, 156.819, 109.343 and a radius of 10 Å. The molecular docking module (Lib dock) provided with the DS 2021 software was used for docking, and the simulation with the highest score was analyzed. The binding mode of compound **6l** is shown in the figure S1 below.

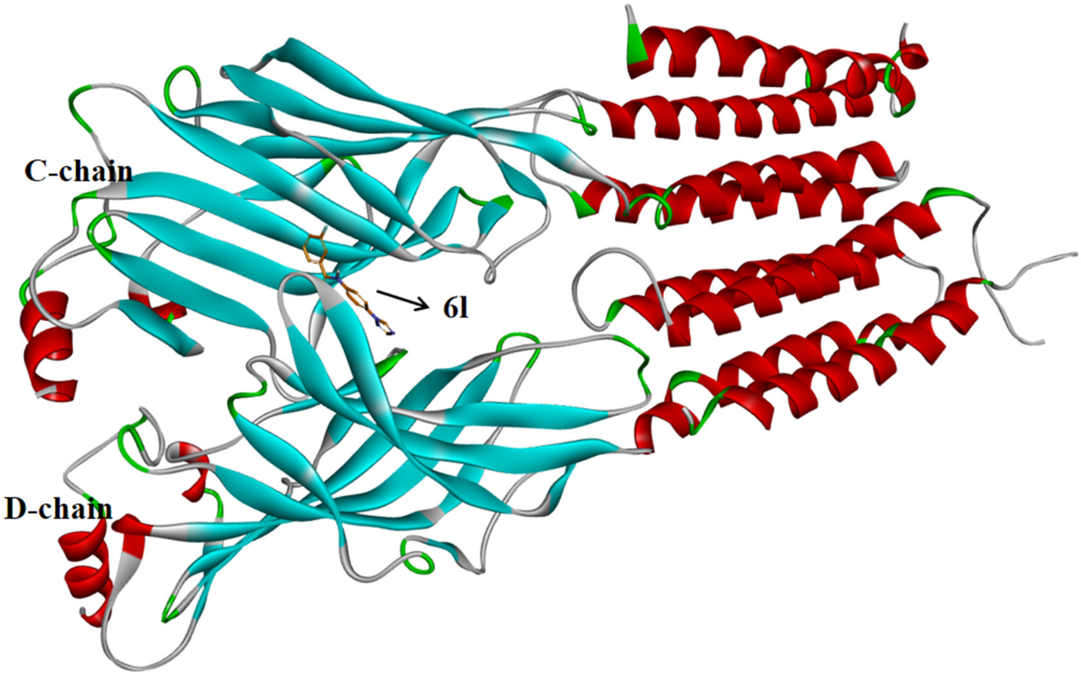

Figure S1. The binding site of compound **6l** with the receptor.

To confirm the stability of the complexes formed in the case of molecular docking, molecular dynamics simulations was performed for the ligand-receptor complexes. To set the simulation, complex was first placed in a rectangular box with a distance of 10 Å between the complex and box. Then the complex was solvated with water molecules and 24 Cl-ions were added into the box to keep system electrically neutral. The MD simulations were performed using the GROMACS 2023.3 software [34-37] with the CHARMm force field [38]. Water molecules were described by the TIP3P model [39]. The systems were first subjected to energy minimization calculations using the steepest descent method. Then, 20 ns MD simulations were performed under the NPT ensemble at 298 K and 1 atm. LINCS algorithm [40] was applied to constrain the bond lengths of other components. Periodic boundary conditions were applied in all three directions. The temperature and pressure was maintained using the V-rescale thermostat algorithm [41] and Berendsen barostat [42], respectively. The cut-off distance for the Lennard-Jones and electrostatic interactions was 1.2 nm. Particle mesh Ewald method [43] was used to calculate the long-range electrostatic interactions. The root mean square deviation (RMSD) with respect to the initial structures was computed during the MD simulations.

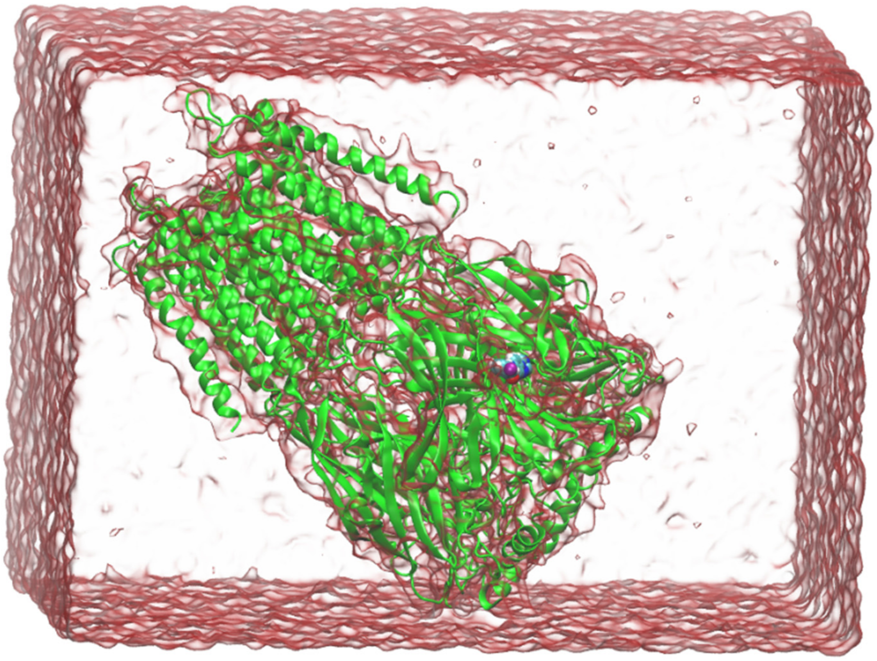

Figure S2-1. Configuration of the solvated complex for the MD simulation. The protein is shown in Cartoon model, and the solvent environment is simulated and displayed using red bubbles.

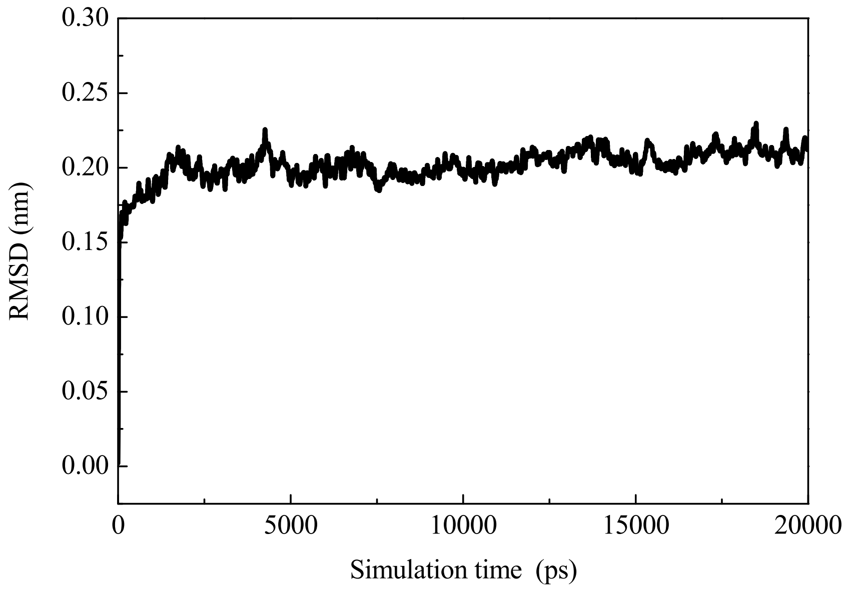

Figure S2-2. RMSD of the complex during the simulation.

¹H-NMR, ¹³C-NMR and HRMS spectrum of compound 6a-m and 9a-g.

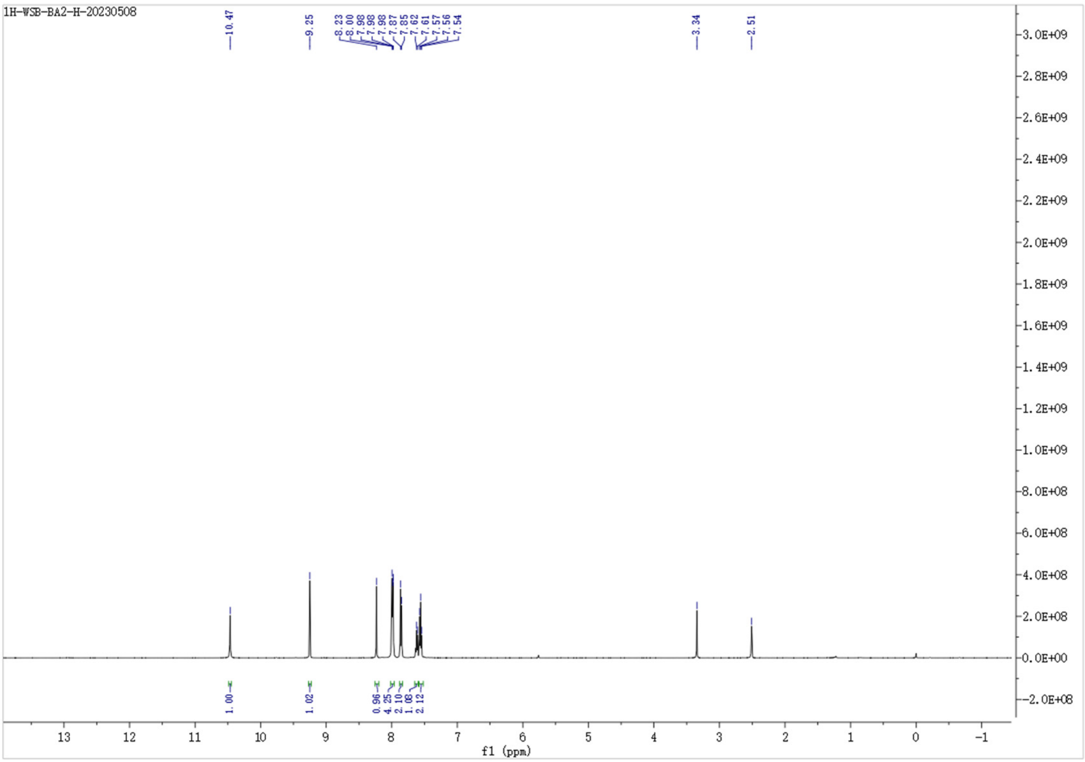

Figure S3-1. ¹H-NMR spectrum of compound 6a.

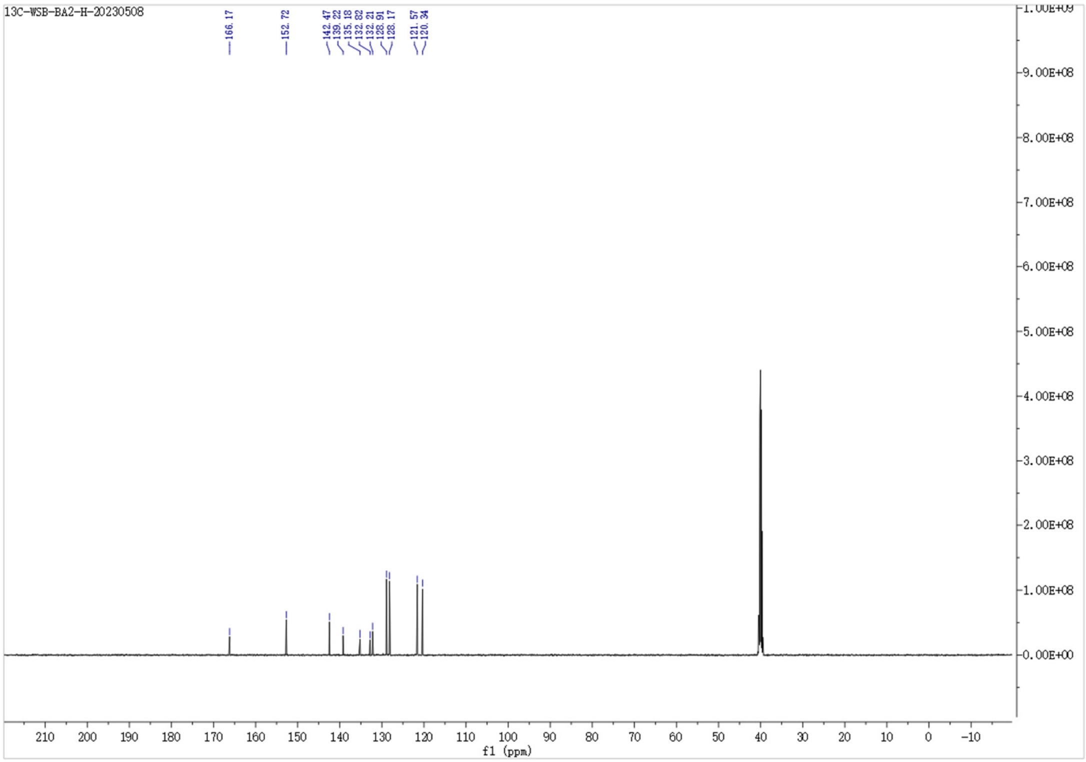

Figure S3-2. ¹³C-NMR spectrum of compound 6a.

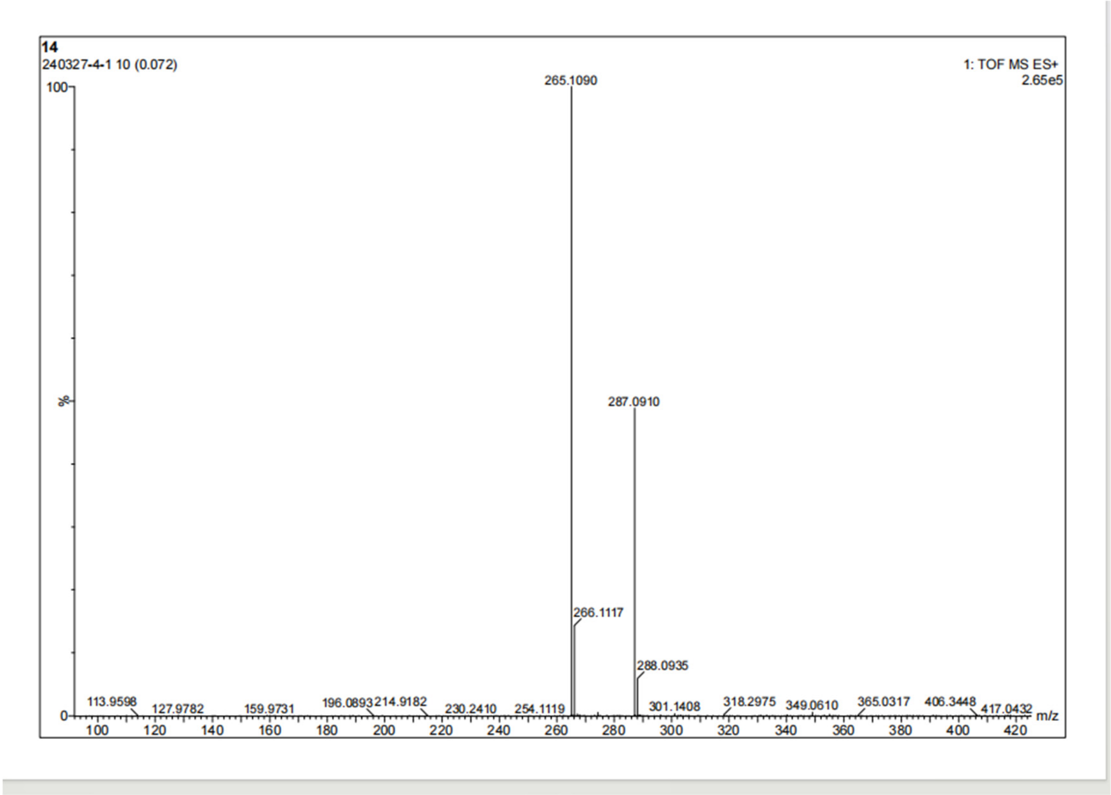

Figure S3-3. HRMS spectrum of compound 6a.

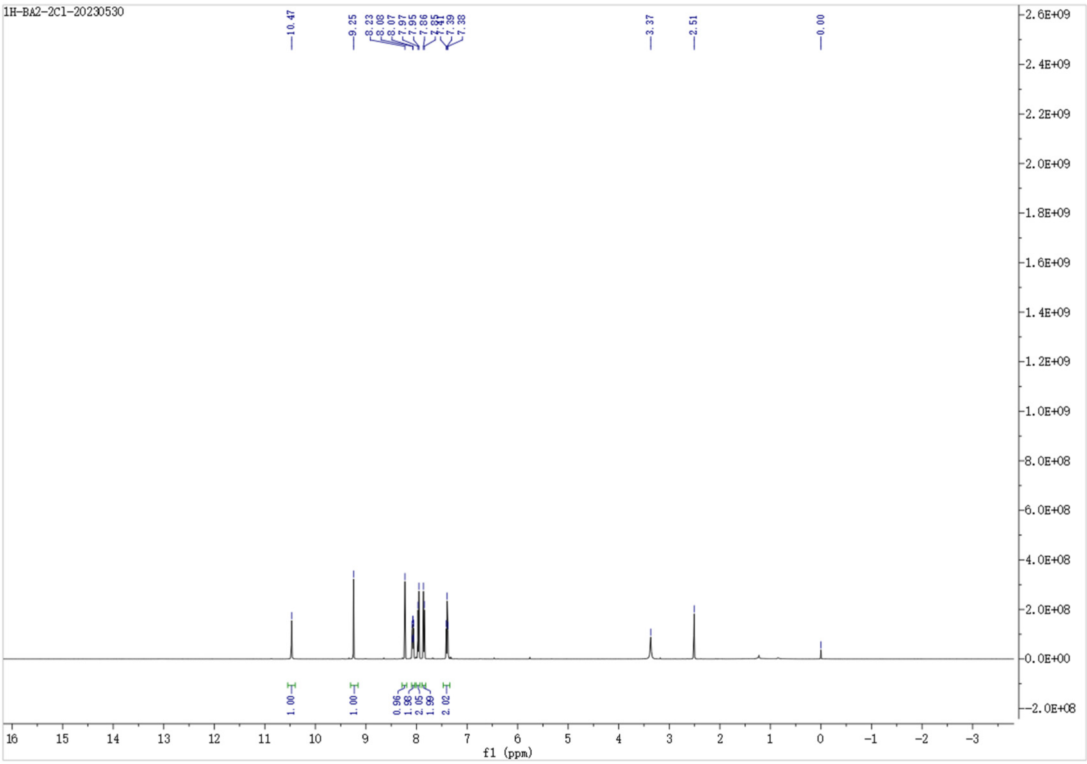

Figure S4-1. ^{1}H-NMR spectrum of compound 6b.

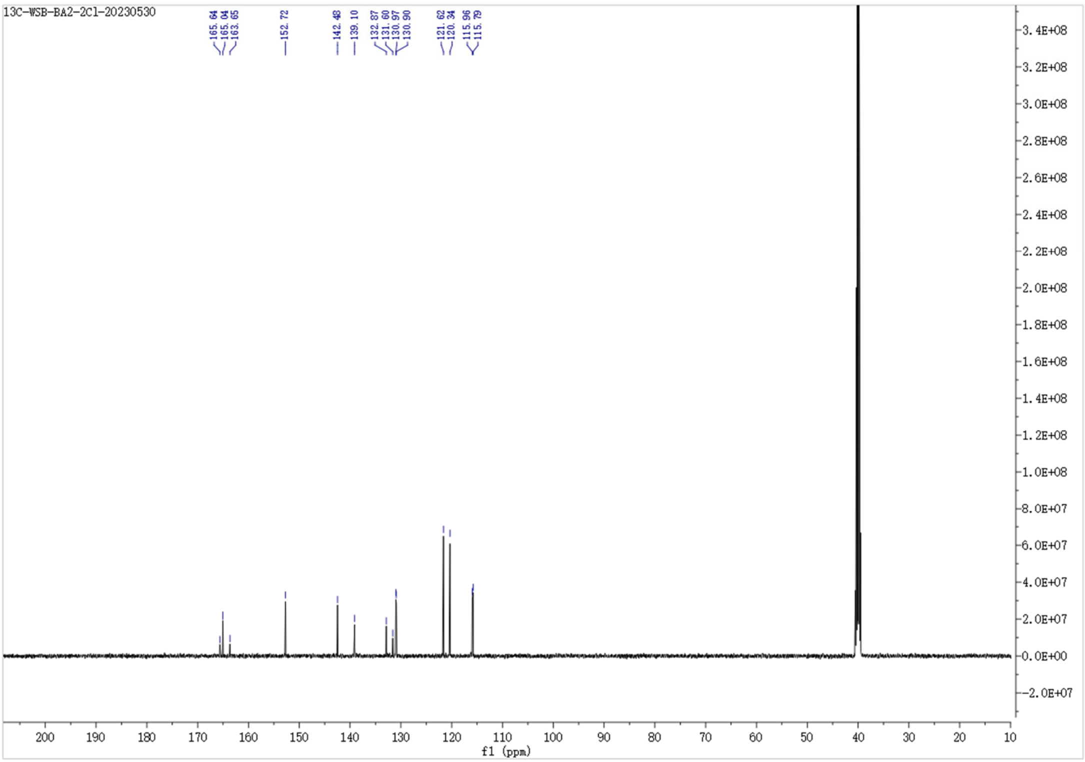

Figure S4-2. ^{13}**C-NMR spectrum of compound 6b.**

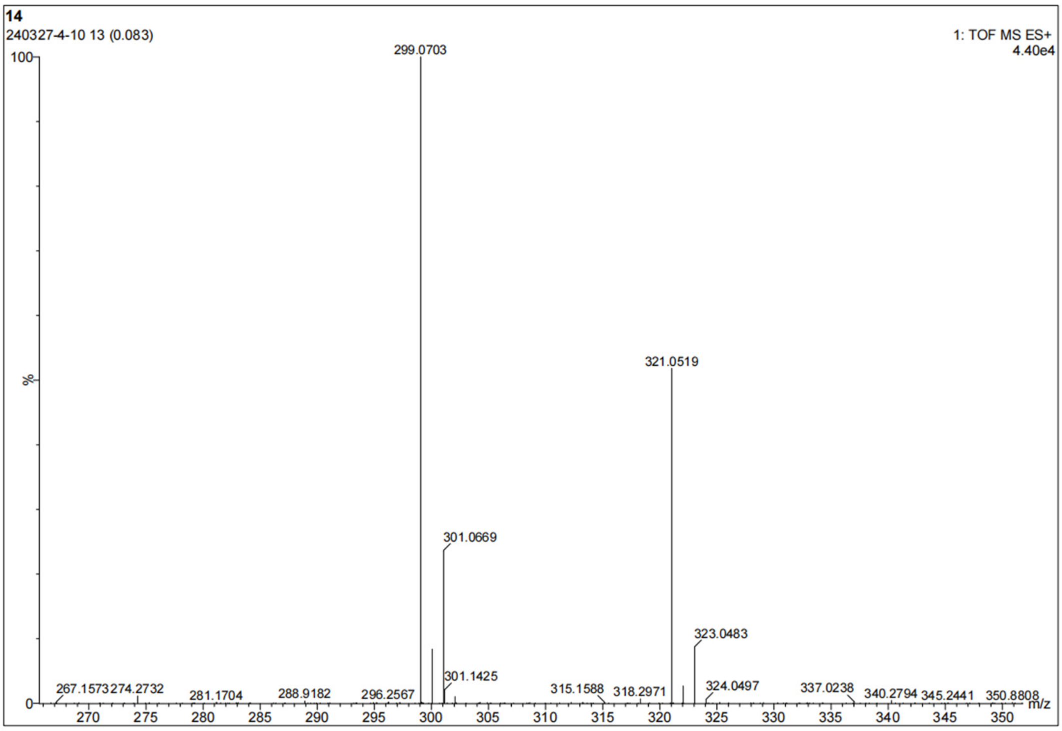

Figure S4-3. HRMS spectrum of compound 6b.

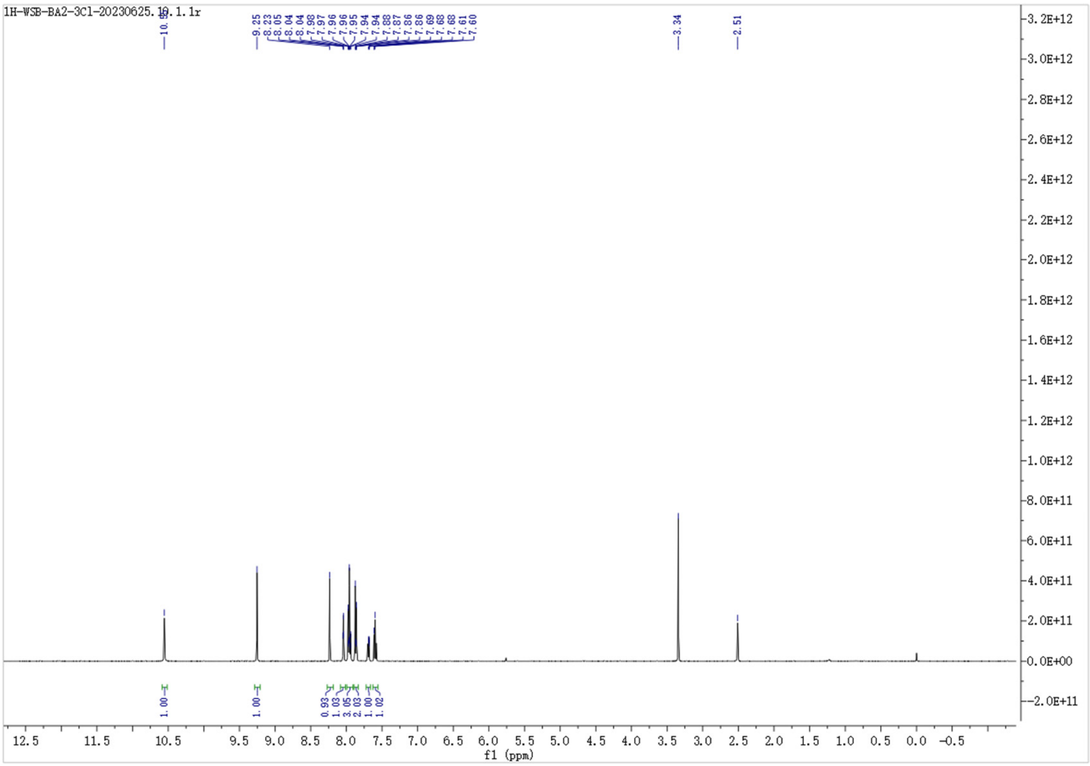

Figure S5-1. **^{1}H-NMR spectrum of compound 6c.**

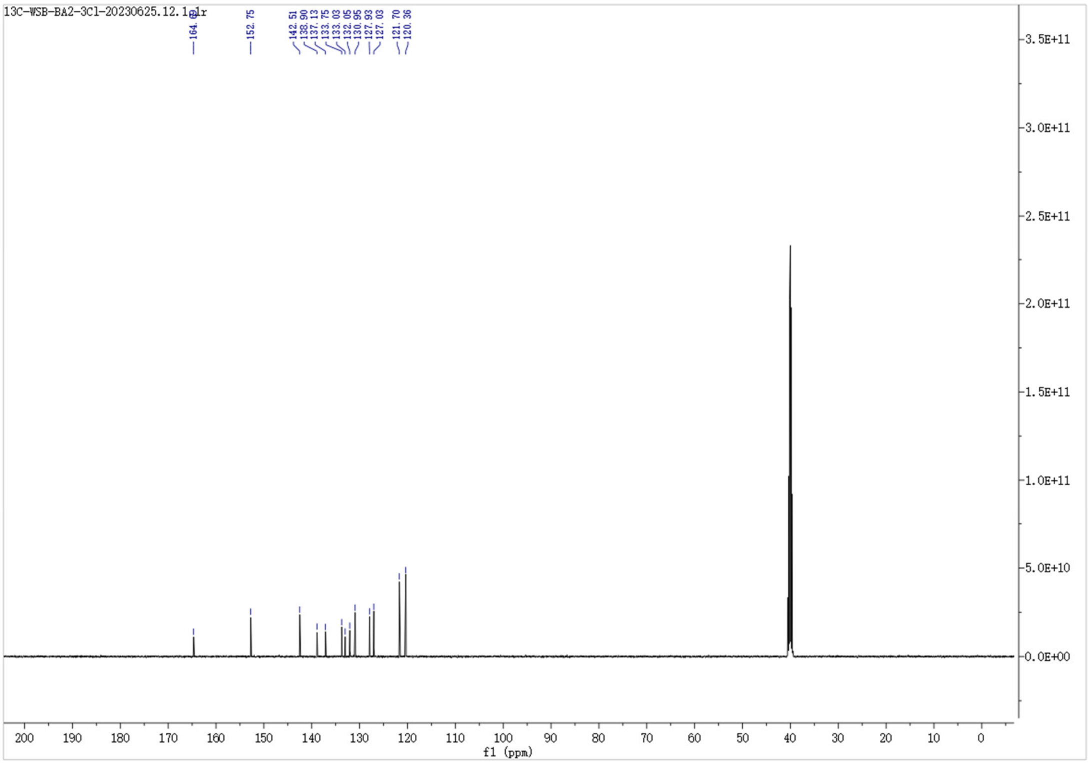

Figure S5-2. **^{13}C-NMR spectrum of compound 6c.**

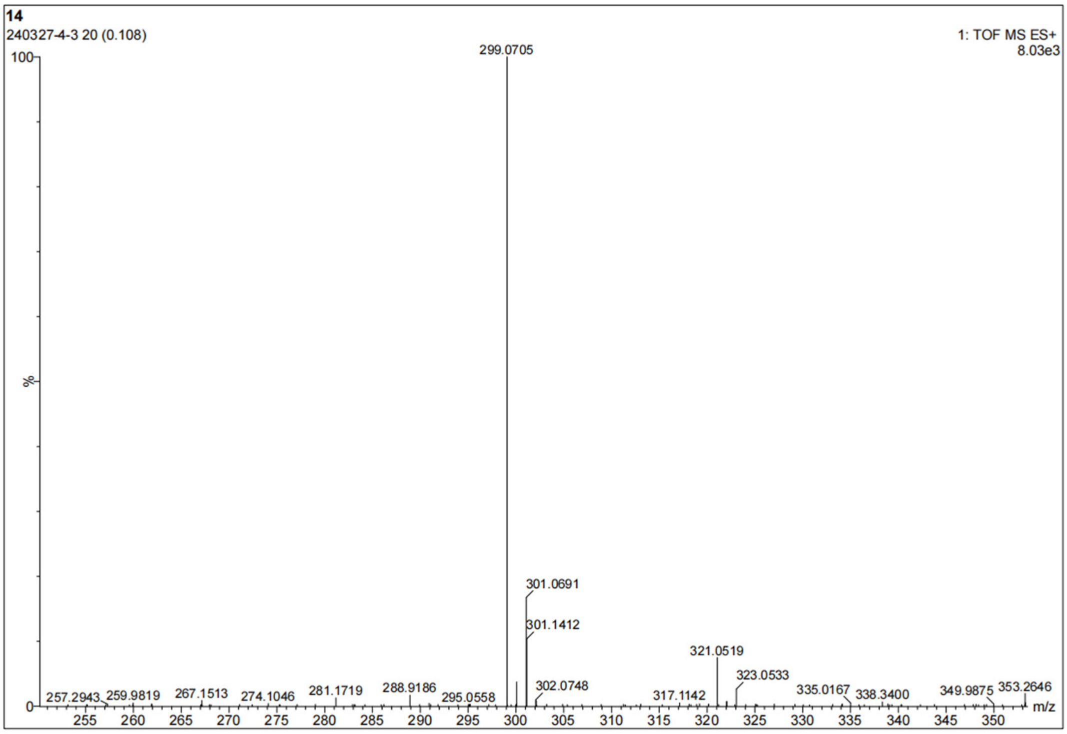

Figure S5-3. HRMS spectrum of compound 6c.

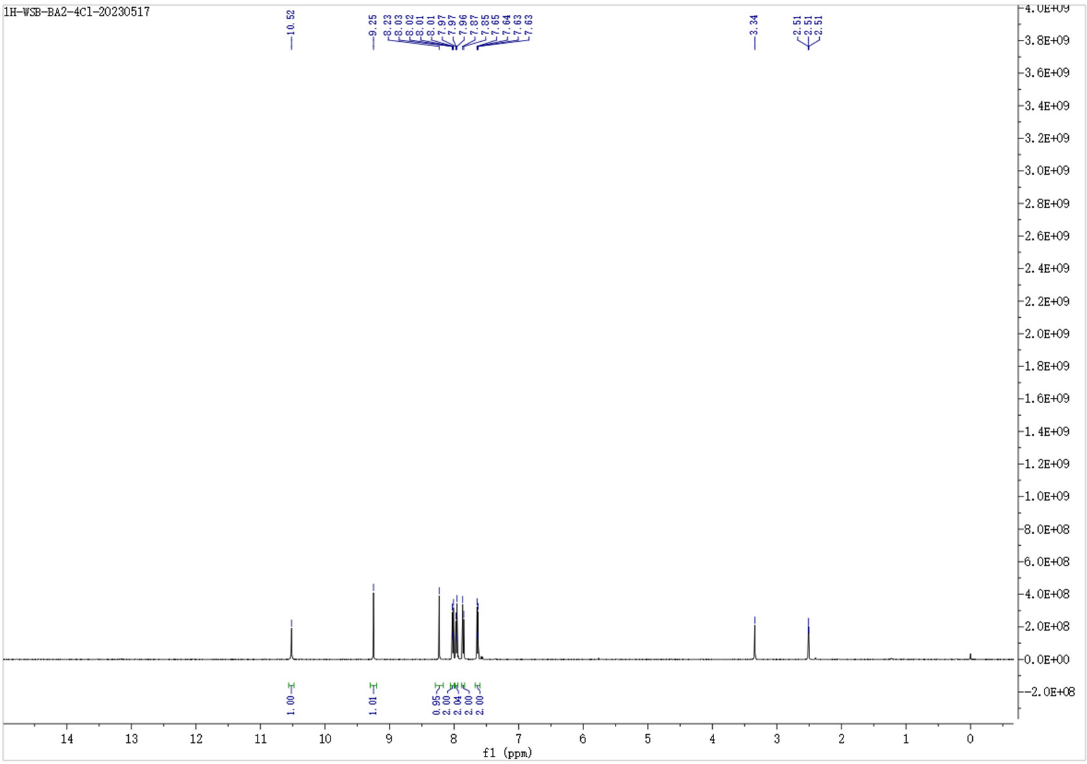

Figure S6-1. ^{1}H-NMR spectrum of compound 6d.

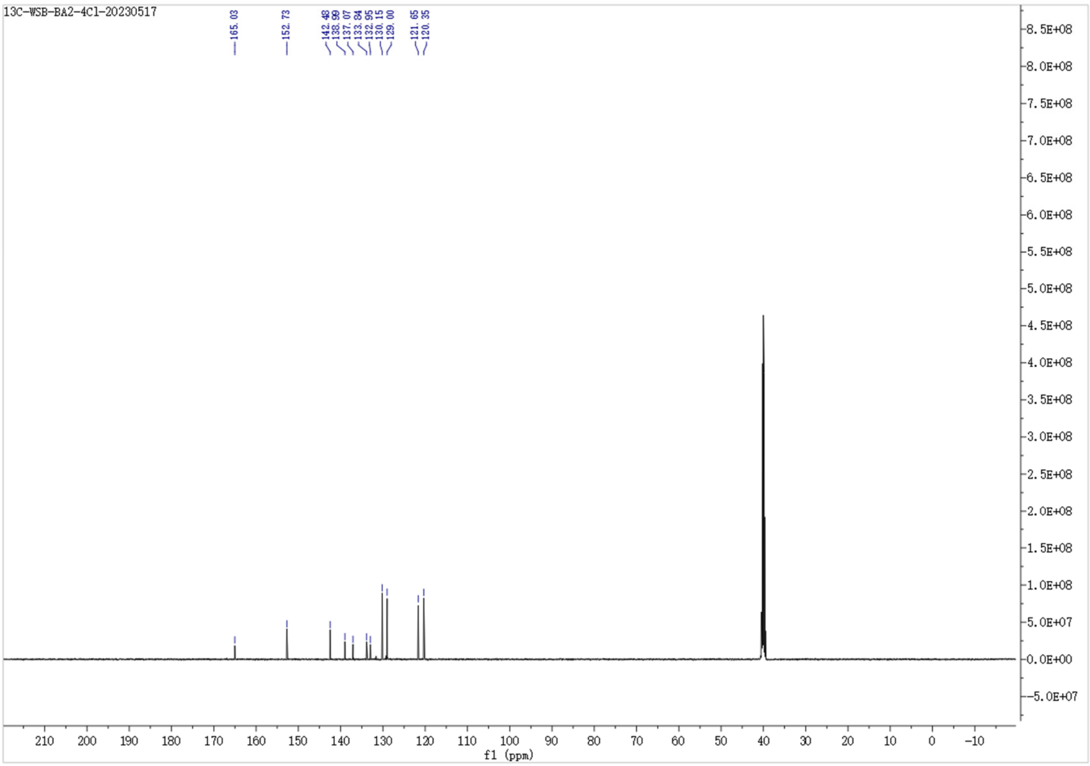

Figure S6-2. ^{13}**C-NMR spectrum of compound 6d.**

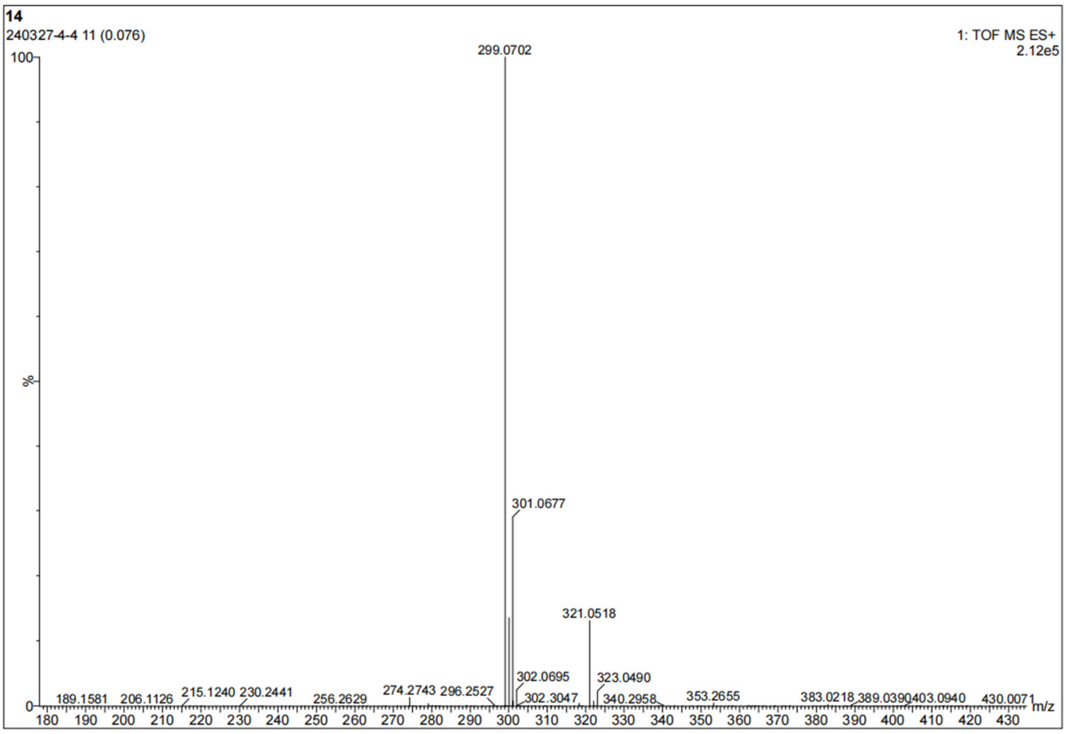

Figure S6-3. HRMS spectrum of compound 6d.

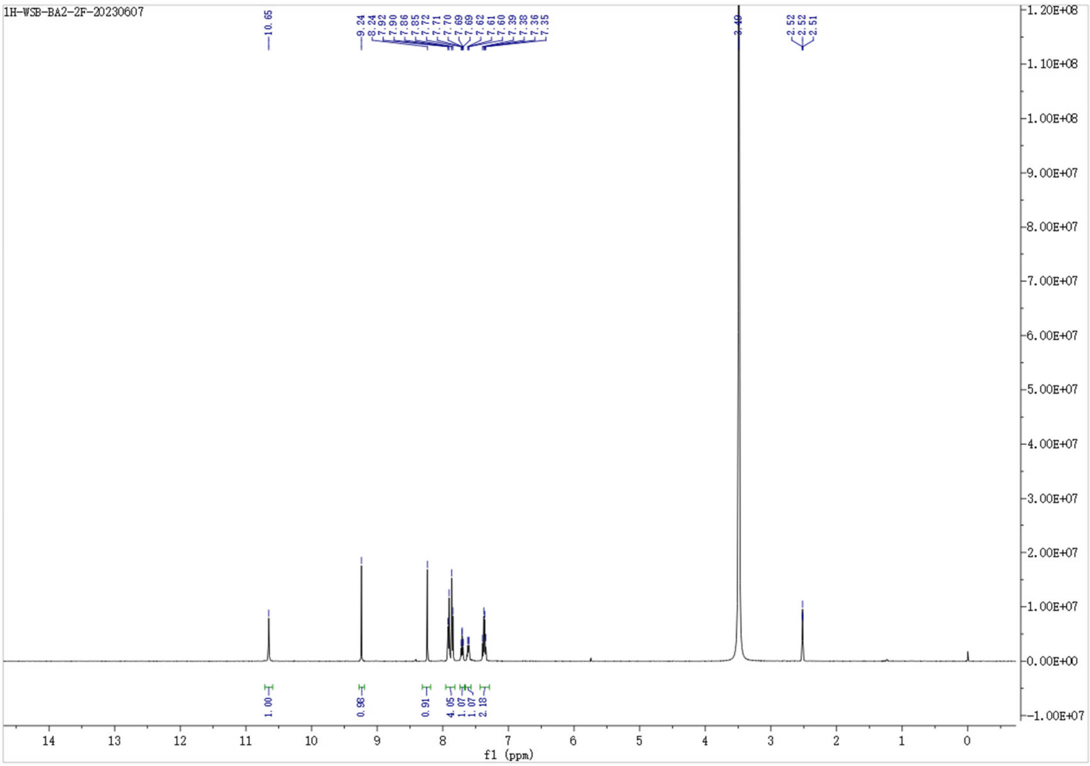

Figure S7-1. ^{1}H-NMR spectrum of compound 6e.

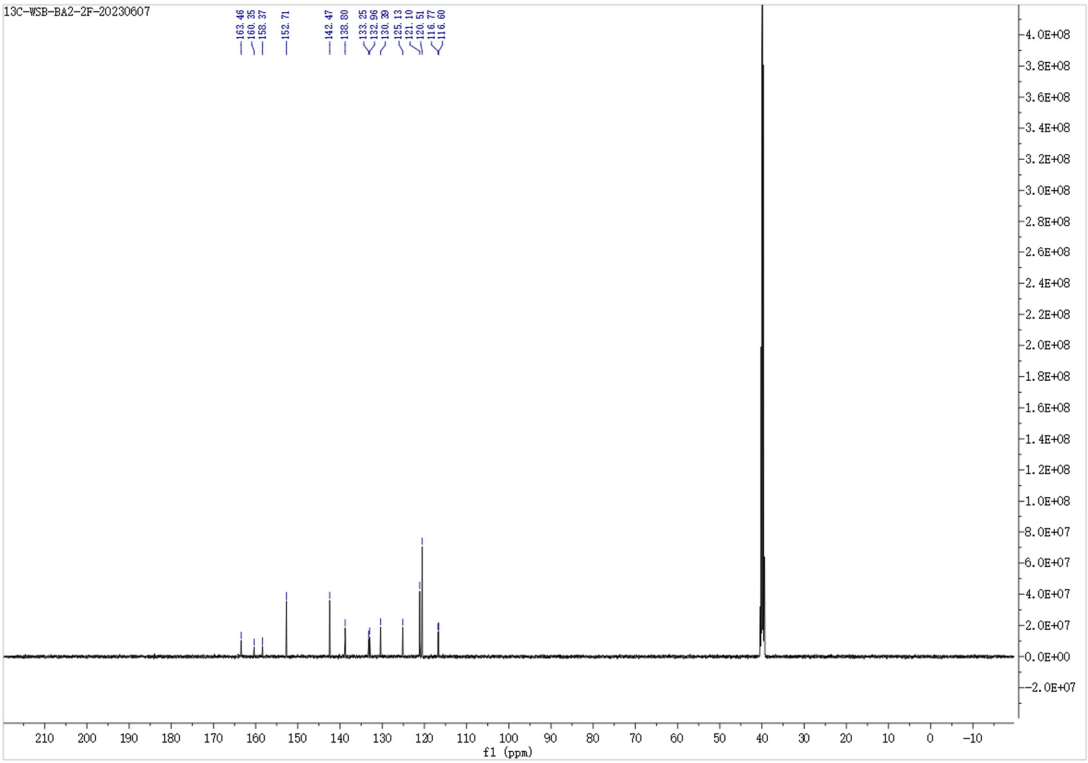

Figure S7-2. ^{13}C-NMR spectrum of compound 6e.

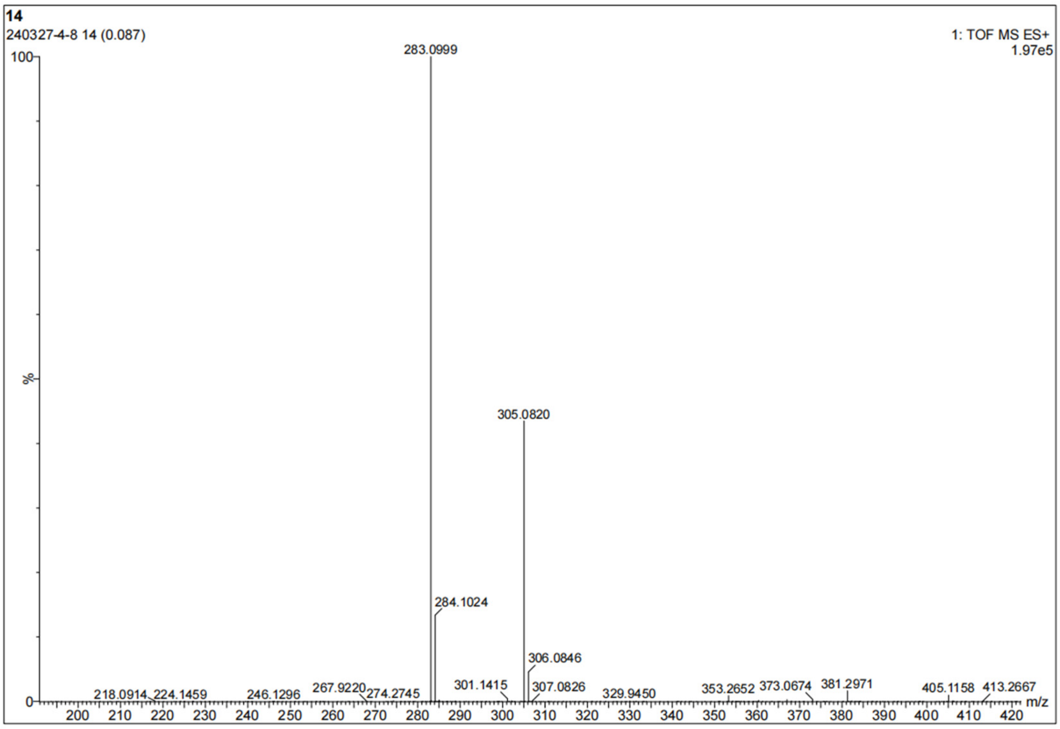

Figure S7-3. HRMS spectrum of compound 6e.

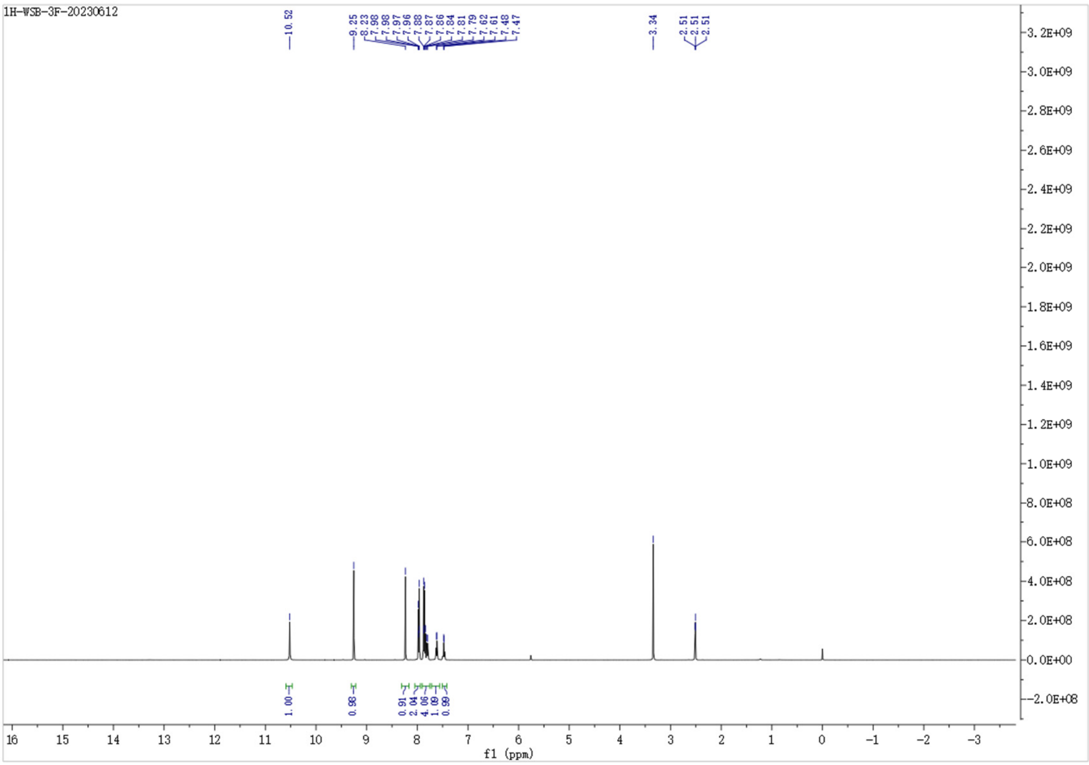

Figure S8-1. ^{1}H-NMR spectrum of compound 6f.

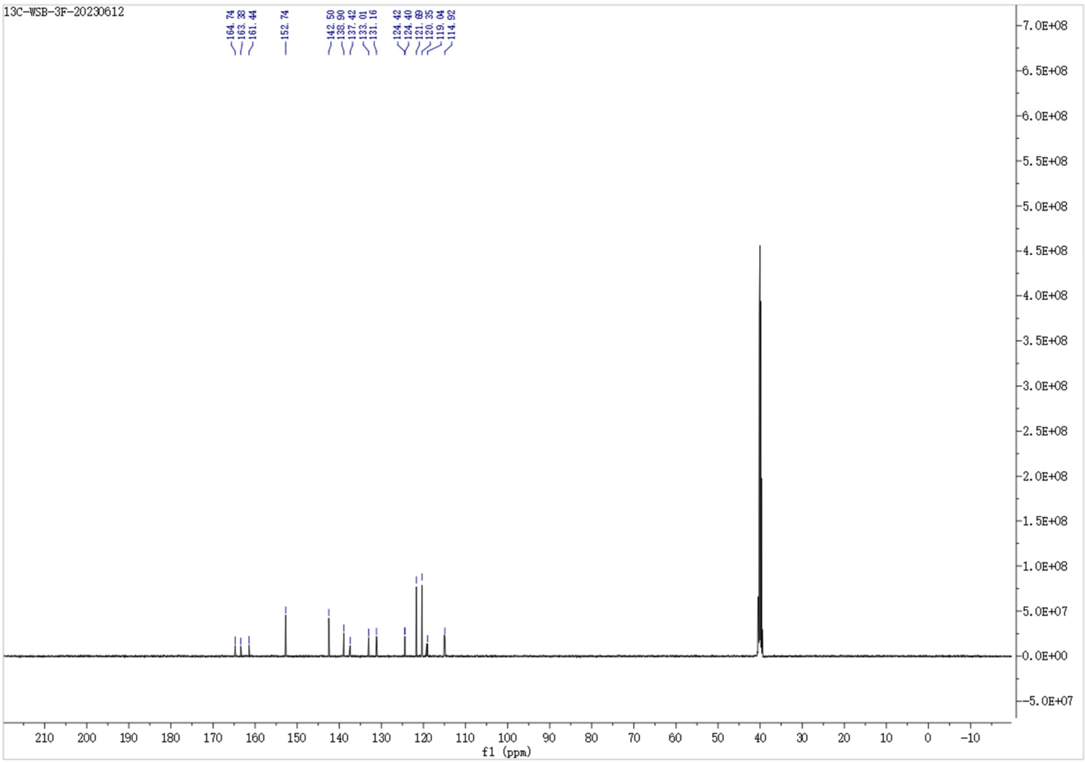

Figure S8-2. ^{13}C-NMR spectrum of compound 6f.

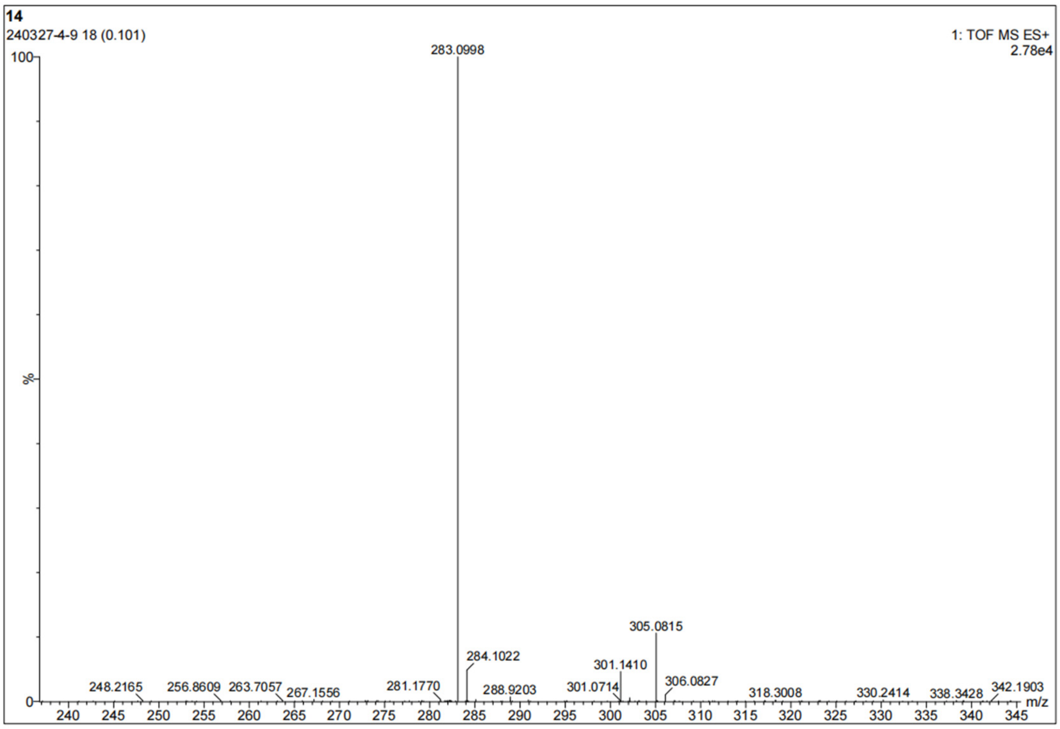

Figure S8-3. HRMS spectrum of compound 6f.

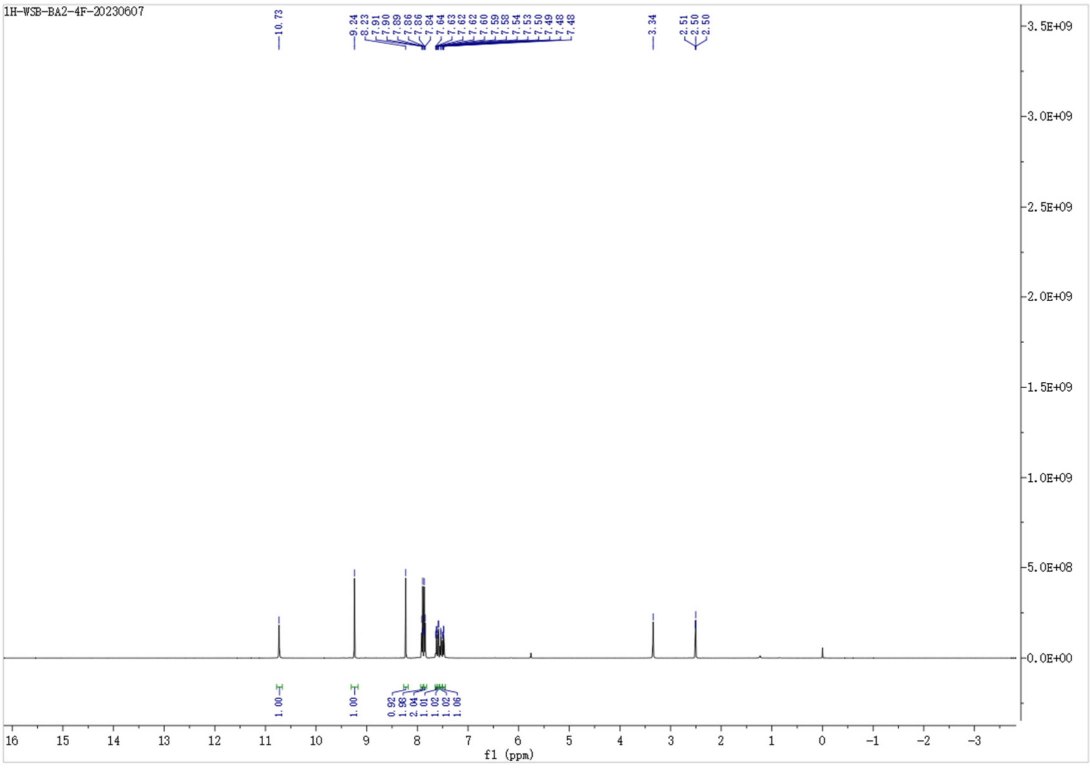

Figure S9-1. **^{1}H-NMR spectrum of compound 6g.**

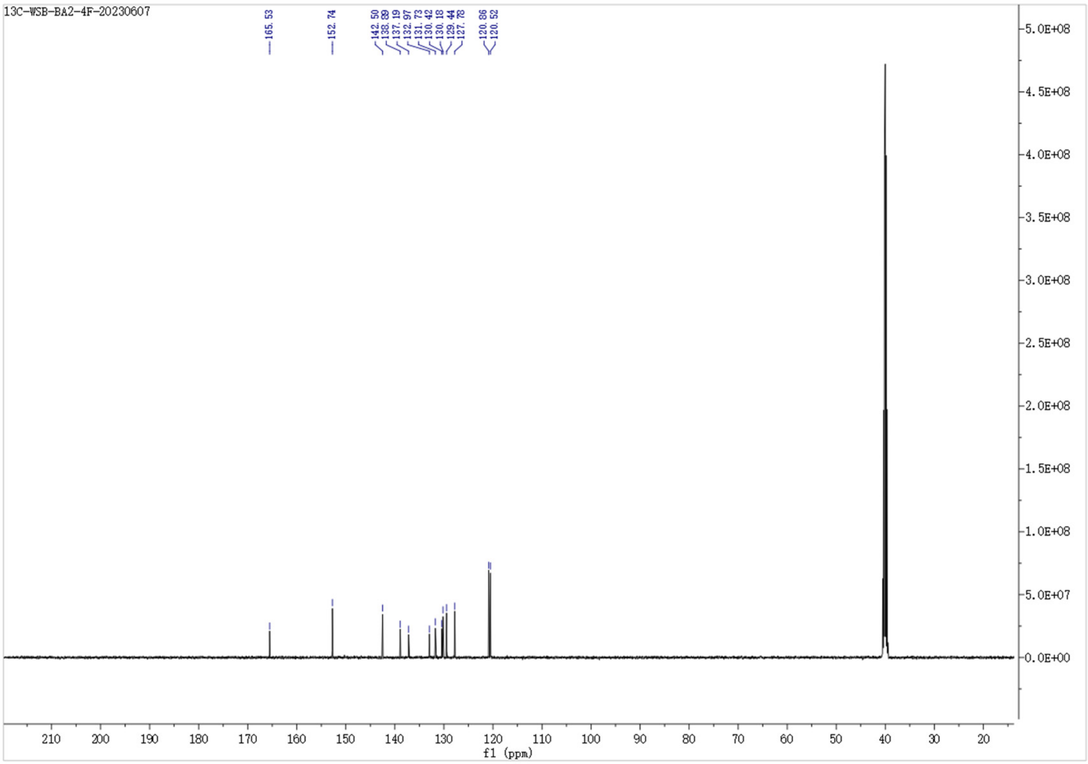

Figure S9-2. **^{13}C-NMR spectrum of compound 6g.**

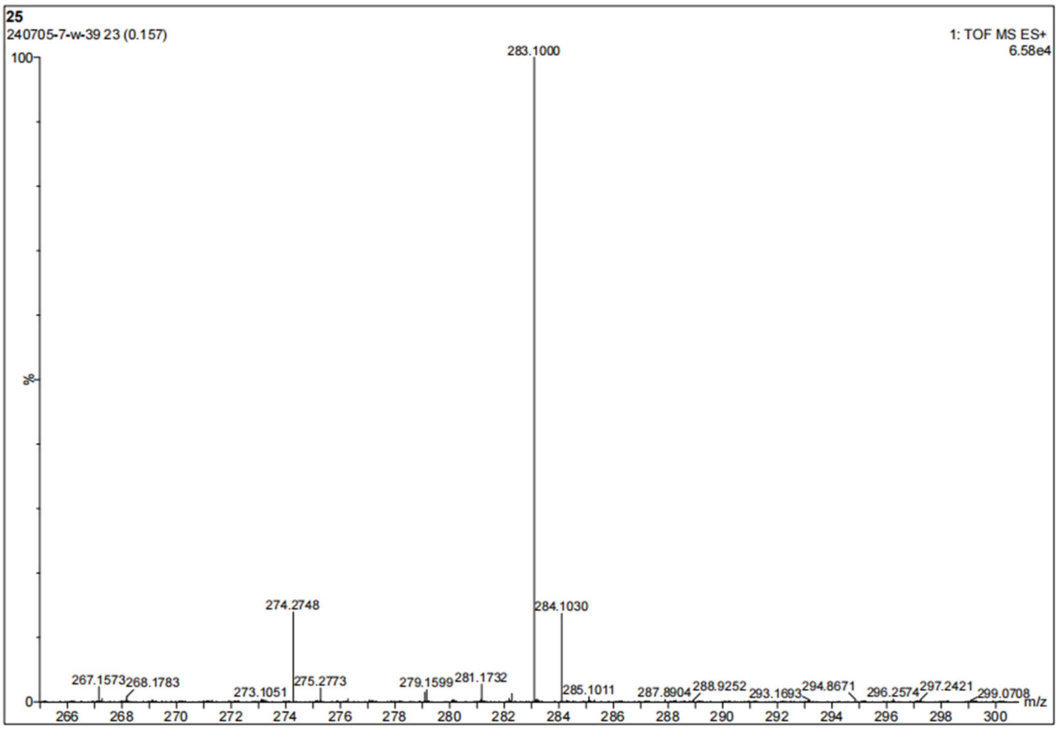

Figure S9-3. HRMS spectrum of compound 6g.

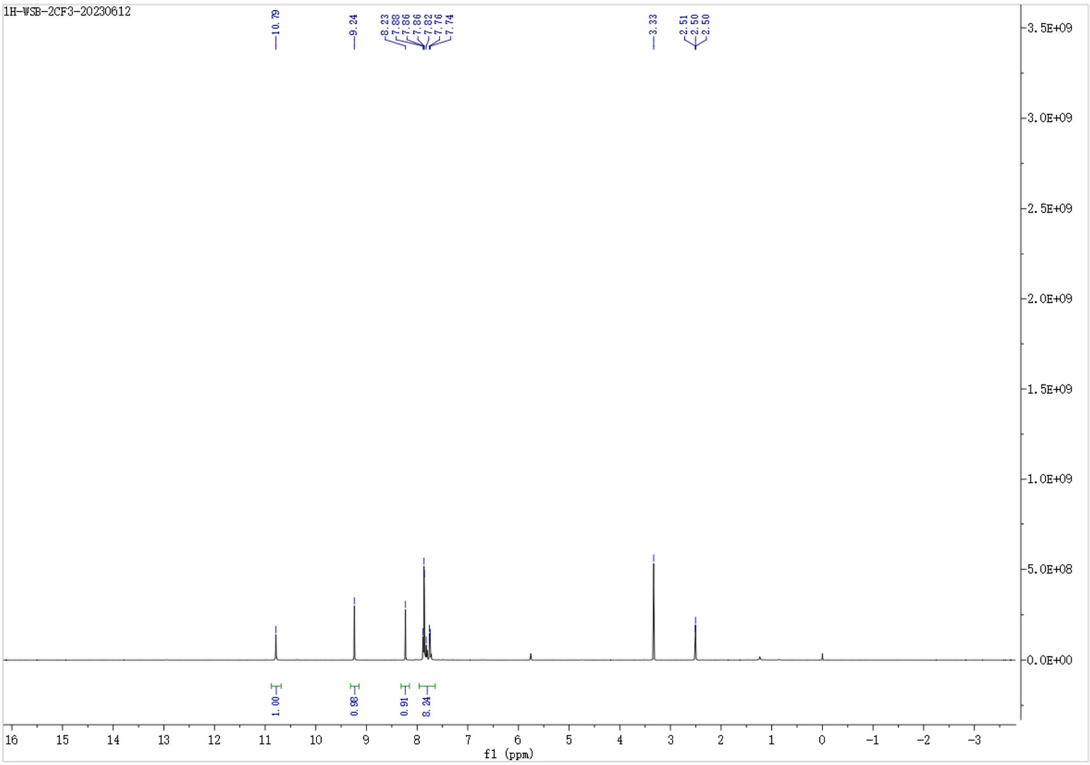

Figure S10-1. ^{1}H-NMR spectrum of compound 6h.

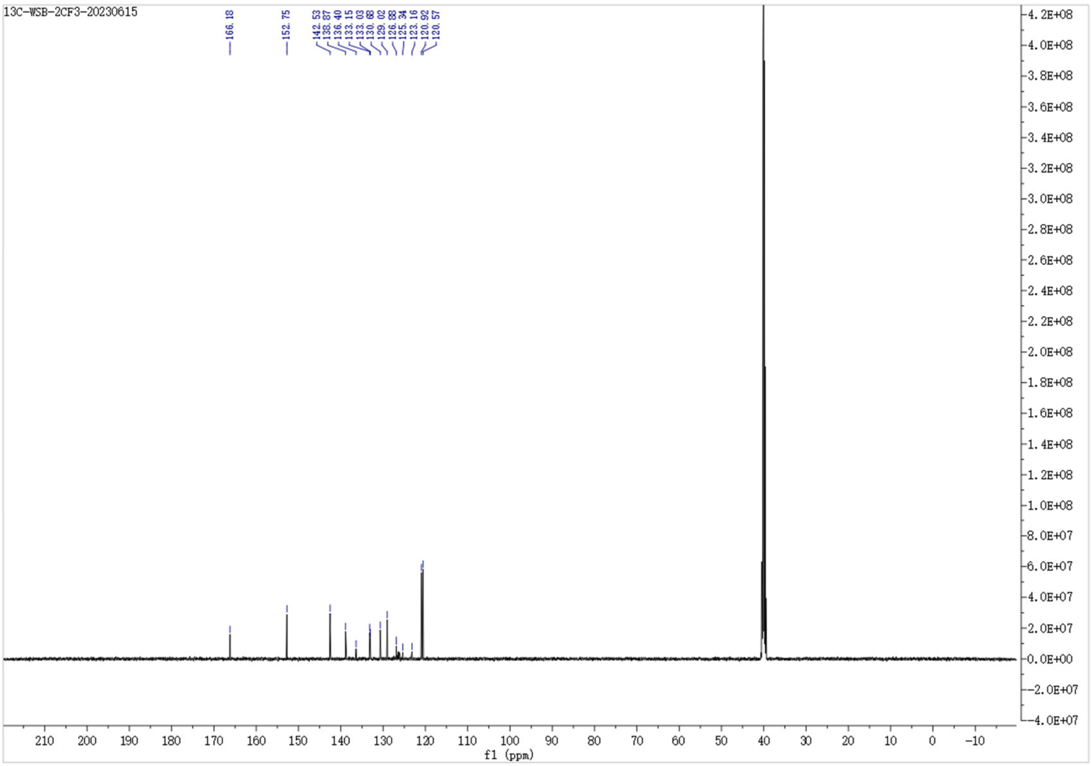

Figure S10-2. ¹³C-NMR spectrum of compound 6h.

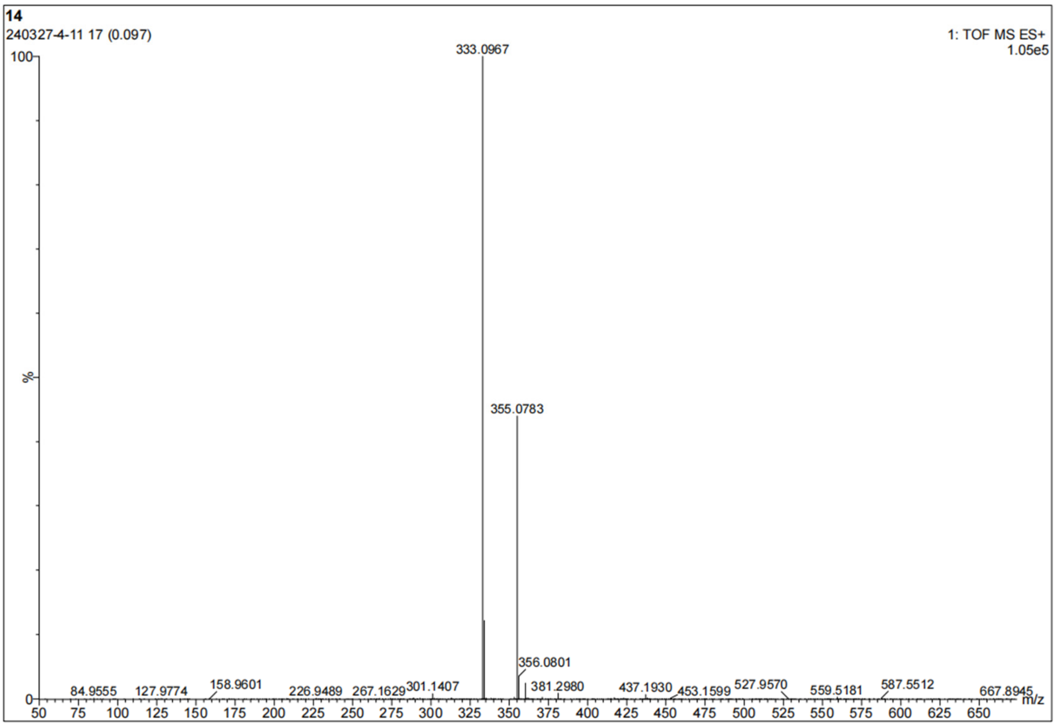

Figure S10-3. HRMS spectrum of compound 6h.

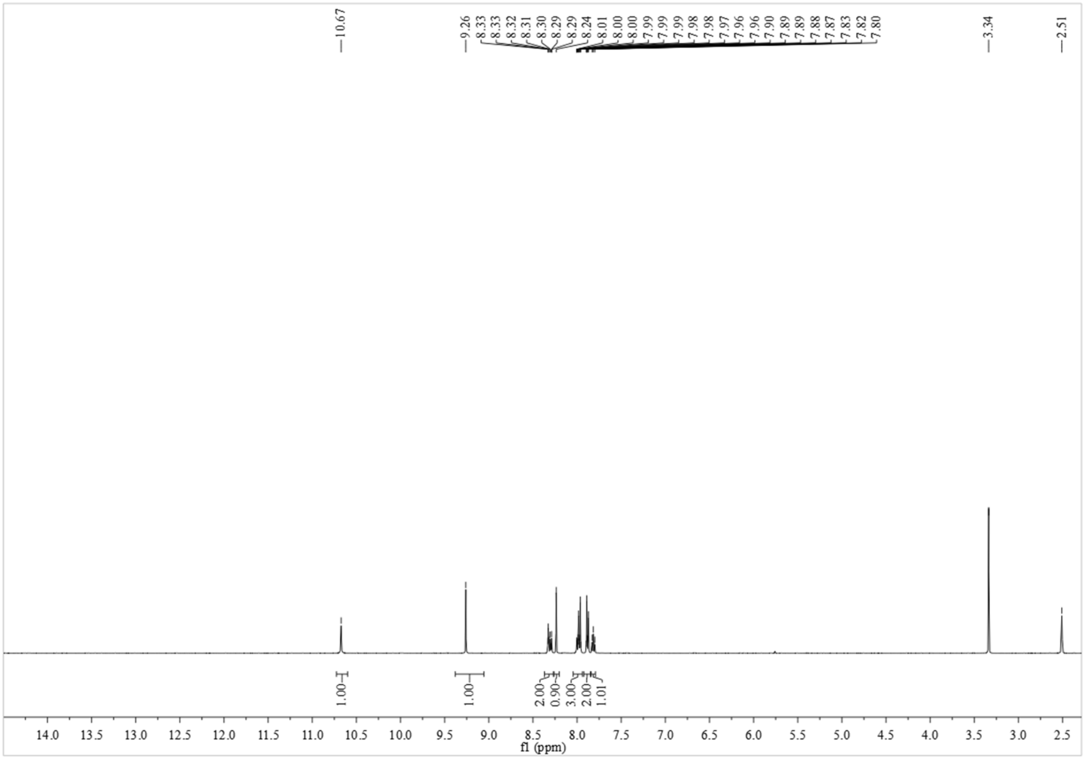

Figure S11-1. **^{1}H-NMR spectrum of compound 6i.**

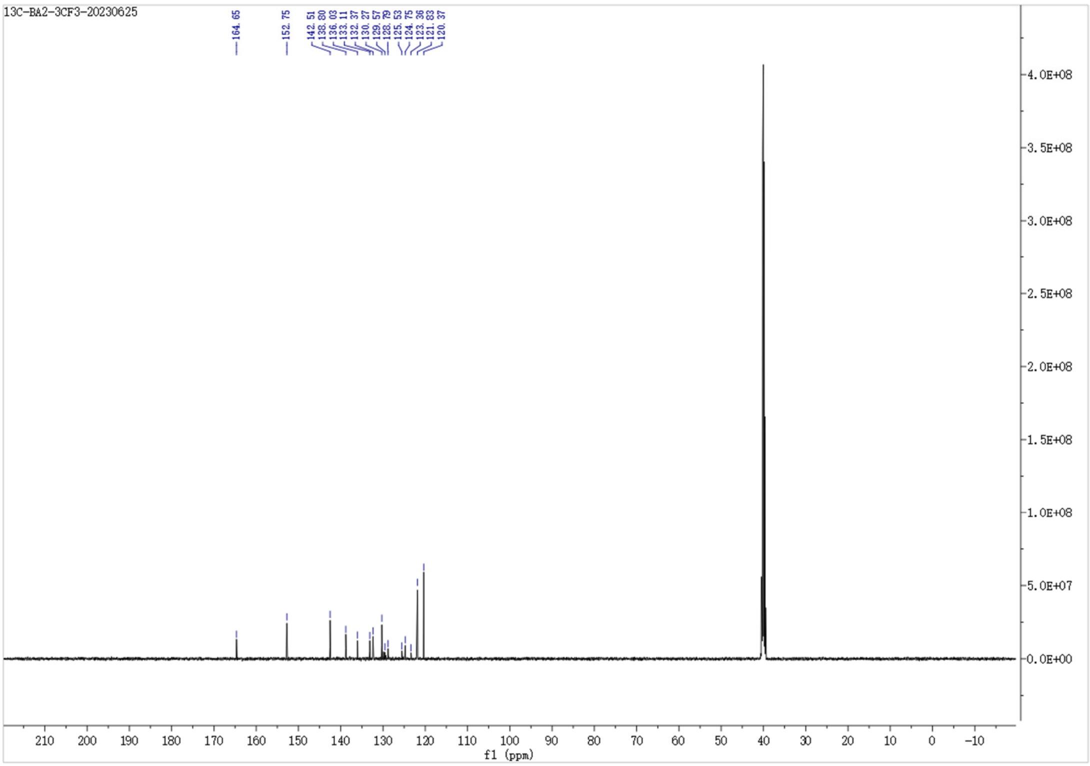

Figure S11-2. **^{13}C-NMR spectrum of compound 6i.**

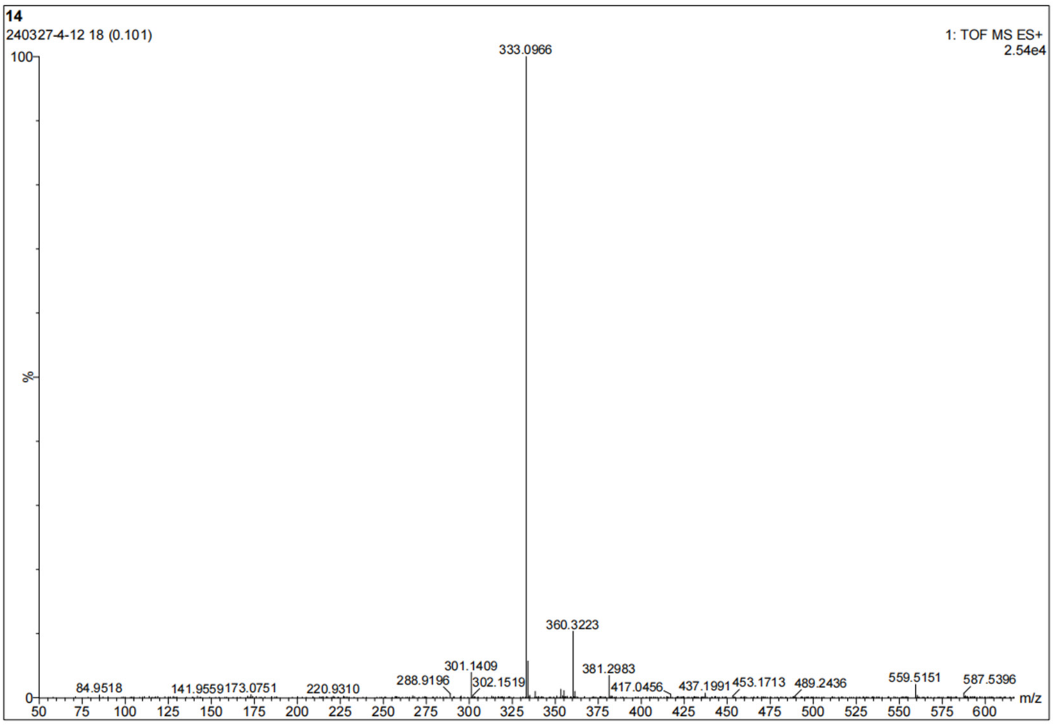

Figure S11-3. HRMS spectrum of compound 6i.

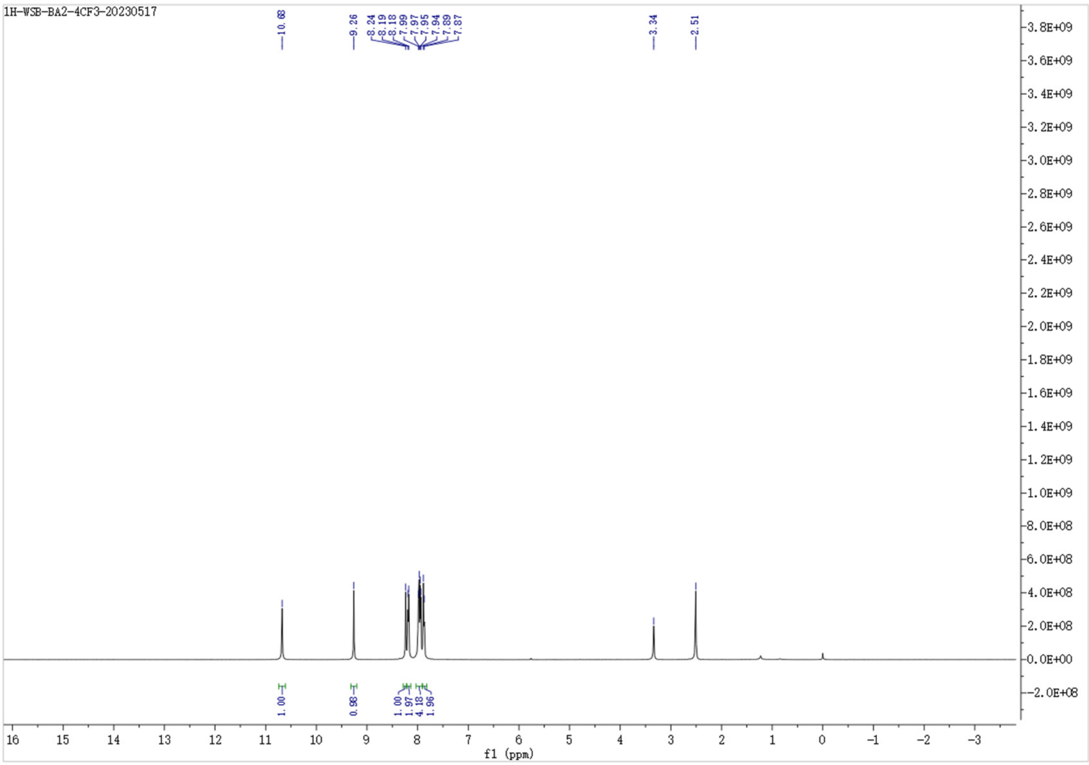

Figure S12-1. ^{1}H-NMR spectrum of compound 6j.

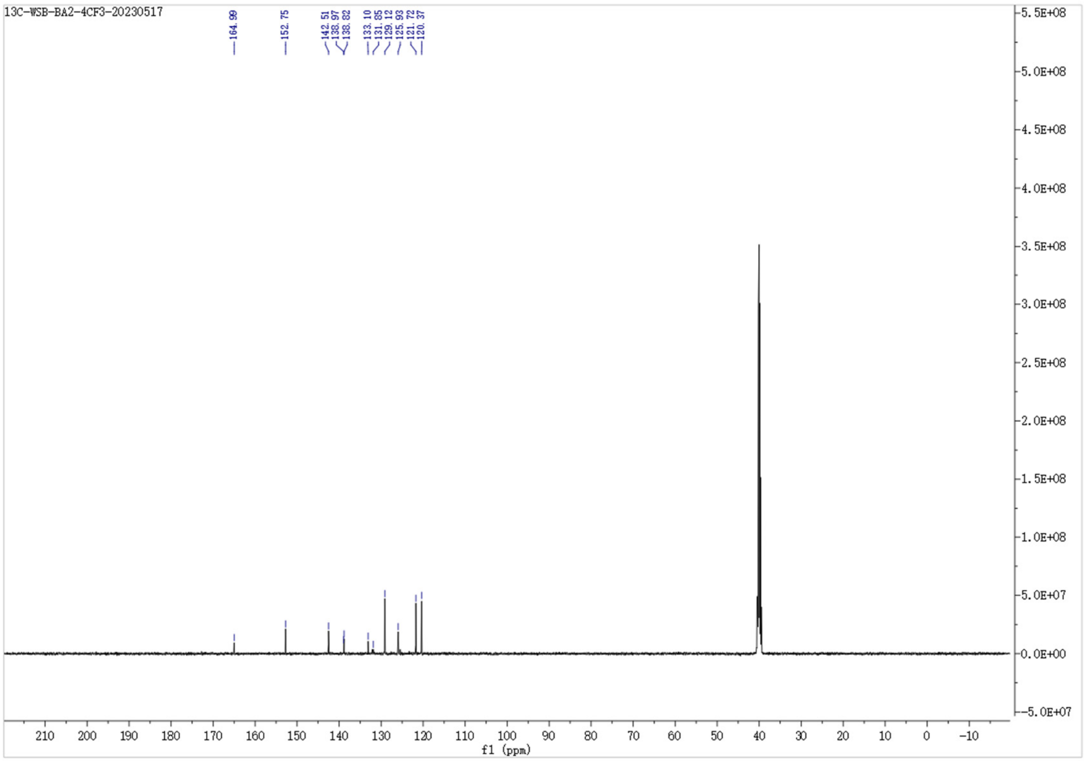

Figure S12-2. ^{13}**C-NMR spectrum of compound 6j.**

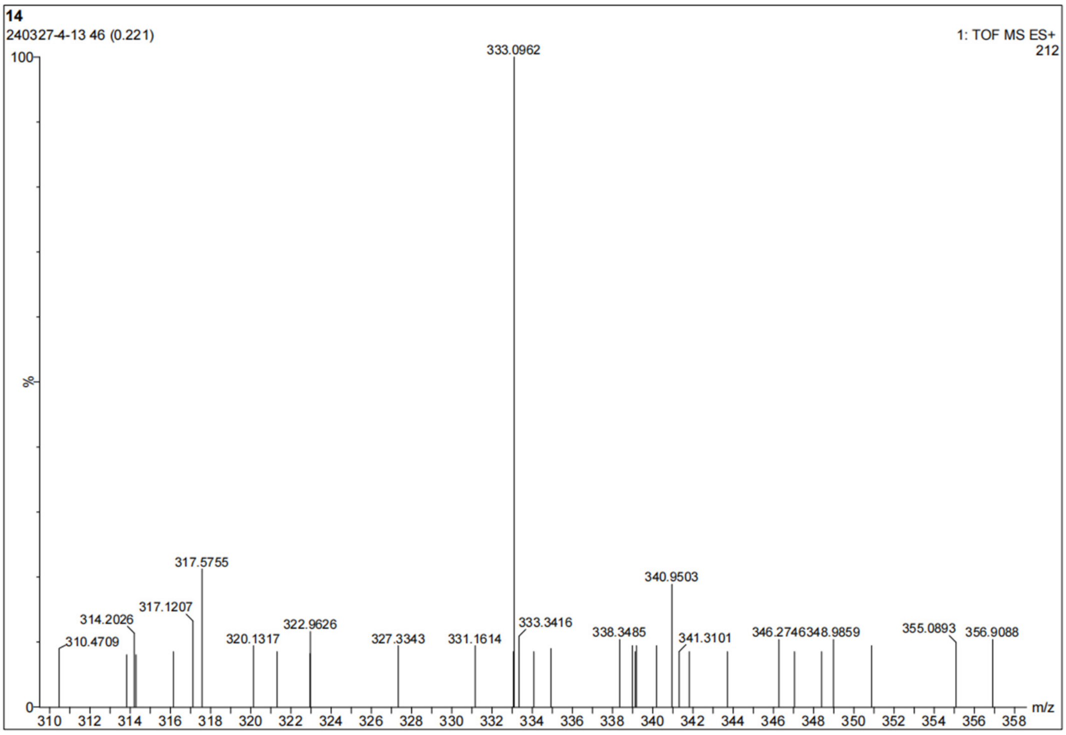

Figure S12-3. HRMS spectrum of compound 6j.

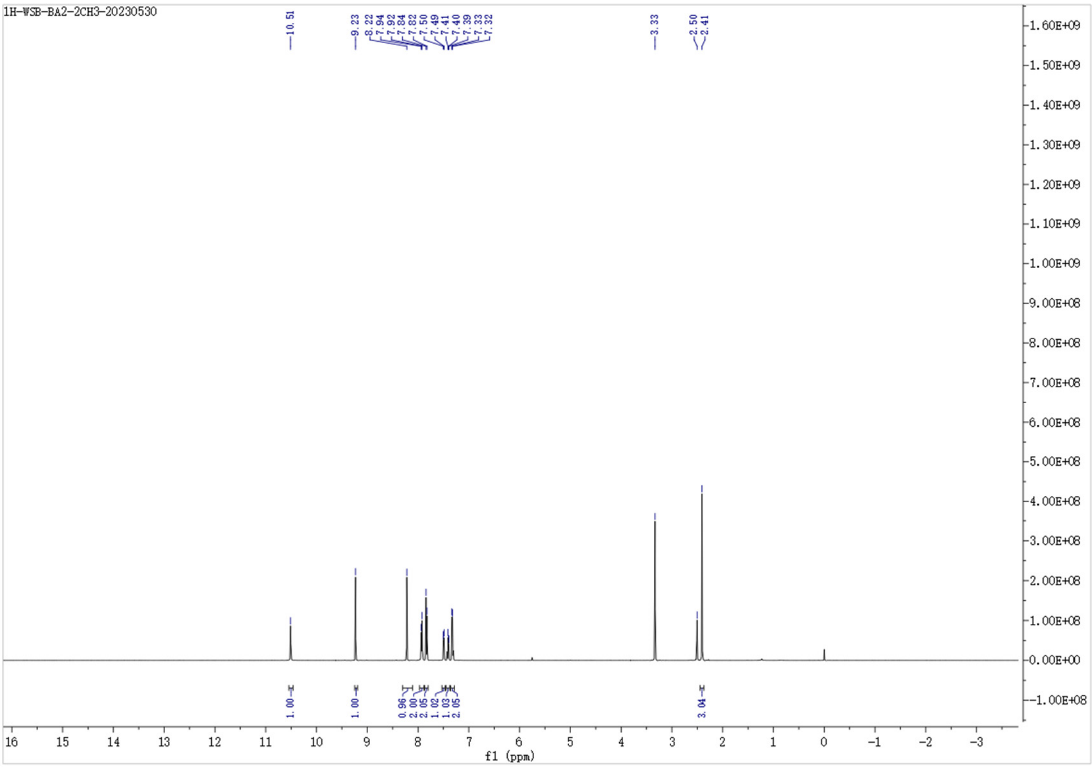

Figure S13-1. **^{1}H-NMR spectrum of compound 6k.**

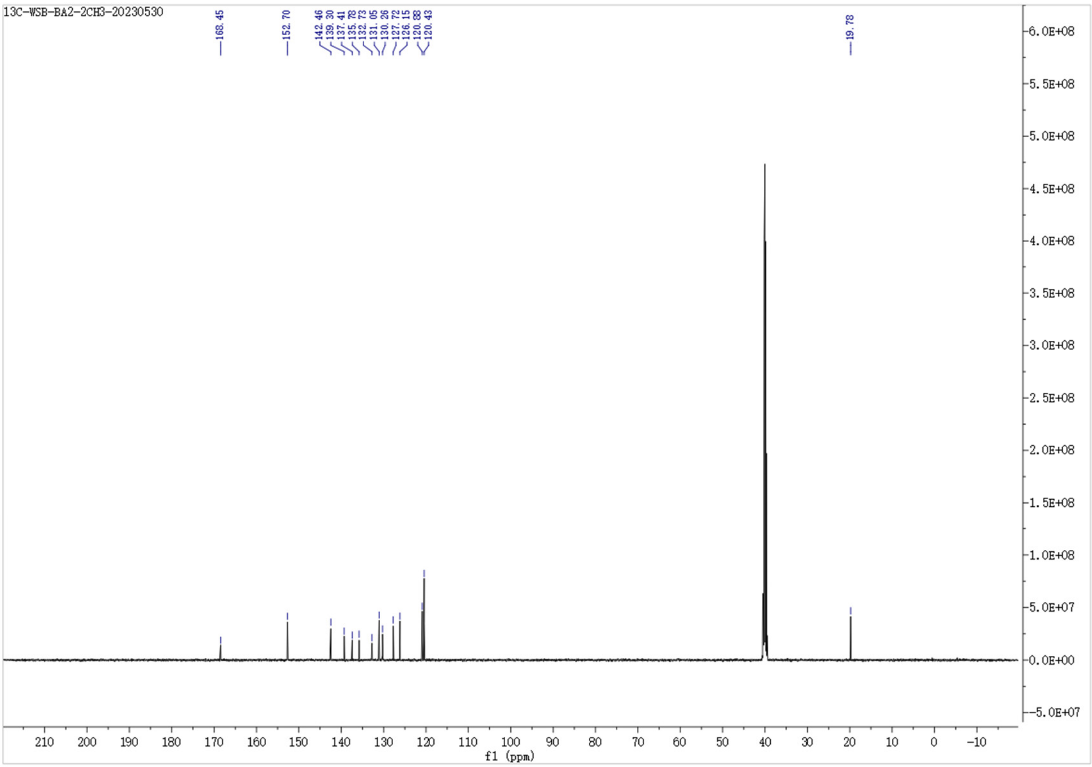

Figure S13-2. **^{13}C-NMR spectrum of compound 6k.**

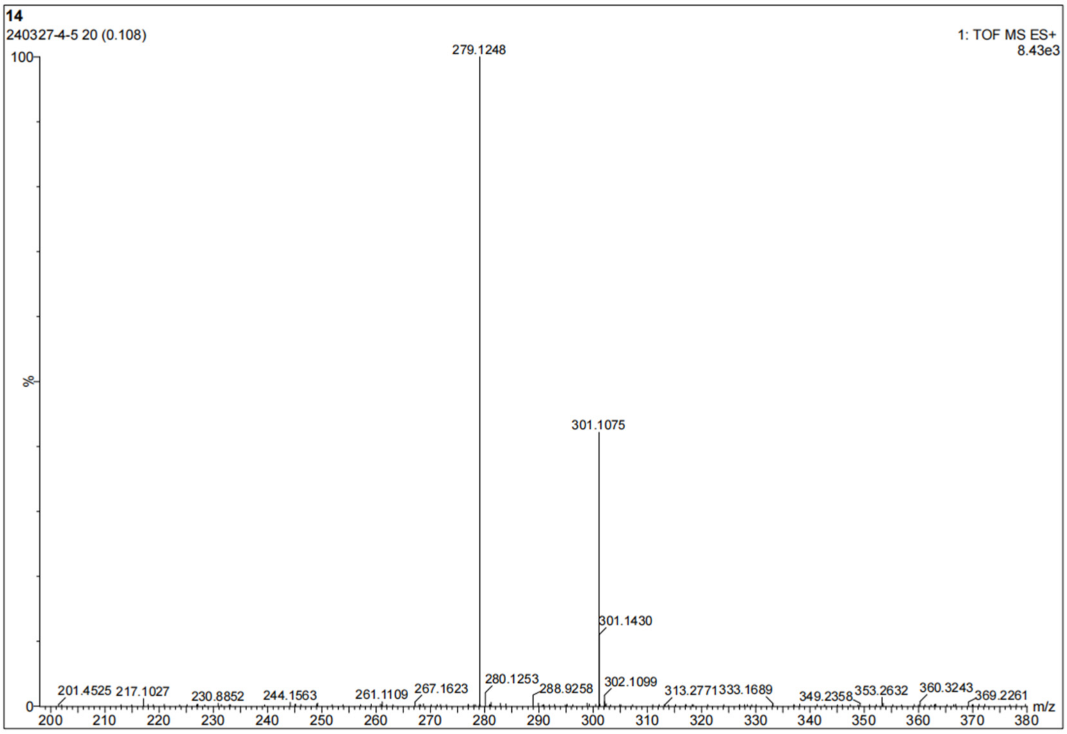

Figure S13-3. HRMS spectrum of compound 6k.

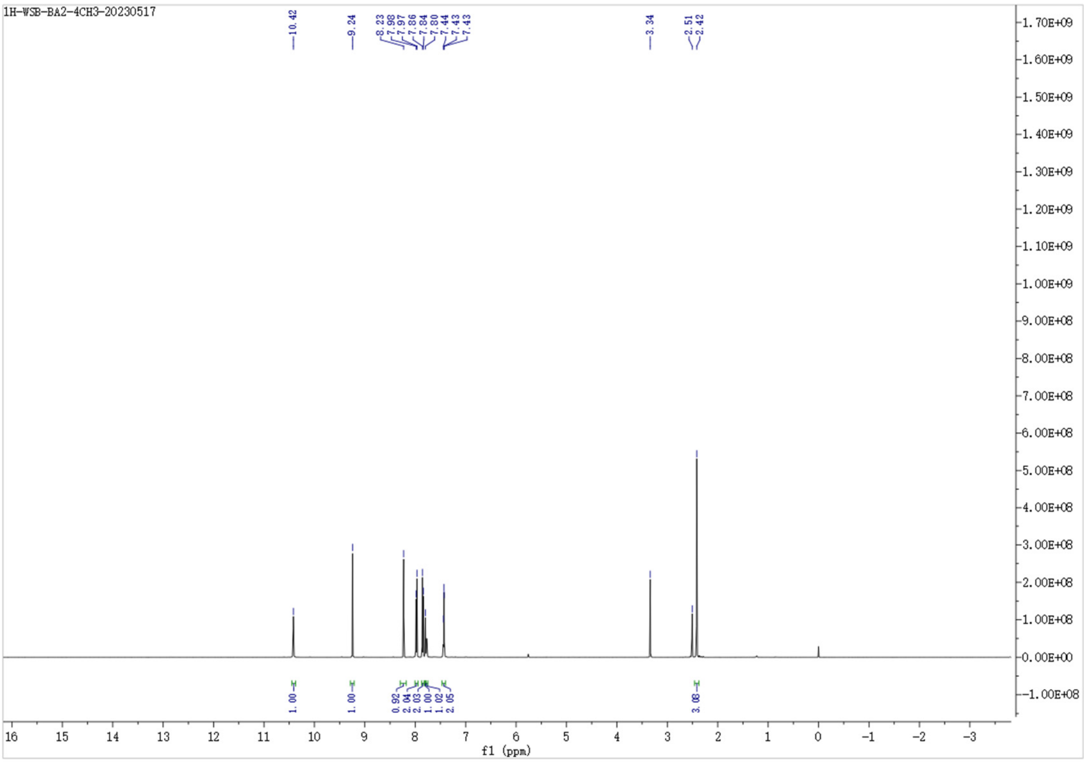

Figure S14-1. ^{1}H-NMR spectrum of compound 6l.

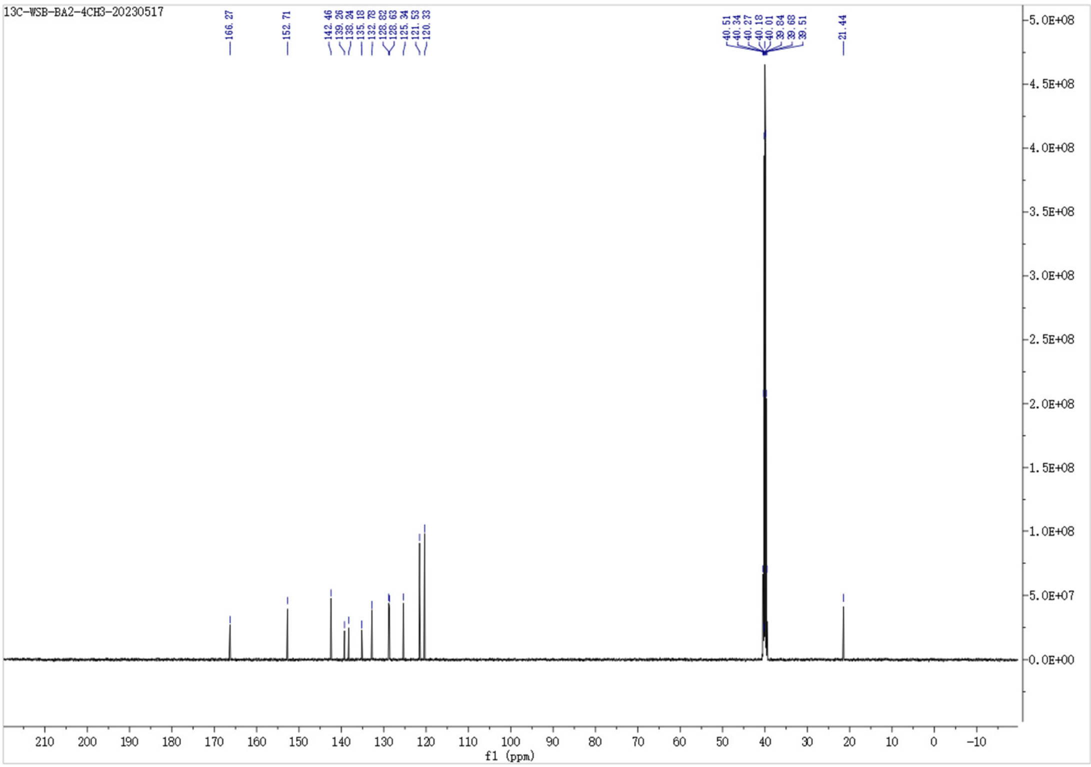

Figure S14-2. ^{13}C-NMR spectrum of compound 6l.

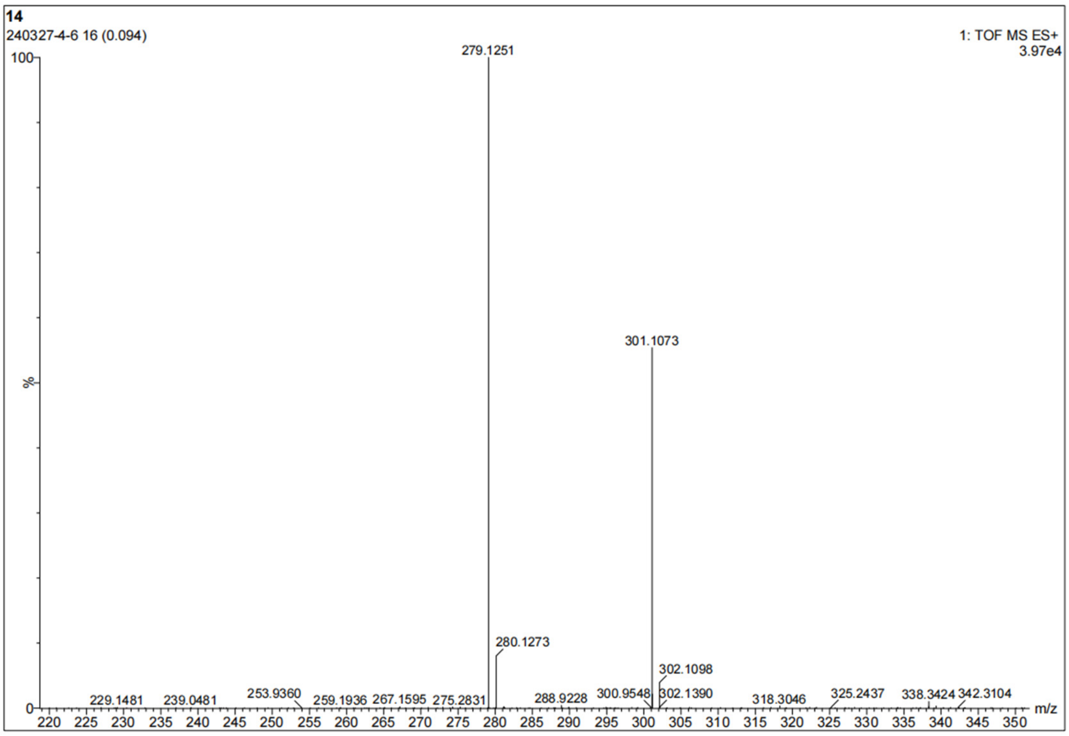

Figure S14-3. HRMS spectrum of compound 6l.

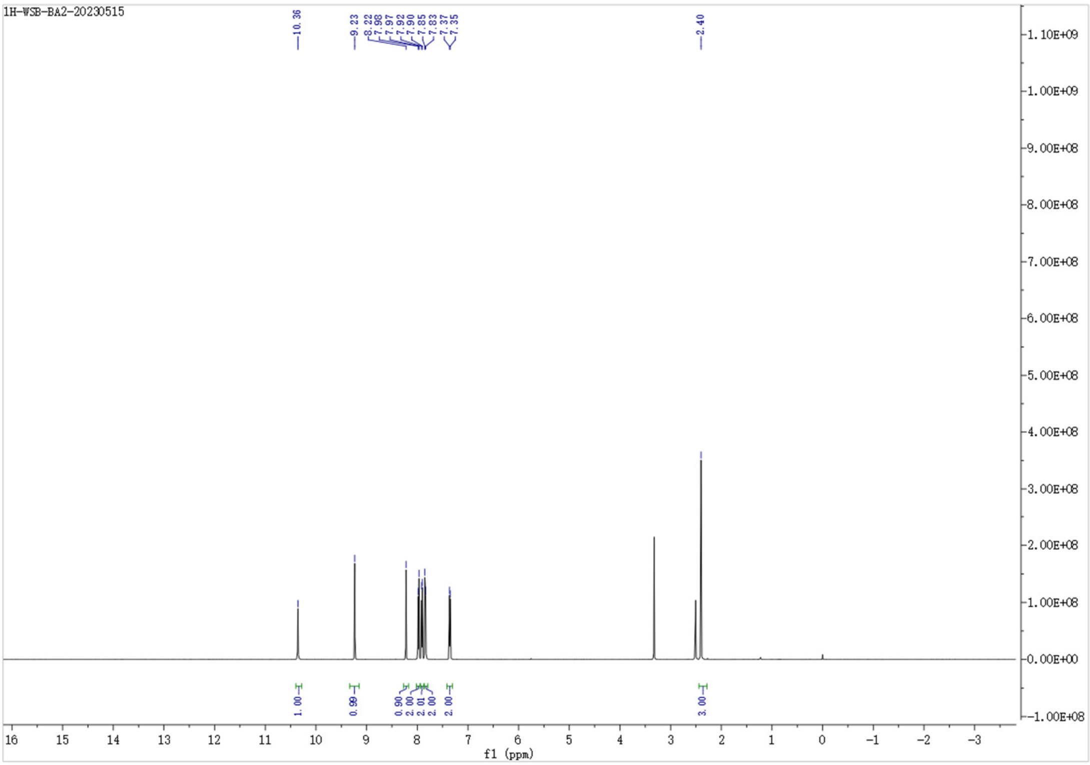

Figure S15-1. ^{1}H-NMR spectrum of compound 6m.

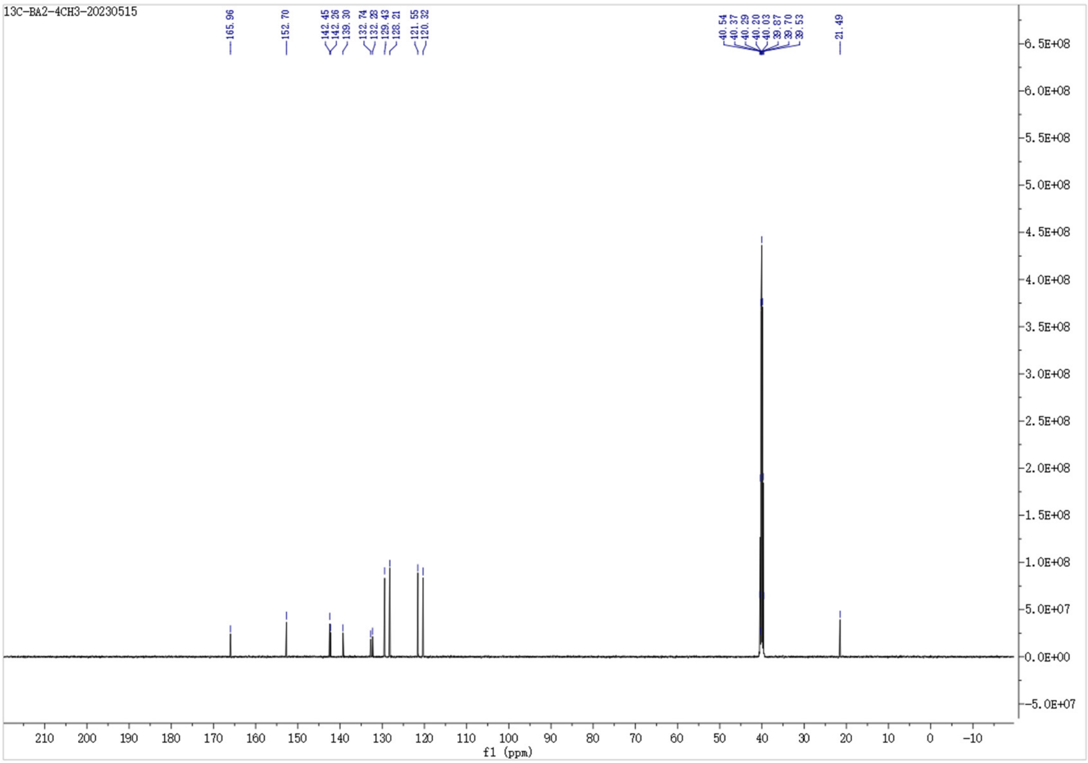

Figure S15-2. ^{13}C-NMR spectrum of compound 6m.

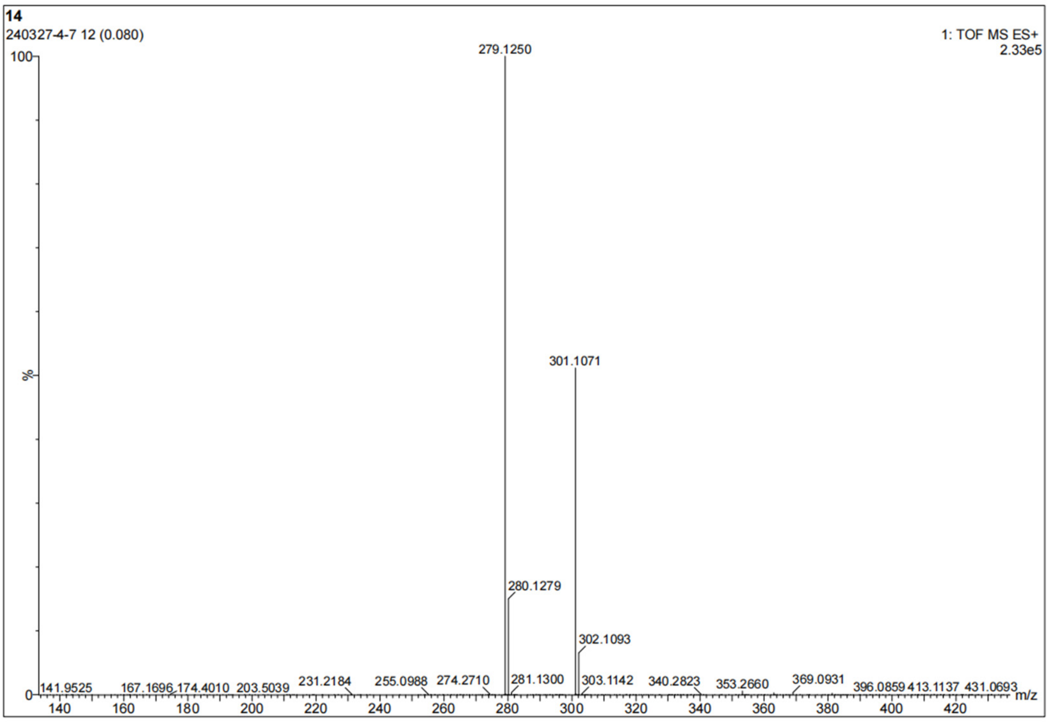

Figure S15-3. HRMS spectrum of compound 6m.

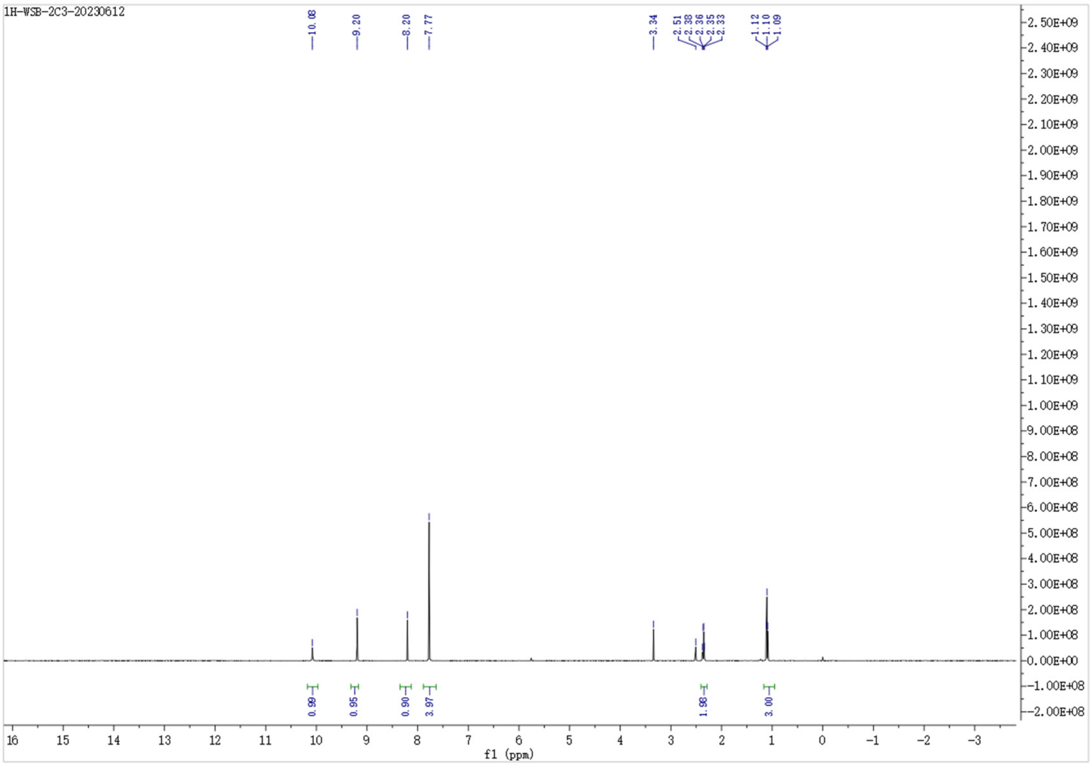

Figure S16-1. ^{1}H-NMR spectrum of compound 9a.

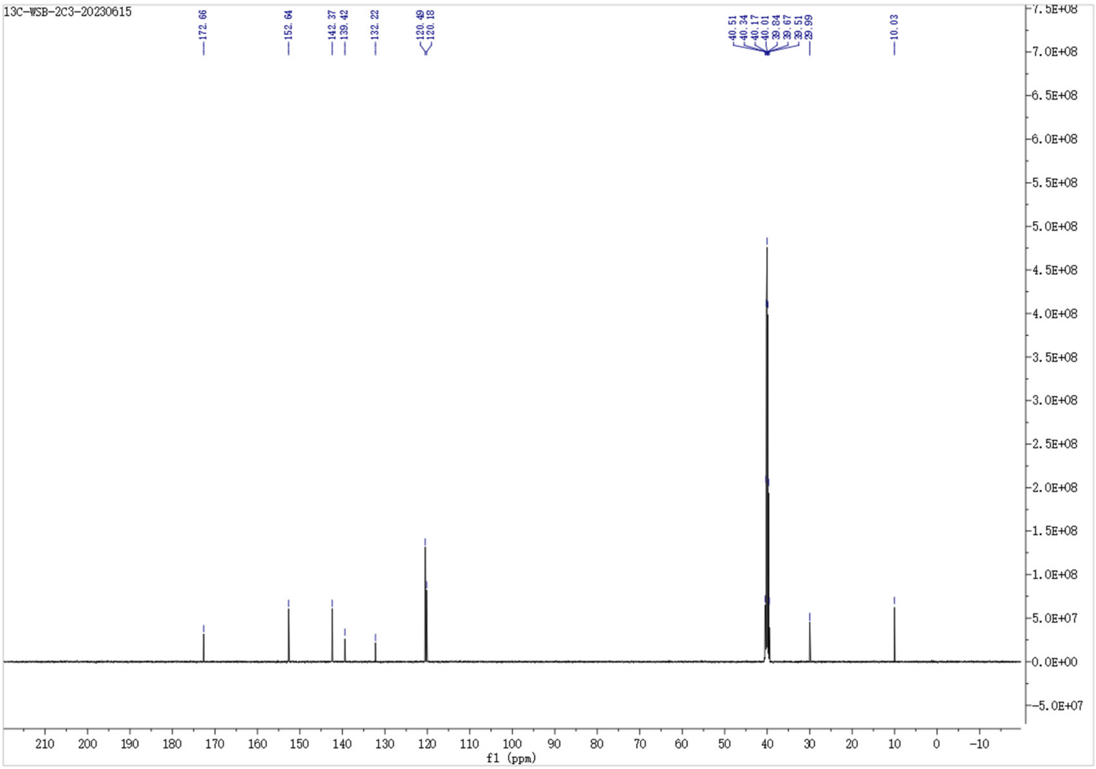

Figure S16-2. ^{13}C-NMR spectrum of compound 9a.

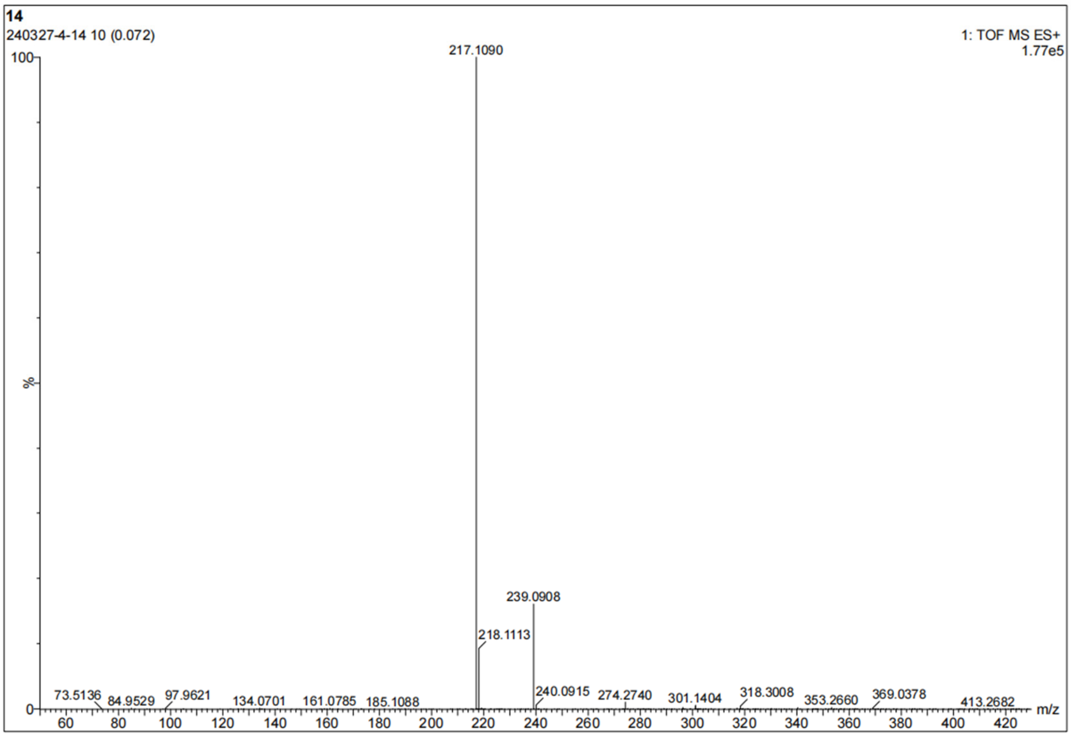

Figure S16-3. HRMS spectrum of compound 9a.

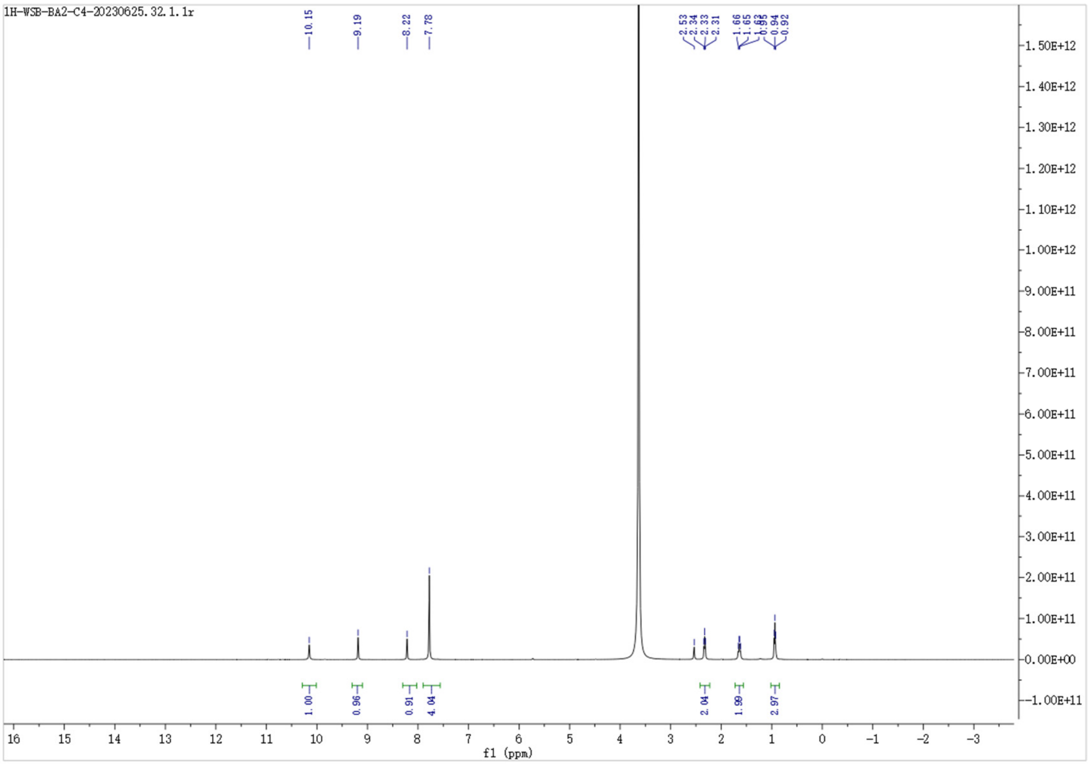

Figure S17-1. ^{1}H-NMR spectrum of compound 9b.

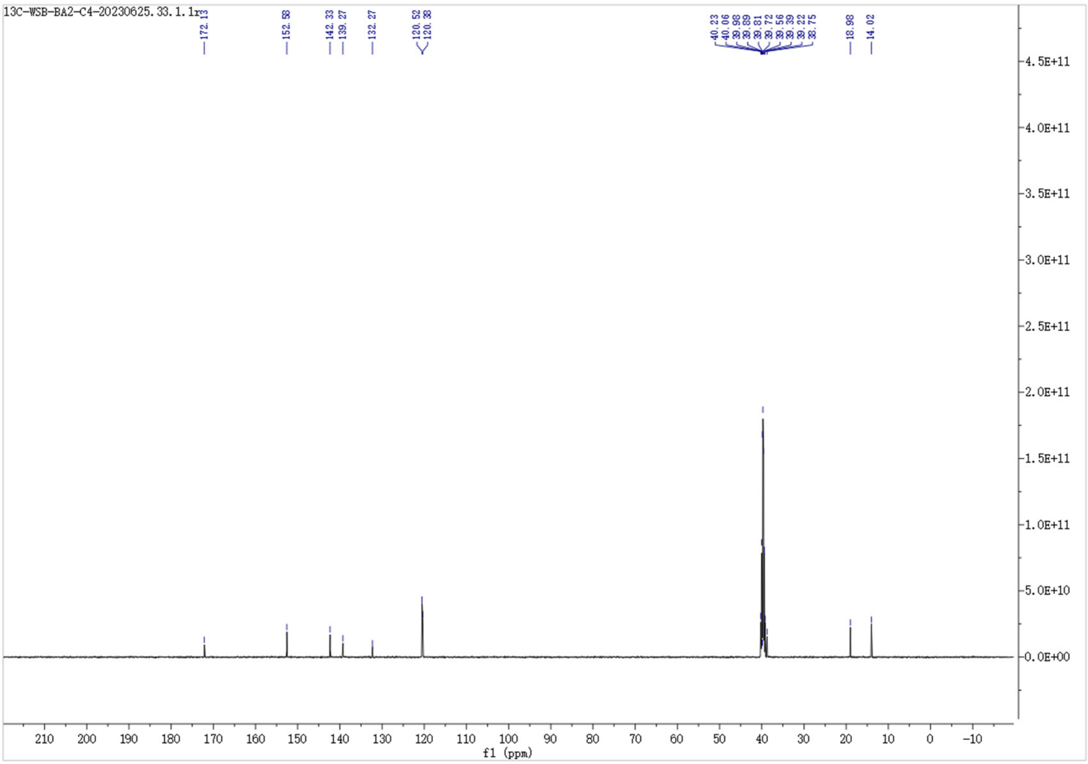

Figure S17-2. ^{13}C-NMR spectrum of compound 9b.

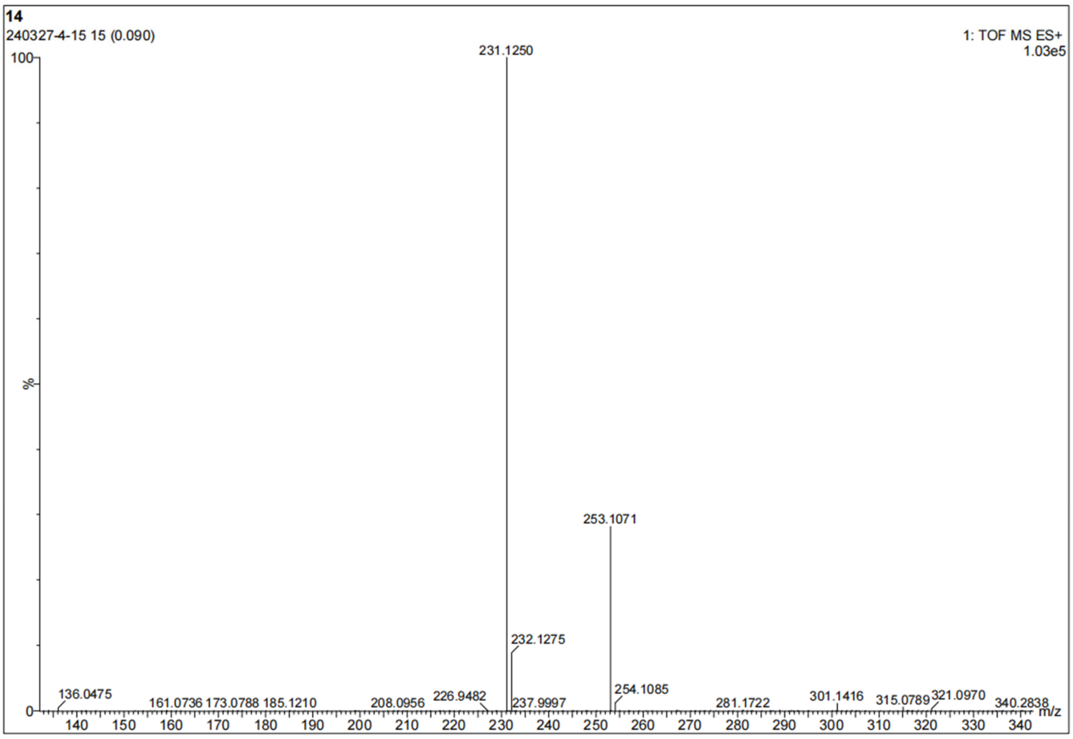

Figure S17-3. HRMS spectrum of compound 9b.

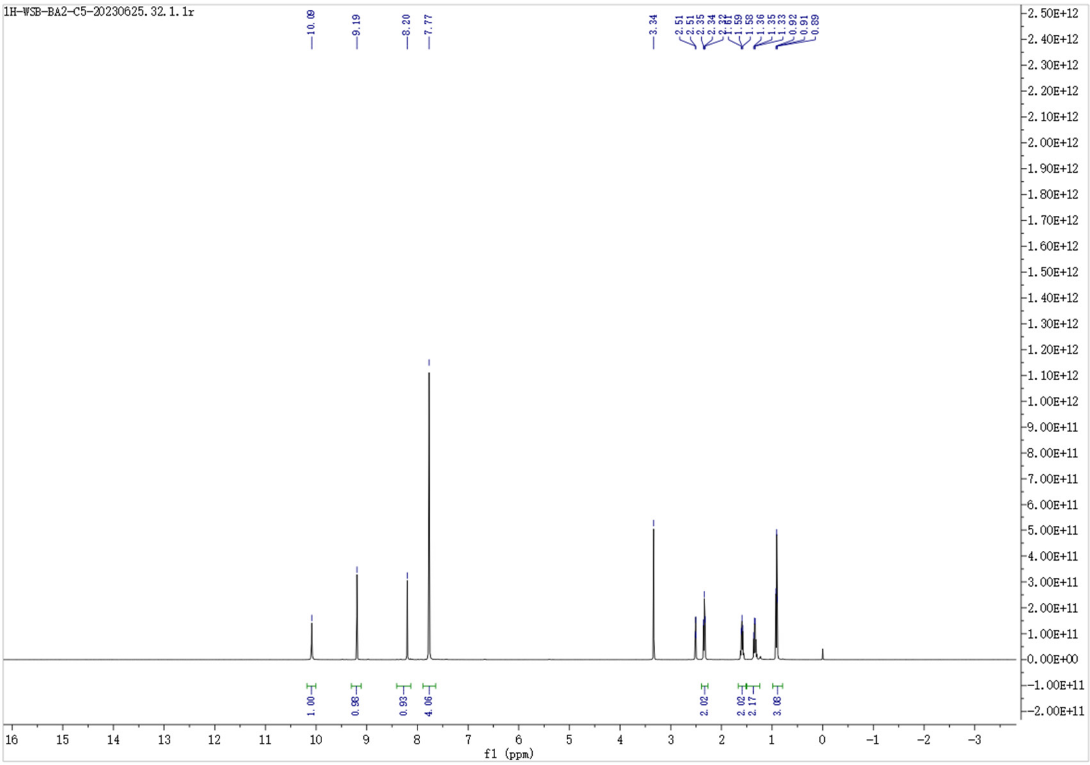

Figure S18-1. ^{1}H-NMR spectrum of compound 9c.

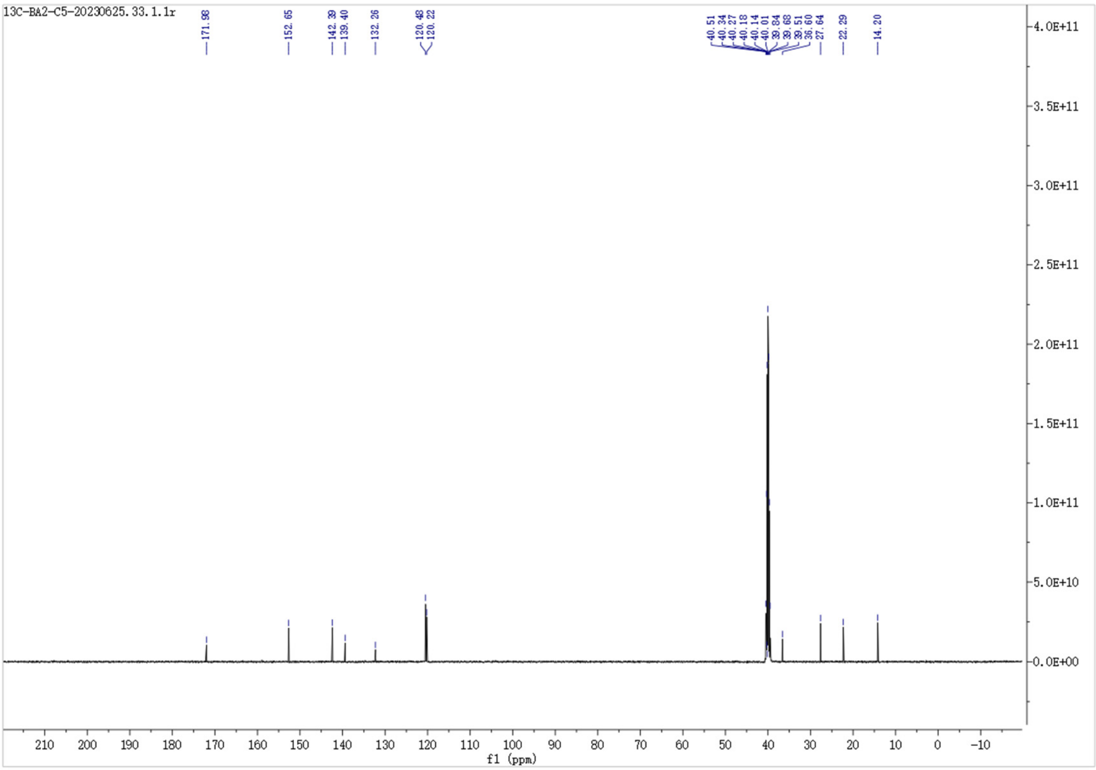

Figure S18-2. ^{13}C**-NMR spectrum of compound 9c.**

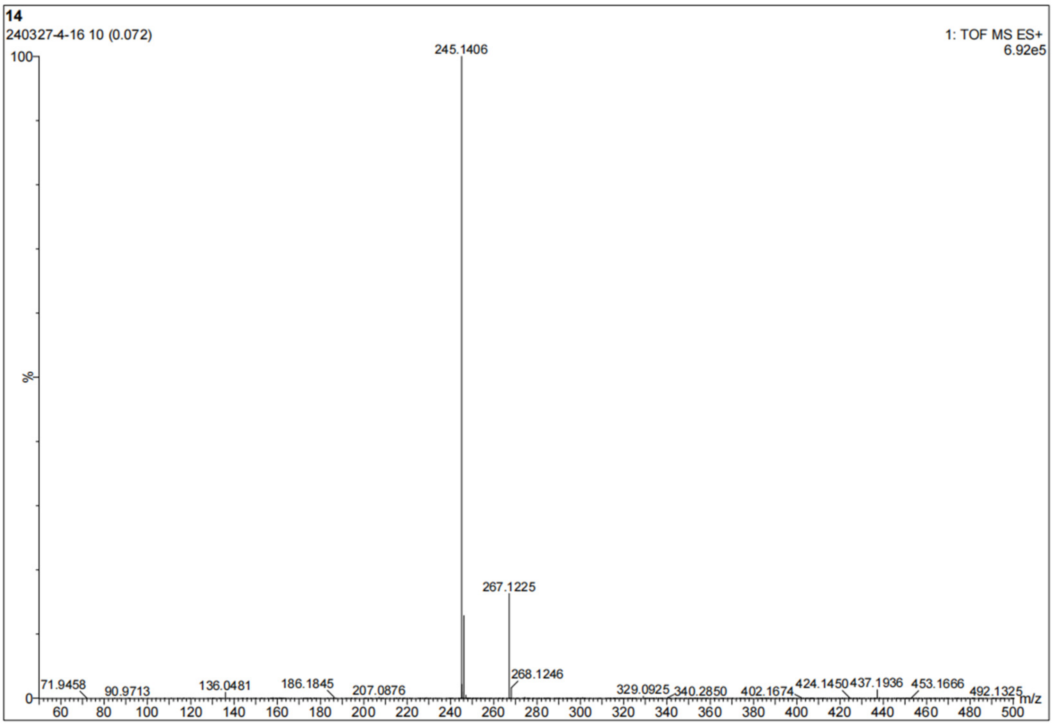

Figure S18-3. HRMS spectrum of compound 9c.

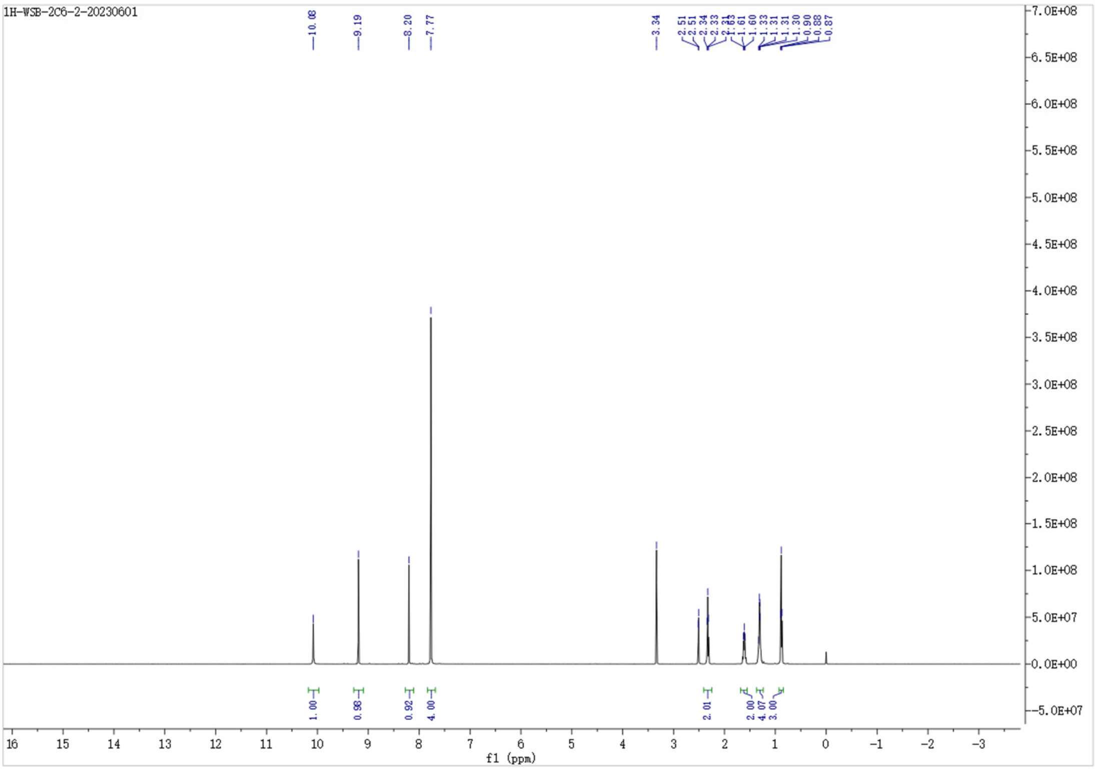

Figure S19-1. **^{1}H-NMR spectrum of compound 9d.**

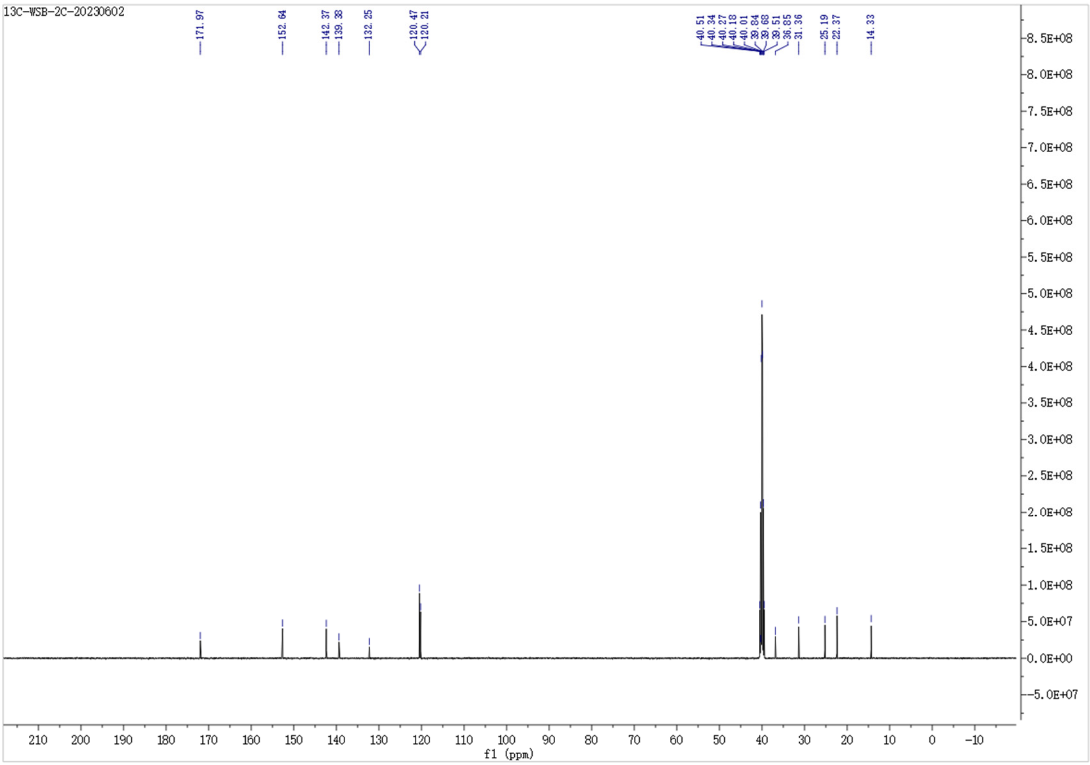

Figure S19-2. **^{13}C-NMR spectrum of compound 9d.**

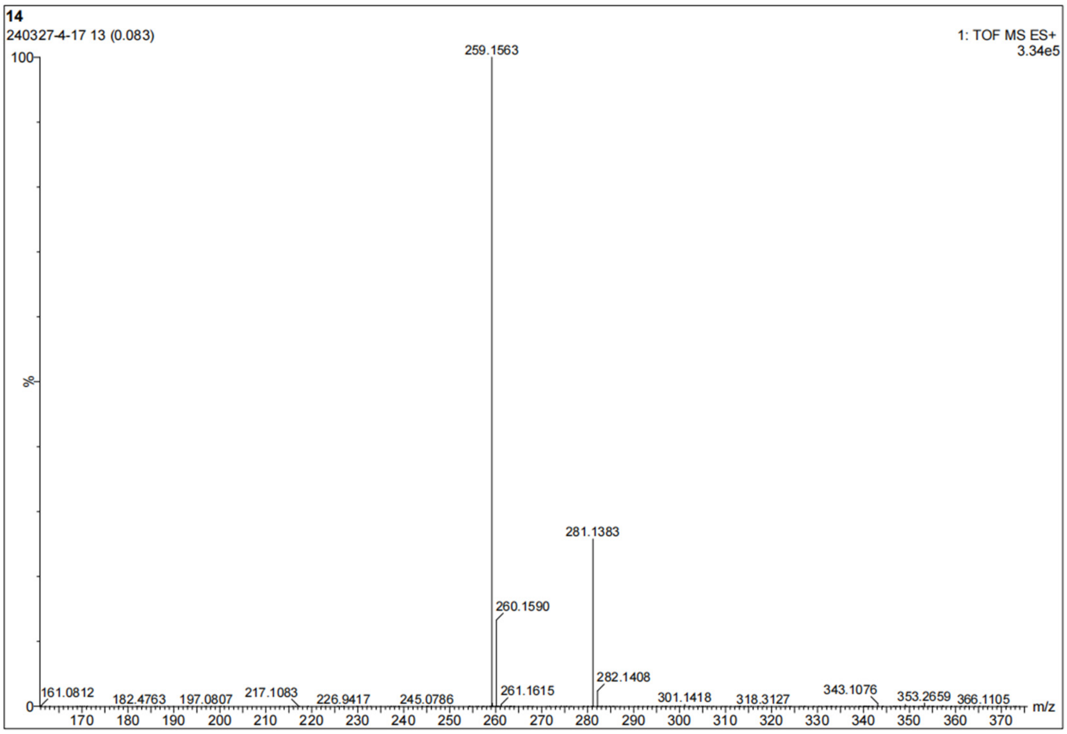

Figure S19-3. HRMS spectrum of compound 9d.

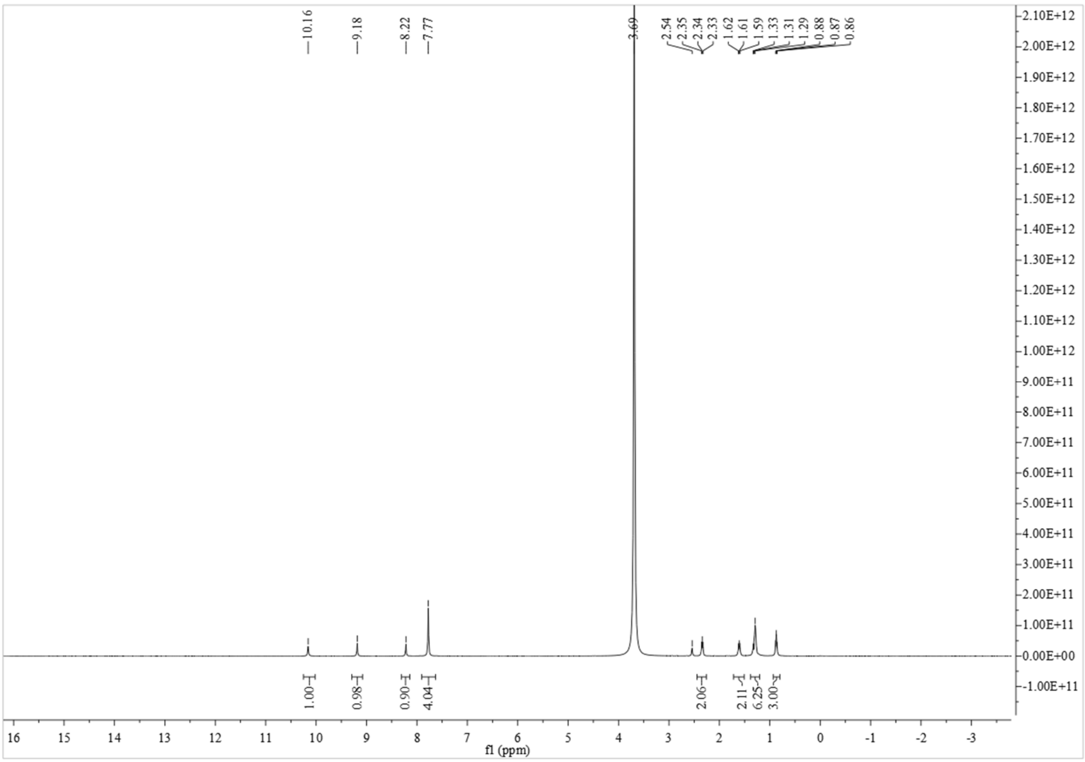

Figure S20-1. ^{1}H-NMR spectrum of compound 9e.

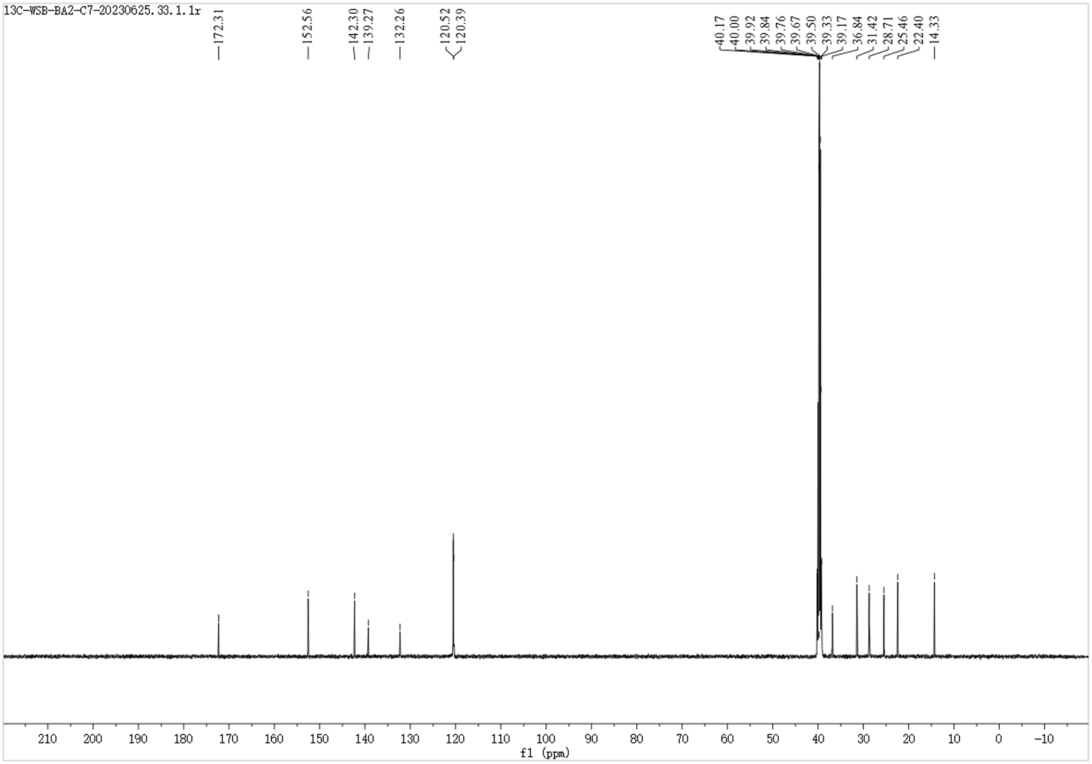

Figure S20-2. ^{13}C-NMR spectrum of compound 9e.

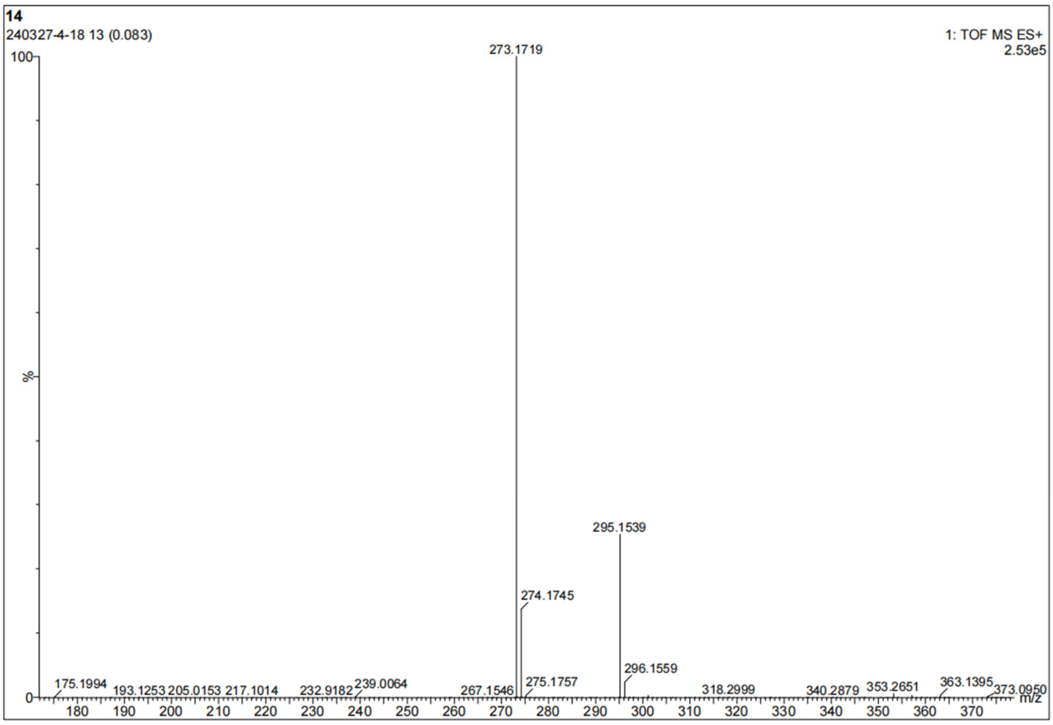

Figure S20-3. HRMS spectrum of compound 9e.

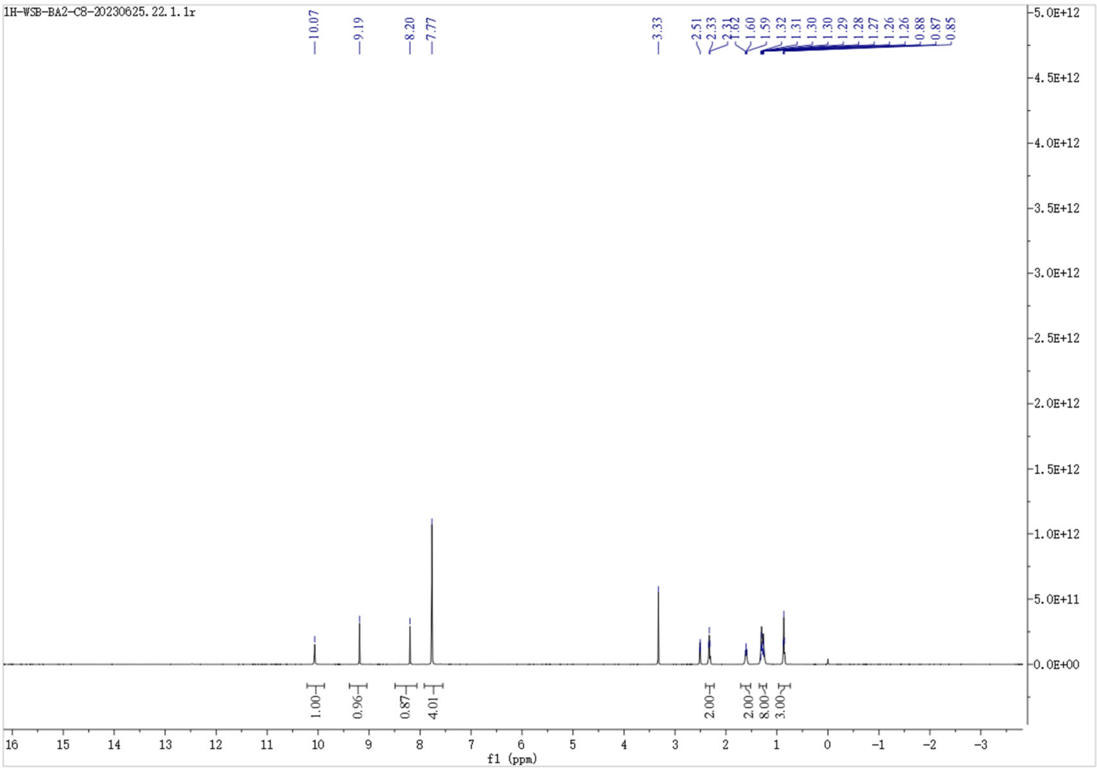

Figure S21-1. ¹H-NMR spectrum of compound 9f.

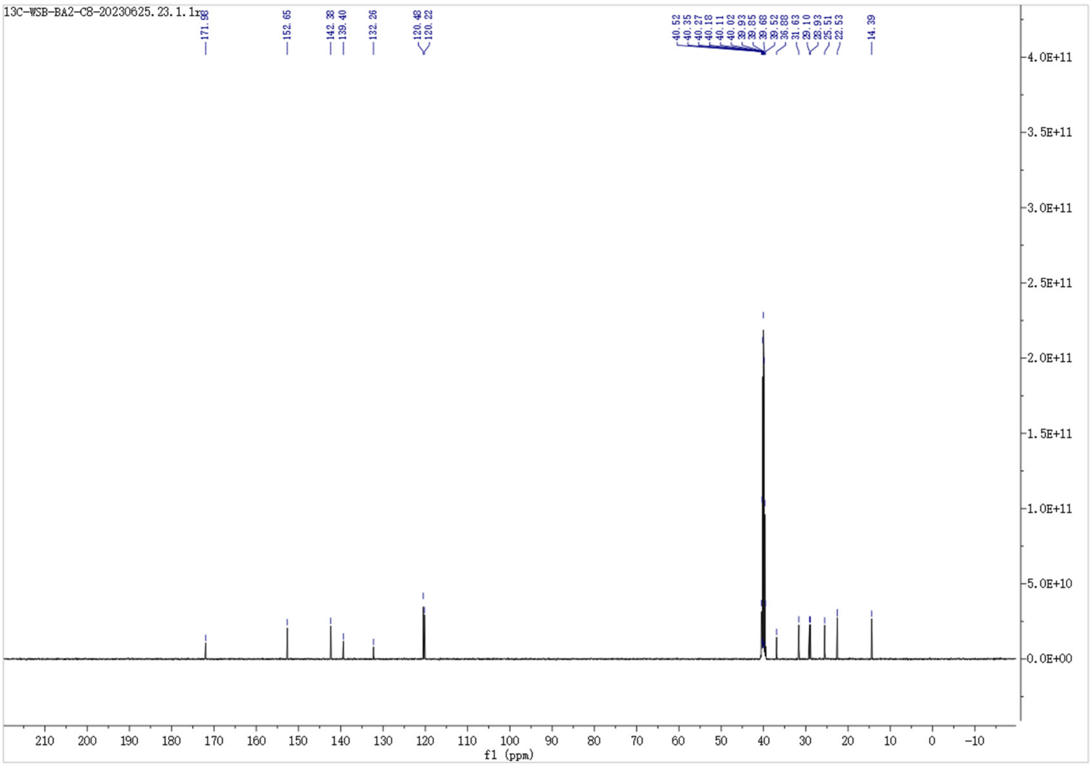

Figure S21-2. ¹³C-NMR spectrum of compound 9f.

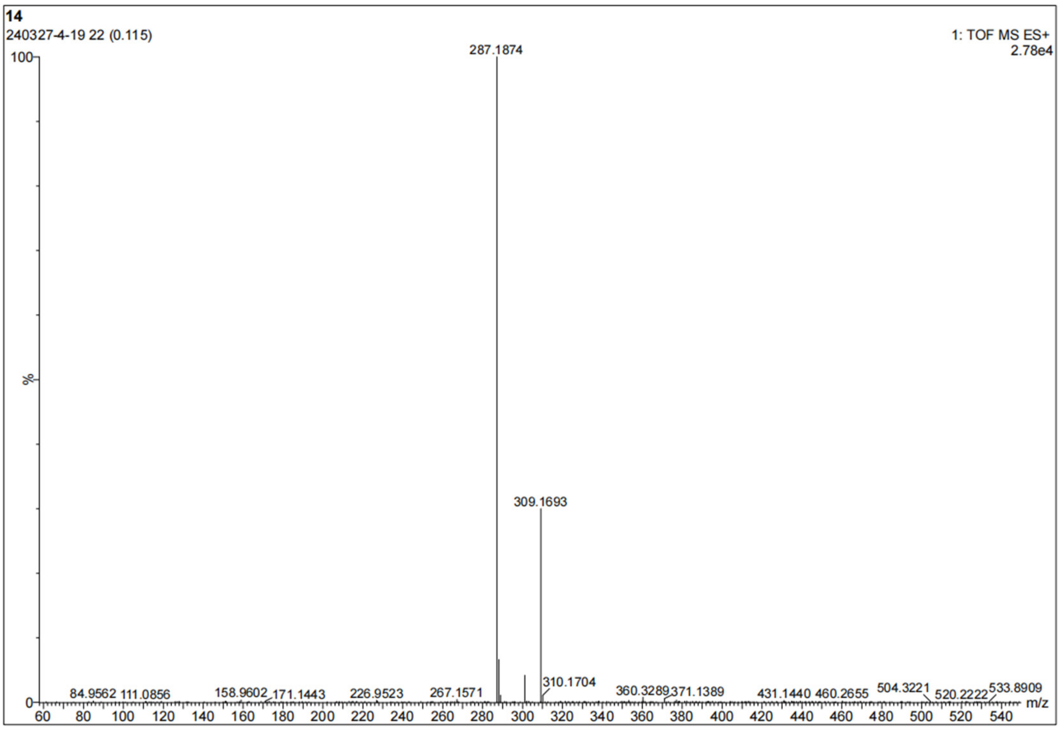

Figure S21-3. HRMS spectrum of compound 9f.

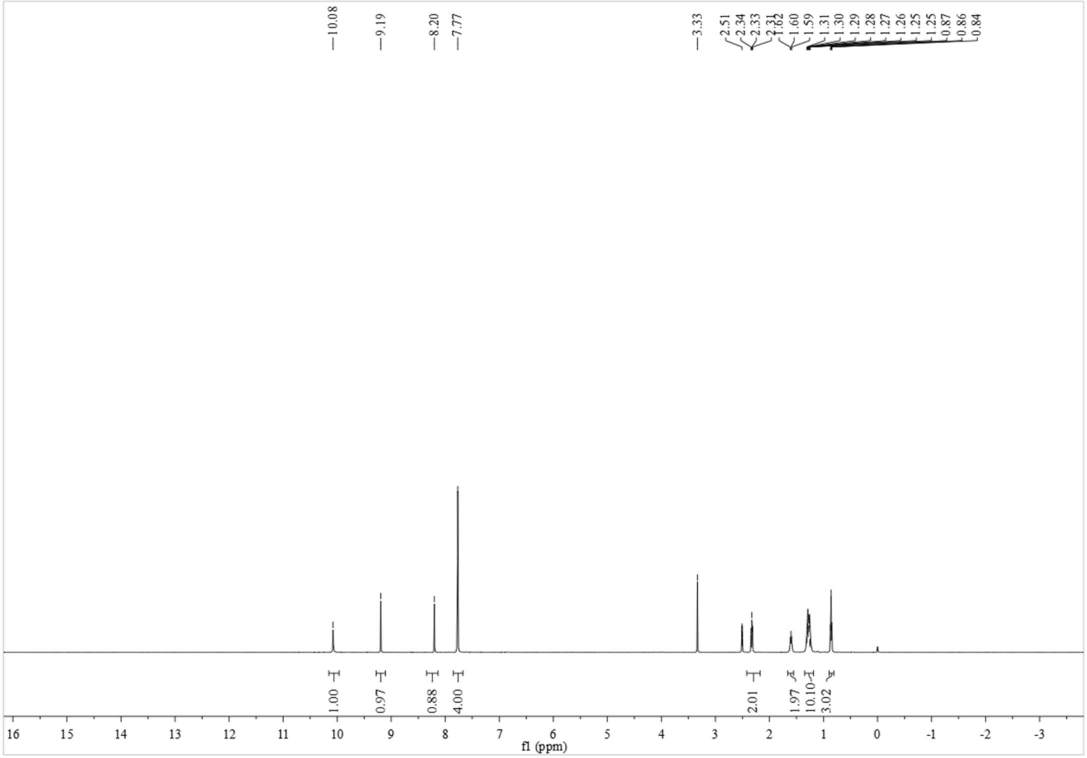

Figure S22-1. ^{1}H-NMR spectrum of compound 9g.

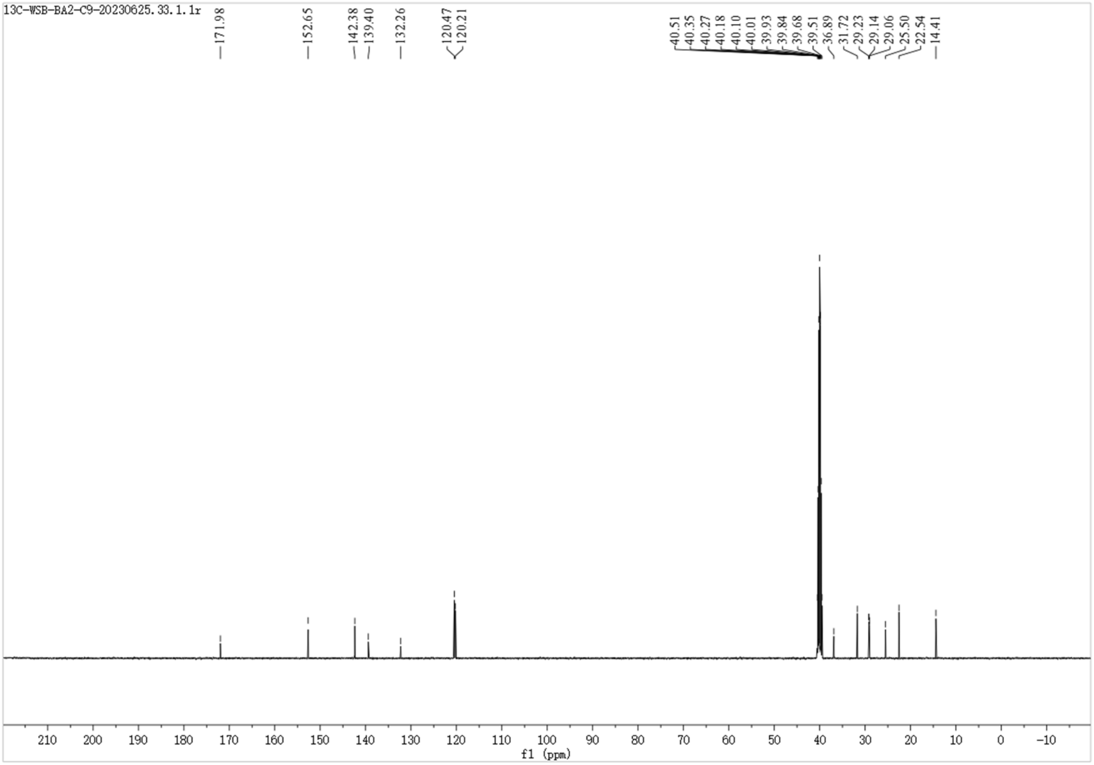

Figure S22-2. ^{13}**C-NMR spectrum of compound 9g.**

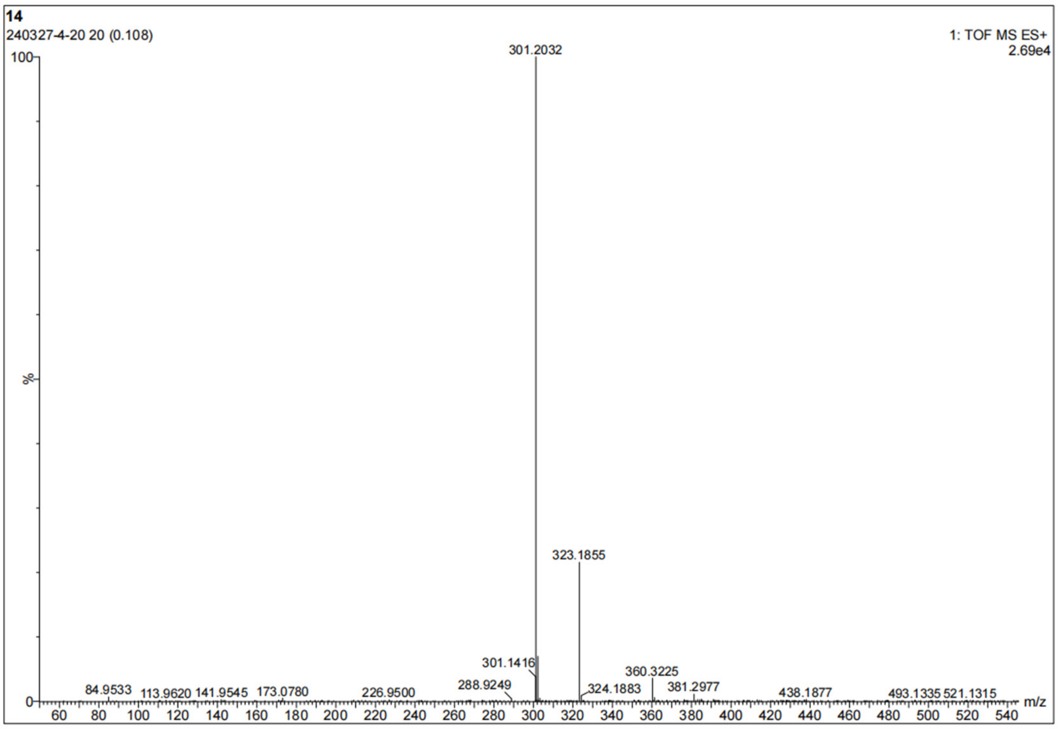

Figure S22-3. HRMS spectrum of compound 9g.

HPLC spectrum of the most promising compounds **6f** and **6l**.

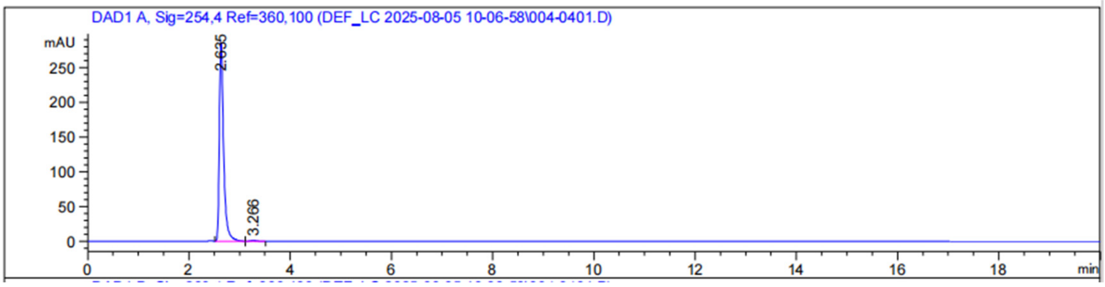

#	[min]		[min]	[mAU*s]	[mAU]	%
1	2.635	BB	0.0890	1686.39307	285.18253	99.5123
2	3.266	BB	0.1002	8.26457	1.23323	0.4877

Figure S23. HPLC **spectrum of compound 6f.**

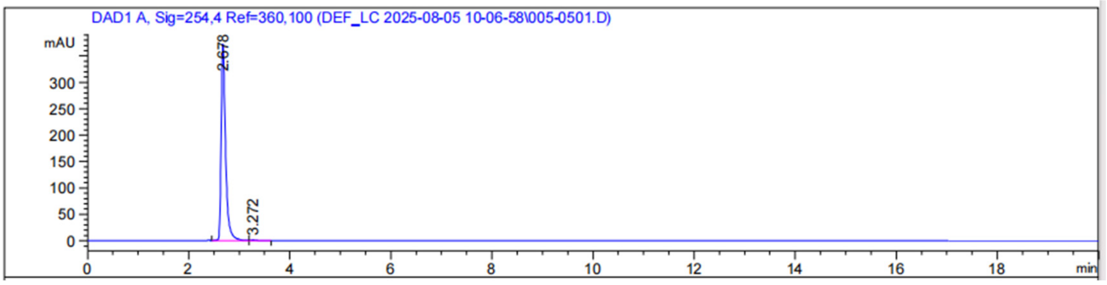

#	[min]		[min]	[mAU*s]	[mAU]	%
1	2.678	VV	0.0904	2254.81836	373.66208	99.5202
2	3.272	VB	0.1156	10.87085	1.35448	0.4798

Figure S24. HPLC **spectrum of compound 6l.**
